# Synthesis, molecular dynamics, and antimicrobial evaluation of peptide-antibiotic conjugates designed for dual-mechanism pathogen inhibition

**DOI:** 10.3389/fphar.2026.1927384

**Published:** 2026-09-17

**Authors:** Denisa Leonte, Clemence Fartaoui, Ioana Ionuț, Róbert Tőtős, Gabriel Marc, Laurian Vlase, Gheorghe-Adrian Martău, Dan Cristian Vodnar, Ovidiu Crişan

**Affiliations:** 1 Department of Organic Chemistry, “Iuliu Hațieganu” University of Medicine and Pharmacy, Cluj-Napoca, Romania; 2 Department of Pharmaceutical Chemistry, “Iuliu Hațieganu” University of Medicine and Pharmacy, Cluj-Napoca, Romania; 3 Enzymology and Applied Biocatalysis Research Center, Faculty of Chemistry and Chemical Engineering, Babeş-Bolyai University, Cluj-Napoca, Romania; 4 Department of Pharmaceutical Technology and Biopharmacy, “Iuliu Hațieganu” University of Medicine and Pharmacy, Cluj-Napoca, Romania; 5 Institute of Life Sciences, University of Agricultural Sciences and Veterinary Medicine, Cluj-Napoca, Romania; 6 Faculty of Food Science and Technology, University of Agricultural Sciences and Veterinary Medicine, Cluj-Napoca, Romania

**Keywords:** antibacterial activity, antifungal activity, antimicrobial peptide-drug conjugates, antimicrobial peptides, metronidazole-conjugated peptide, norfloxacin-conjugated peptide

## Abstract

**Introduction:**

In the context of rapid rise of resistant pathogen strains, antimicrobial peptides (AMPs) represent promising scaffolds that complement conventional antibiotics, especially given the specific toxicities that limit classic drugs like fluoroquinolones and nitroimidazoles. AMP monotherapy faces major drawbacks, primarily due to inherent host-cell toxicity and a short half-life.

**Methods:**

To overcome these limitations, this study details the design and synthesis of dual-mechanism prodrugs by conjugating the membrane-active AMPs scaffolds of urechistachykinin I and decoralin to norfloxacin and metronidazole via a pathogen-cleavable ester linker. To minimize off-target toxicity, both AMP sequences were synthesized with a C-terminal carboxyl group, as these variants exhibit negligible hemolytic and neurotoxic activity compared to their amidated analogues. The designed antibiotic-peptide conjugates were synthesized on solid support starting from the C-terminus, followed by N-terminal chain elongation to attach the antibiotics. Specific derivatization methods ensured the covalent ester linkage of norfloxacin and metronidazole to the N-termini of urechistachykinin I (resulting in conjugates **1a** and **1b**) and decoralin (resulting in conjugates **2a** and **2b**). Structural behavior was assessed by 200 ns molecular dynamics simulations in water. Antimicrobial activity was evaluated by broth microdilution against Gram-negative, Gram-positive bacteria and fungi, and compared to parent peptides and reference antibiotics as positive controls.

**Results:**

All conjugates exhibited increased flexibility and solvent exposure relative to their parent peptides, as evidenced by increased RMSD, radius of gyration, H-bonding with water and higher solvent accessible surface area. In vitro screening identified **1a**, **2a** and **2b** as the most active conjugates. Norfloxacin conjugate **1a** exhibited potent activity against *Escherichia coli* ATCC 8739 (MIC 0.56 μM) and *Candida parapsilosis* (MIC 2.26 μM). Norfloxacin conjugate **2a** showed broad-spectrum inhibition, including *Escherichia coli* ATCC 8739 (MIC 8.64 μM), *Salmonella enterica* ATCC BAA-2162 (MIC 8.64 μM) and *Listeria* *monocytogenes* DSMZ 115292 (MIC 4.32 μM), with improved activity over unmodified decoralin. Metronidazole conjugate **2b** was active against *Listeria monocytogenes* DSMZ 115292 (MIC 11.86 μM) and *Enterococcus faecalis* ATCC 29212 (MIC 23.12 μM).

**Discussion:**

These in vitro assays demonstrate retained peptide membrane-disrupting properties and synergistic effects with the antibiotics. The dual-mechanism design represents a promising strategy to reduce toxicity and overcome antimicrobial resistance.

## 1 Introduction

The progressive rise of multidrug-resistant pathogens poses a critical threat to global health, creating therapeutic challenges for currently established antibiotic therapy protocols. While traditional antibiotics remain fundamental in clinical practice, their diminishing margins of efficacy impose the development of innovative therapeutic strategies.

In this context, norfloxacin and metronidazole serve as key scaffold molecules for structural modulations and prodrug development. Despite their potent antimicrobial activity, these chemotherapeutics exhibit severe side effects: norfloxacin is linked to fluoroquinolone-induced tendinopathy and cartilage damage, whereas metronidazole can cause peripheral neuropathy and gastrointestinal side-effects. Structural modulations aim to optimize their therapeutic index, mitigate toxicity, and extend their antimicrobial spectrum against resistant strains. Although research in this specific direction remains relatively sparse, recent strategies to enhance their pharmacological and pharmacotoxicological profiles include chemical conjugation with distinct pharmacophores for synergistic action (Akgün et al., 2025; Li et al., 2022), macromolecular prodrug engineering for targeted delivery (Mura et al., 2011; Roseeuw et al., 2003) and amino acid/peptide functionalisation for enhanced microbial inhibition (Kamal El-Sagheir et al., 2023).

The limited studies available indicate that macromolecular engineering combined to peptide chemistry has proven effective in optimizing the pharmacokinetics of these agents. For instance, Roseeuw et al. (2003) successfully conjugated norfloxacin to dextran backbones via lysosomal-cleavable peptide spacers (e.g., Gly-Phe-Gly-Gly or Gly-Phe-Ala-Leu) to facilitate controlled intracellular drug release. By incorporating mannose residues, these conjugates enabled precise macrophage targeting, thereby maximizing *in vivo* efficacy against intracellular mycobacteria (Roseeuw et al., 2003). Similarly, metronidazole, a frontline agent for anaerobic pathogens infectious diseases and trichomoniasis, giardiasis, and amoebiasis (Patel et al., 2021), has been adapted for colon-targeted delivery. Metronidazole acts as a prodrug that undergoes intracellular activation only after its passive diffusion across the bacterial cell wall. The cytotoxicity of metronidazole is strictly governed by the bioreductive activation of its nitro group, a process mediated by specific proteins such as ferredoxin, found exclusively in anaerobic environments. This single-electron reduction converts the nitro group into highly reactive nitro radical intermediates, which directly interact with the microbial DNA, inhibiting nucleic acid synthesis and ultimately driving cell death (Patel et al., 2021). To mitigate its documented gastrointestinal side effects and to ensure its colon targeted delivery, Mura et al. (2011) developed chitosan-based polymeric prodrug formulations using succinyl and glutaryl spacers. These hydrophilic macromolecular chains restricted drug release to the colon through enzymatic cleavage, with the succinyl conjugate exhibiting a slower hydrolysis rate ideal for sustained delivery (Mura et al., 2011).

Beyond polymer-based carriers, modern optimization strategies focus on direct molecular functionalization. From a synthetic perspective, the primary hydroxyl group of metronidazole and the carboxyl group of norfloxacin serve as versatile anchors for the development of novel small-molecule hybrids, yielding new chemical entities with enhanced properties while preserving their essential pharmacophore cores (Akgün et al., 2025; Li et al., 2022). Conjugating norfloxacin with specific amino acid derivatives has been shown to enhance antibacterial and antimycobacterial potency, while simultaneously disrupting cell wall synthesis and membrane integrity (Kamal El-Sagheir et al., 2023).

In the continuation of this conjugation strategy, the use of antimicrobial peptides (AMPs) as conjugate partners has gained significant attention. Nevertheless, literature regarding norfloxacin and metronidazole peptide conjugates is still highly limited, in contrast with other therapeutic agents. AMPs have emerged as highly promising alternatives to conventional drugs - either as autonomous therapeutic entities or as covalent conjugate partners - owing to their broad-spectrum activity and exceptionally low potential for resistance development. Unlike traditional drugs that target specific intracellular enzymes, AMPs typically function by physically disrupting bacterial membrane bilayers, a mechanism that makes structural adaptation difficult for pathogens. Among these, cationic membrane-active AMPs, such as urechistachykinin I (LRQSQFVGSR) (Sung et al., 2008) and decoralin (SLLSLIRKLIT) (Konno et al., 2007) are of particular interest due to their high electrostatic affinity for negatively charged bacterial surfaces. Consequently, combining the precise target selectivity of fluoroquinolones or nitroimidazoles with the membrane-disrupting capabilities of these cationic peptide sequences represent a powerful frontier in the design of next-generation antimicrobials with dual mechanism.

Urechistachykinin I (LRQSQFVGSR) belongs to the tachykinin-related peptide family, characterized by the conserved FXGXR-amide motif, and shares high structural homology with vertebrate tachykinins (Mao et al., 2024). Although this peptide exhibits potent, broad-spectrum membrane-disruptive activity against various pathogens, its clinical translation is strictly constrained by its neurotoxicity. Mechanistic studies indicate that the C-terminal amide (-CONH_2_) moiety is a rigid functional requirement for activation of the urechistachykinin receptor (UTKR), a seven-transmembrane G-protein-coupled receptor that mediates calcium-dependent signal transduction upon binding of urechistachykinins I–V and VII (Kawada et al., 2002), which is directly responsible for these neurological side effects (Mao et al., 2024). While deleting this amide or converting it to a free carboxylate completely abolishes receptor signaling and associated neurotoxicity, it simultaneously diminishes the peptide’s antimicrobial potency.

Decoralin (SLLSLIRKLIT) is a linear, cationic, α-helical antimicrobial peptide (AMP) originally isolated from the venom of the solitary eumenine wasp *Oreumenes decoratus*) (Konno et al., 2007). Its amphipathic secondary structure is governed by an architectural balance of hydrophobic (Leu, Ile) and basic (Arg, Lys) amino acid residues. Previous investigations into native decoralin demonstrated that its wild-type form, which possesses a free C-terminal carboxyl group (-COOH), displays negligible hemolytic activity. Conversely, synthetic incorporation of a C-terminal amide function (-CONH_2_) enhances its antimicrobial efficacy, but induces a significantly more pronounced hemolytic toxicity profile (Konno et al., 2007).

Despite their significant antimicrobial activity, the clinical advancement of these AMPs in therapy remains severely hindered by poor metabolic stability and systemic toxicities, such as hemolysis.

To overcome these physiological limitations, the development of covalent antimicrobial peptide-drug conjugates (PDCs) has emerged as a powerful therapeutic strategy (Gattu et al., 2023). Chemically linking urechistachykinin I or decoralin with potent small-molecule antibiotics yields a synergistic, dual-action architecture capable of simultaneously attacking distinct bacterial structures: physical membrane disruption via the cationic peptide backbone and precise intracellular target inhibition via the antibiotic payload.

Based on these considerations, the primary objective of this study was to design and synthesize a novel series of antibiotic-conjugated antimicrobial peptides designed to enhance pathogen eradication through a synergistic, dual mechanism of action. This approach merges the established antimicrobial efficacy of metronidazole and norfloxacin with the membrane-disrupting capabilities of the cationic AMPs scaffolds urechistachykinin I and decoralin.

The design of these PDCs relies on a multi-functional chemical spacer. The integration of a pathogen-cleavable ester linker ensures site-specific antimicrobial cargo release, as it undergoes selective hydrolysis by bacterial esterases within the microenvironment of infection. This approach maximizes local drug concentrations while minimizing off-target toxicity in patients (Peng et al., 2025). In addition to this ester linkage, we opted for the incorporation of a di-β-alanine (β-Ala-β-Ala) spacer between the peptide sequence and the antibiotic payload, based on well-established strategies in drug design. Non-proteinogenic β-amino acids linkers are widely recognized for their biological inertness and high resistance to premature proteolytic degradation, serving strictly as structural tethers to alleviate steric hindrance between the bulky peptide carrier and the cargo (Effendi et al., 2021).

Furthermore, to optimize the safety profiles of both the norfloxacin- and metronidazole-conjugated peptides, we opted for the peptide sequences bearing a free C-terminal carboxyl group (-COOH) to mitigate systemic hemolysis and neurotoxicity.

To validate the therapeutic potential of these newly engineered PDCs, their antimicrobial efficacy was screened against eleven bacterial strains strategically selected to represent diverse cell envelope topologies and major infectious threats. The inclusion of five Gram-negative strains, including *Escherichia coli*, *Salmonella enterica*, and the opportunistic pathogen *Pseudomonas aeruginosa*, serves to validate the ability of the conjugates to traverse the asymmetric lipopolysaccharide outer membrane. Six Gram-positive strains (*Staphylococcus aureus*, *Staphylococcus epidermidis*, *Enterococcus faecalis*, *Streptococcus pyogenes*, *Bacillus subtilis*, and the intracellular pathogen *Listeria monocytogenes*) were selected to evaluate peptide interactions with thick, teichoic acid-rich peptidoglycan layers. This comprehensive antimicrobial profiling is essential to determine whether N-terminal modulations and C-terminal carboxyl retention successfully confer broad-spectrum bactericidal activity without compromising target selectivity.

## 2 Results

### 2.1 Design and synthesis of antibiotic-conjugated antimicrobial peptides

The design of norfloxacin-conjugated peptides **1a**, **2a** and metronidazole-conjugated peptides **1b**, **2b** aimed to preserve or to enhance antimicrobial potency and selectivity, while expanding the antimicrobial activity spectrum and minimizing the systemic toxicities of both the antibiotic moieties and the AMPs, urechistachykinin I and decoralin (Scheme 1).

**SCHEME 1:**
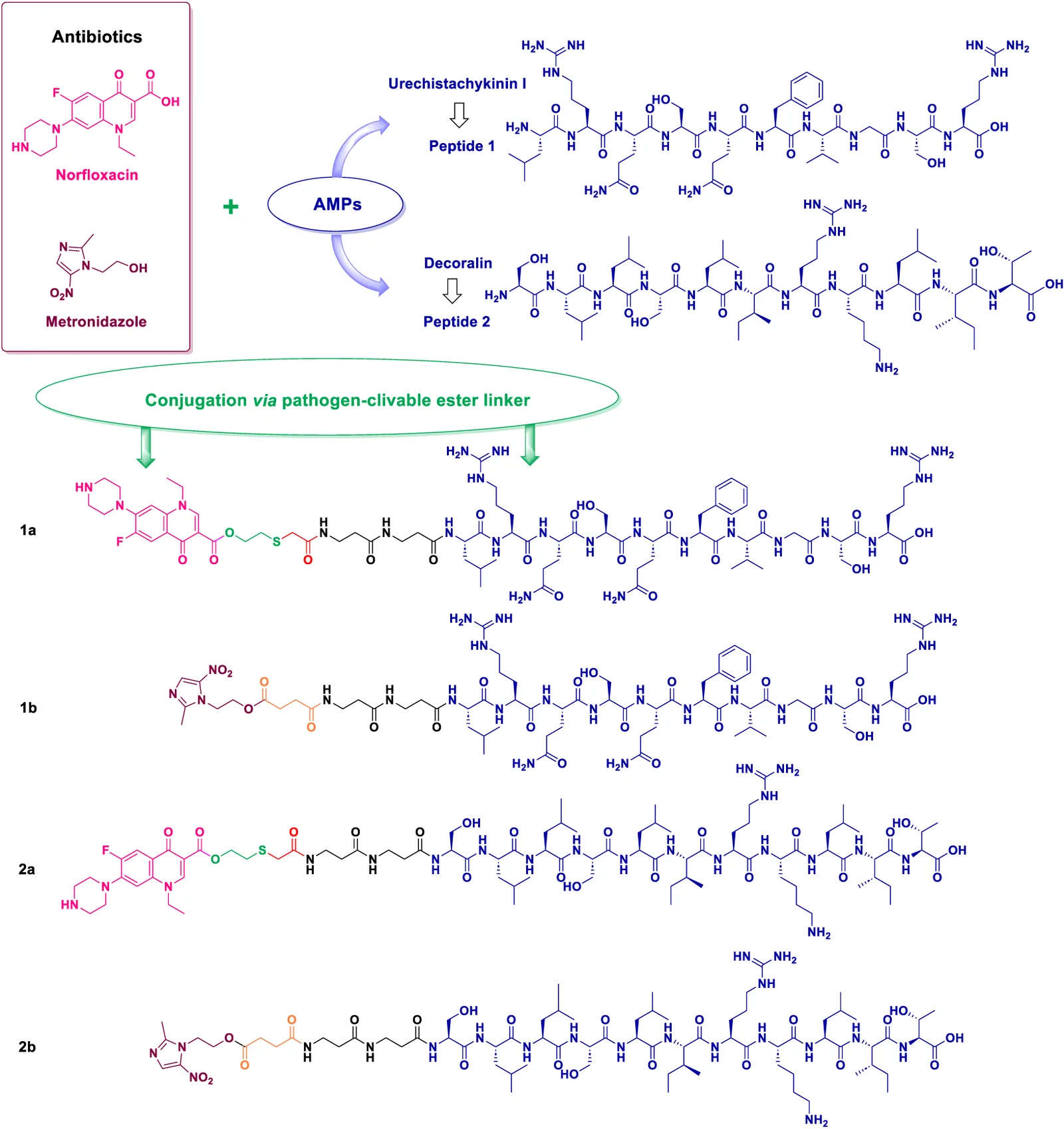
Schematic design of antibiotic-AMP conjugates via pathogen-cleavable ester linkers.

The synthesis of peptide-drug conjugates based on urechistachykinin I (LRQSQFVGSR) and decoralin (SLLSLIRKLIT) sequences was performed by standard SPPS using the Fmoc/tBu strategy, using 2-chlorotrityl chloride resin as solid support. To ensure that the conjugation does not compromise the structural amphipathic nature or the net positive charge of the peptides, all Fmoc-amino acids were protected at their side-chains with orthogonal protecting groups. We opted for a N-terminal conjugation of peptide sequences by chain elongation on solid support using the same Fmoc/tBu protocol. While the fully protected peptide remained anchored to the solid resin, the N-terminal Fmoc protective group was selectively removed, followed by chain elongation with two β-alanine units.

Specific chemical derivatization of norfloxacin and metronidazole was necessary in order to ensure covalent attachment of these molecules to peptide sequences by an ester-type bond.

#### 2.1.1 Modification and conjugation strategy for norfloxacin

To preserve the full antibacterial potency of norfloxacin upon its release, the conjugation strategy was strategically directed away from the piperazine. According to literature data, the free secondary amine of piperazine is critical for targeted binding to DNA gyrase and topoisomerase IV, as well as for outer membrane permeation (Aldred et al., 2014; Domagala, 1994). Modifying this amine frequently leads to a significant loss of antibiotic activity. Consequently, in our design, we opted for the covalent attachment of norfloxacin to the peptide sequence through an ester bond involving its C-3 carboxyl group. Prior to the esterification step on solid support, the secondary amine group of norfloxacin was temporarily protected by *tert*-butoxycarbonyl (Boc)-derivatization by an adapted already reported protocol (Chen et al., 2023).

Following the removal of the N-terminal Fmoc protective group from the β-alanine linker, chloroacetic acid was coupled to the free N-terminal amine on the solid support in the presence of N,N′-diisopropylcarbodiimide (DIC) as activating agent. The resulting chloroacetylated resin-bound peptide suspension was allowed to react with 2-mercaptoethanol to form a thioether linkage via *S*-alkylation in the presence of *N,N*-diisopropylethylamine (DIPEA). The free hydroxyl group of the newly formed hydroxyethylthioether moiety was then esterified with *N*-*tert*-butoxycarbonyl-norfloxacin, which was pre-activated with *O*-(Benzotriazol-1-yl)-*N,N,N′,N′*-tetramethyluronium hexafluorophosphate (HBTU) in the presence of 4-*N,N*-dimethylaminopyridine (DMAP) and DIPEA. *N*-Methylpyrrolidone (NMP) was utilized as the solvent due to the low solubility of *N*-Boc-norfloxacin in other common organic solvents.

#### 2.1.2 Modification and conjugation strategy for metronidazole

Metronidazole was structurally modified in solution using a bifunctional spacer. This was achieved by a similar protocol previously reported, involving acylation of the primary alcohol group with succinic anhydride, in the presence of DMAP and triethylamine (TEA) (Mura et al., 2011; Vermeersch et al., 1985) to functionalize it with a pathogen-cleavable ester linker. The resulting metronidazole hemisuccinate monoester was subsequently activated with DIC at its newly introduced carboxyl group, allowing it to be covalently anchored directly to the resin-bound N-terminal amino group of the peptides by an amide function.

#### 2.1.3 Purity and structure confirmation

The purity of the final products was verified by RP-HPLC, and their structures were confirmed by HRMS analysis using a Q-TOF instrument in positive ESI mode. All synthesized compounds exhibited high mass accuracy, with deviations below 2 ppm relative to the calculated monoisotopic masses (Table 1). For the peptide conjugates, the detection of [M+2H]^2+^ and [M+3H]^3+^ ions aligns consistently with the presence of basic amino acid residues (Arg, Lys) in their sequences.

**TABLE 1:** HRMS data for norfloxacin- and metronidazole-conjugated peptides*.

Compound	Molecular formula	Monoisotopic mass	Calculated [M + H]^+^	Found [M + H]^+^	Error ppm	Calculated [M+2H]^2+^	Found [M+2H]^2+^	Calculated [M+3H]^3+^	Found [M+3H]^3+^
**1a**	C_76_H_116_FN_23_O_21_S	1737.8421	1738.8494	1738.8473	−1.19	869.9283	869.9296	580.2880	580.2880
**1b**	C_66_H_105_N_23_O_22_	1571.7805	1572.7878	1572.7867	−0.68	786.8975	786.8958	524.9341	524.9309
**2a**	C_84_H_141_FN_20_O_21_S	1817.0285	1818.0358	1818.0332	−1.42	909.5215	909.5246	606.6834	606.6859
**2b**	C_74_H_130_N_20_O_22_	1650.9669	1651.9742	1651.9771	1.77	826.4907	826.4926	551.3296	551.3291

* All masses are monoisotopic. Error ppm = [(obs-calc)/calc] × 106.

### 2.2 *In vitro* antimicrobial activity evaluation

#### 2.2.1 Antibacterial activity evaluation

To evaluate the antibacterial efficacy of the synthesized peptides and their antibiotic conjugates, a panel of eleven clinically relevant bacterial strains was selected. This panel encompasses five Gram-negative pathogens (*E. coli* ATCC 8739, *E. coli* ATCC 25922, *S. enterica* ATCC BAA-2162, *S. enterica* ATCC 14028, and the highly resistant *P. aeruginosa* ATCC 27853) and six Gram-positive pathogens (*E. faecalis* ATCC 29212, *S. epidermidis* ATCC 12228, *L. monocytogenes* DSMZ 115292, *B. subtilis* ATCC 1177, *S. pyogenes* ATCC 12344, and *S. aureus* ATCC 29213). This selection ensures a robust evaluation of the tested compounds against distinct bacterial cell envelopes, encompassing the highly permeable peptidoglycan layer of Gram-positive walls and the restrictive lipopolysaccharide outer membrane of Gram-negative bacteria.

The results summarized in Table 2 show the determined minimum inhibitory concentrations (MIC, in μM) of peptides **1**, **2** and their antibiotic conjugates **1a, 1b, 2a, 2b** against the tested pathogen microbial strains. Fold-change comparisons quantifying conjugate potency relative to the unconjugated parent peptides, and relative to the free reference antibiotics, are presented in Figures 1, 2, respectively, and are discussed in detail in Section 3.

**TABLE 2:** Heatmap summary of the minimum inhibitory concentrations (MIC, μM) determined for parent peptides **1** and **2**, their antibiotic conjugates (**1a**, **1b**, **2a**, **2b**), and the reference antibiotics norfloxacin, metronidazole, and fluconazole (below the dashed line), against Gram-negative and Gram-positive bacterial strains, yeasts, and fungi.

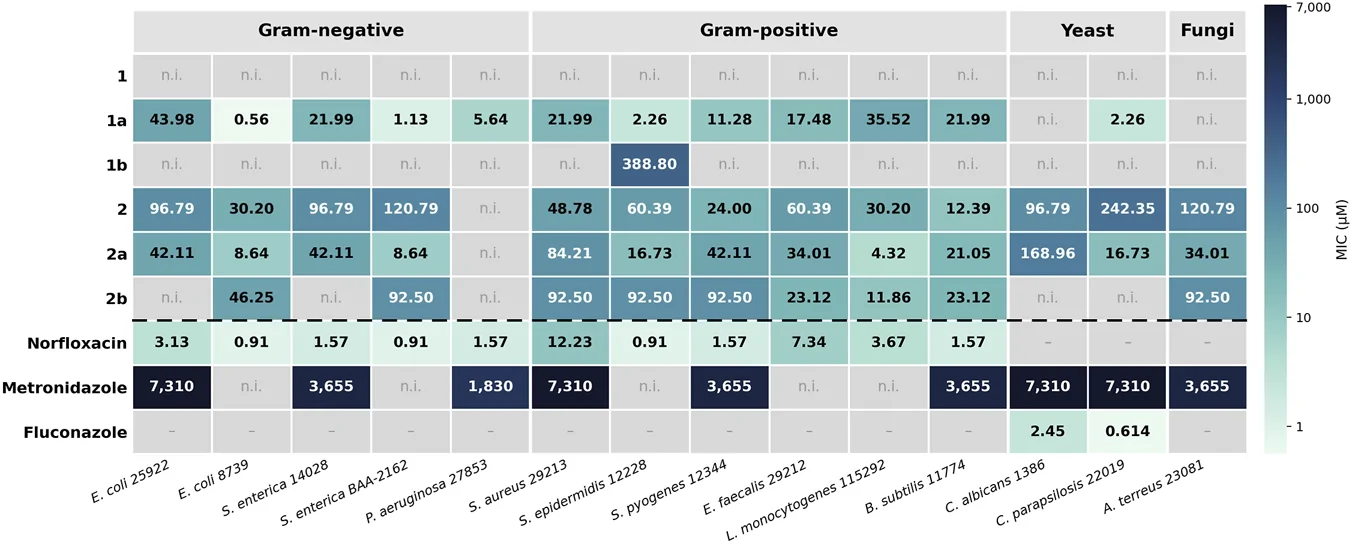

Color intensity reflects MIC on a logarithmic scale (lighter shading indicates greater potency); “n.i.” denotes no inhibition at the tested concentrations, and “–” denotes strains not tested against a given reference antibiotic. The final DMSO concentration was <1% v/v and was no-inhibitory.

**FIGURE 1:**
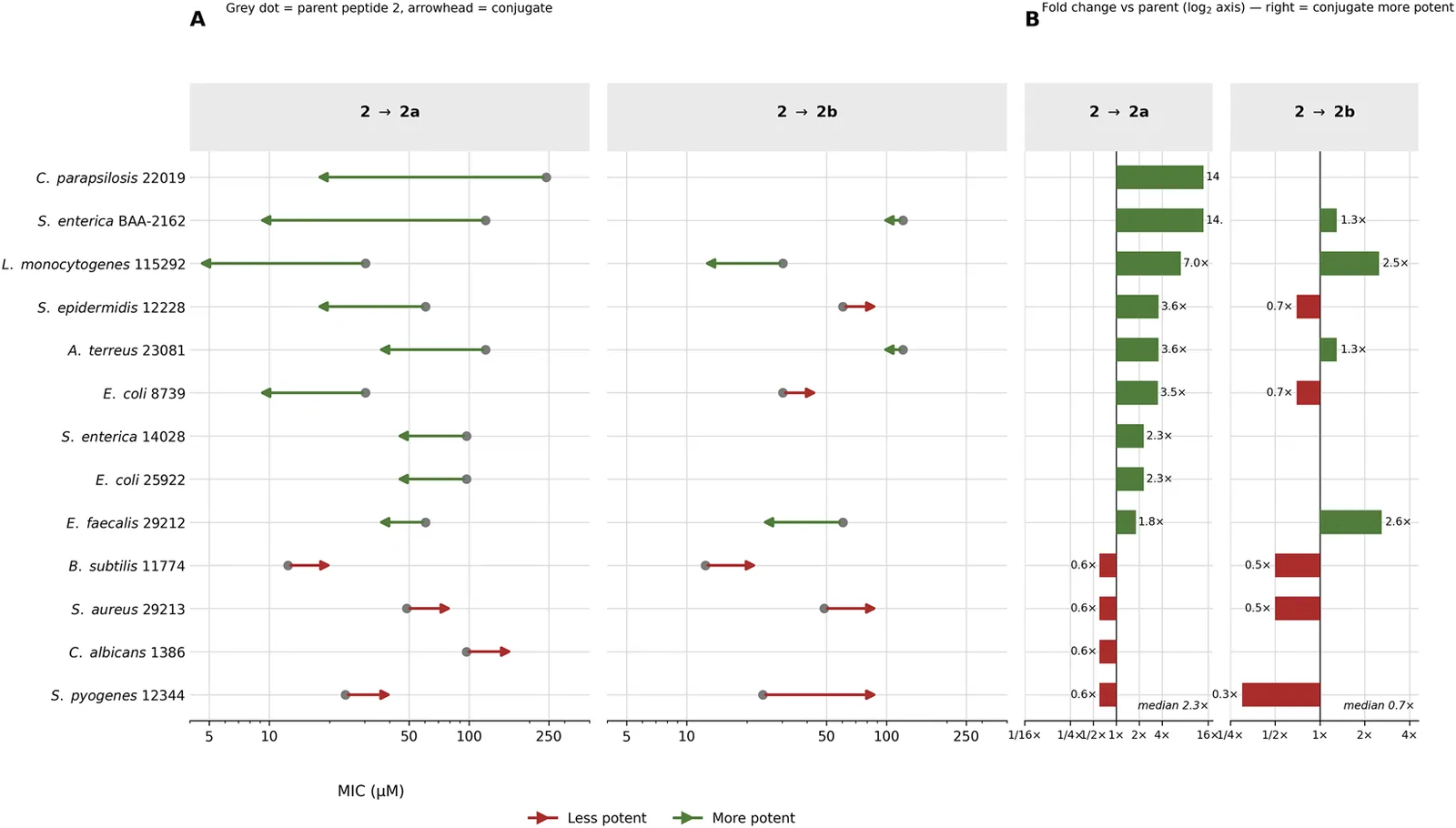
Fold-change in antimicrobial potency of conjugates **2a** and **2b** relative to the unconjugated parent peptide **2** (decoralin), across all strains tested. **(A)** MIC values (μM, log scale) for parent peptide **2** (grey dot) and the corresponding conjugate (arrowhead); arrow color indicates whether conjugation increased (green) or decreased (red) potency relative to the parent peptide. **(B)** Corresponding fold-change in potency, log_2_ scale; bars to the right of 1× indicate the conjugate was more potent than parent peptide **2**, bars to the left indicate reduced potency. Median fold-change is reported for each conjugate.

**FIGURE 2:**
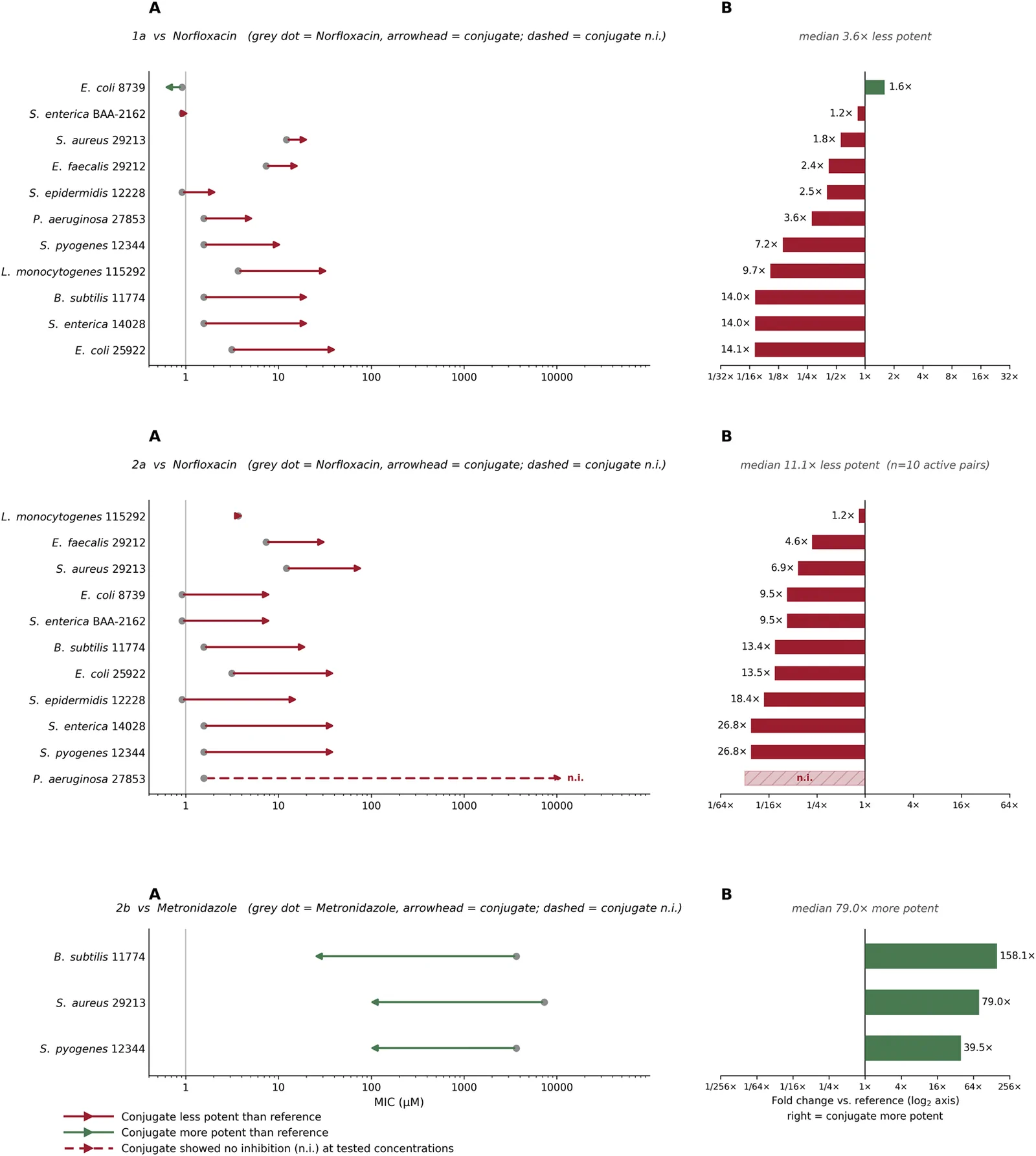
Head-to-head potency comparison between the peptide-antibiotic conjugates and the reference antibiotics (norfloxacin, metronidazole) used as positive controls in this study, across all strains for which the reference antibiotic was active. **(A)** shows MIC values (μM, log scale) for the conjugate (arrowhead) relative to the corresponding reference antibiotic (grey dot), and **(B)** shows the corresponding fold-change in potency on a log_2_ scale; bars extending to the right of 1 × indicate the conjugate outperformed the reference antibiotic, bars to the left indicate reduced potency. Solid arrows/bars denote strains where the conjugate showed measurable activity, colored green (conjugate more potent) or red (conjugate less potent). Dashed red arrows and hatched bars marked “n.i.” denote strains where the reference antibiotic was active but the conjugate showed no inhibition at the tested concentrations; these are excluded from the median fold-change reported in each **(B)** panel, together with the number of active pairs it is based on.

Norfloxacin-conjugated peptides **1a** and **2a** proved to exhibit lower MICs than the parent peptides **1** and **2** in most cases, suggesting that conjugation enhances activity.

When compared to the control norfloxacin, the conjugated peptide **1a** proved to be more active against *E. coli* ATCC 8739 (MIC 0.56 μM) and showed a comparable activity against *S. enterica* ATCC BAA-2162 (MIC 1.13 μM) and *S. aureus* ATCC 29213 (MIC 21.99 μM).

Although compound **2a** did not surpass the activity of free norfloxacin, this conjugated peptide showed good activity against the tested pathogenic bacterial strains with MICs between 4.32 and 84.21 μM. Instead, when compared to the parent peptide **2**, an enhanced antibacterial activity was observed for **2a** against Gram-negative bacterial strains *E. coli* ATCC 8739 (MIC 8.64 μM), *E. coli* ATCC25922 (MIC 42.11 μM), *S. enterica* ATCC BAA-2162 (MIC 8.64 μM) and *S. enterica* ATCC 14028 (MIC 42.11 μM), as well as against Gram-positive bacterial strains *E. faecalis* ATCC 29212 (MIC 34.01 μM), *S. epidermidis* ATCC 12228 (MIC 16.73 μM) and *L. monocytogenes* DSMZ 115292 (MIC 4.32 μM).

The metronidazole-conjugated peptides **1b** and **2b** displayed markedly distinct antibacterial profiles, thereby underscoring the critical influence of the peptide sequence on overall bioactivity. While conjugate **1b** exhibited negligible or completely absent activity across the majority of the tested strains, **2b** demonstrated, in some cases, enhanced antimicrobial efficacy relative to both free metronidazole and the parent peptide **2**. When active, the MIC values for **2b** (ranging from 11.86 to 92.50 μM) were substantially lower than those of metronidazole (MIC > 1830 μM). Compared to peptide **2**, **2b** showed improved potency against facultative anaerobic strains *S. enterica* ATCC BAA-2162 (from 120.79 μM to 92.50 μM), *E. faecalis* ATCC 29212 (from 60.39 μM to 23.12 μM), and *L. monocytogenes* DSMZ 115292 (from 30.20 μM to 11.86 μM) (Table 2).

#### 2.2.2 Antifungal activity evaluation

The antimicrobial efficacy of the parent peptides (**1** and **2**) and their respective peptide-drug conjugates (**1a**, **1b**, **2a**, and **2b**) was evaluated against a fungal panel comprising two yeast strains and one filamentous fungus: *Candida parapsilosis* ATCC 22019, *Candida albicans* ATCC 1386, and *Aspergillus terreus* DSMZ 23081. The determined MIC values are summarized in Table 2.

Parent urechistachykinin I analogue (peptide **1**) was completely inactive across all tested strains. Conversely, the parent decoralin (peptide **2**) exhibited moderate antifungal activity, presenting MIC values ranging from 96.79 μM against *C. albicans* to 242.35 μM against *C. parapsilosis*.

The norfloxacin-peptide conjugate **1a** demonstrated superior potency compared to both free norfloxacin and the parent peptide **1** specifically against *C. parapsilosis*, achieving a low MIC value of 2.26 μM. Furthermore, covalent linkage of norfloxacin to peptide **2** (conjugate **2a**) significantly enhanced antimycotic activity against *C. parapsilosis* (MIC = 16.73 μM) and *A. terreus* (MIC = 34.01 μM), while moderately augmenting efficacy against *C. albicans* (MIC = 168.96 μM).

In contrast, the metronidazole-conjugated variants **1b** and **2b** proved devoid of activity against the tested yeast strains. Notably, only the metronidazole-conjugate **2b** exhibited a moderately improved antifungal effect against *A. terreus* (MIC = 92.50 μM) relative to the parent peptide **2** (MIC = 120.79 μM).

### 2.3 Molecular dynamics simulation and structural analysis

To investigate the structural organization, conformational stability and dynamic behavior of the parent peptides (**1** and **2**) and how the covalent conjugation with metronidazole and norfloxacin (conjugates **1a–b** and **2a–b**) influences the 3D structure, the initial minimized structures of **1, 2** and their conjugates **1a–b** and **2a–b** in vacuum are presented in Figures 3A,C.

**FIGURE 3:**
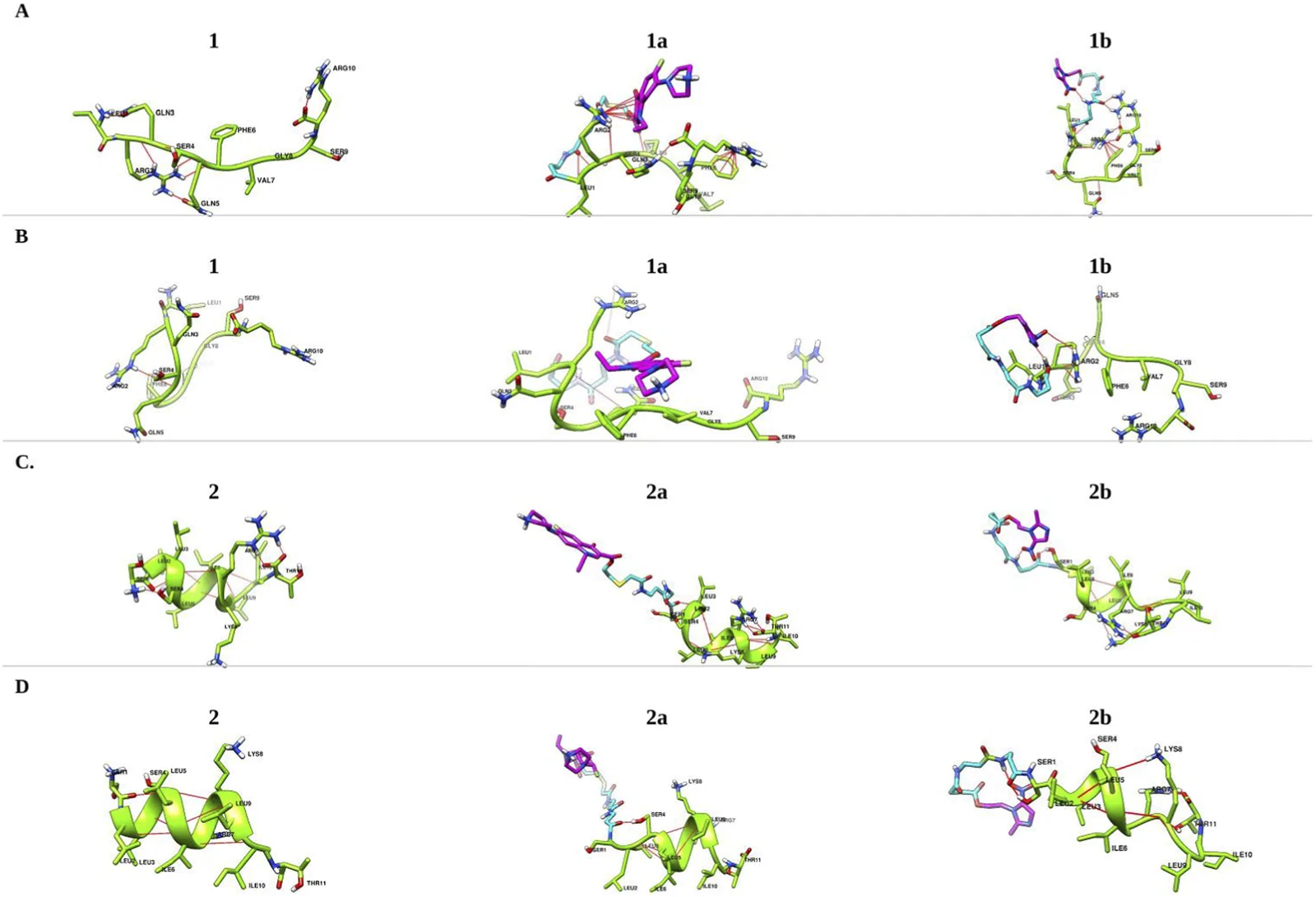
The 3D energy-minimized structures (vacuum) and the most populated conformer evolved over a 200 ns MD run (water), for antimicrobial peptides and their conjugates. **1/2** = parent peptides. **1a/2a** = norfloxacin conjugate. **1b/2b** = metronidazole conjugate. Metronidazole and norfloxacin are depicted in magenta, the peptide in green, and the linker in azure. Polar intramolecular interactions are depicted in red lines. **(A)** 3D structures of peptide 1 and conjugates 1a–b in vacuum (energy-minimized). **(B)** 3D structures of peptide 1 and conjugates 1a–b in water (200 ns MD, most populated conformer). **(C)** 3D structures of peptide 2 and conjugates 2a–b in vacuum (energy-minimized). **(D)** 3D structures of peptide 2 and conjugates 2a–b in water (200 ns MD, most populated conformer).

From a comparative point of view, the initial minimized structures obtained in vacuum and after solvent equilibration showed that conjugation with the antimicrobial moieties metronidazole and norfloxacin attachment to the peptides backbone through the designed linker induced structural rearrangements of the architecture of the parent peptides. The structures obtained in aqueous environment after equilibration suggest structural adaptation of the conjugates to solvent exposure, with the conjugated antimicrobial moieties which adopted different orientations relative to the peptide core, dependent on their structure (Figures 3B,D). As depicted in Figure 3B, while the nitro group of metronidazole was predicted to be involved in a hydrogen bond as acceptor from one of the peptides from the linker, the more complex norfloxacin moiety is predicted to be involved in π-cation interaction with the sidechain of Arg2 (**1a**), while in **2a**, the whole conjugate has a linear structure, with preservation of a partially helical-like backbone arrangement observed in the minimized structure of the parent peptide **2.**

The intramolecular hydrogen bonding analysis of the peptides and their conjugates (Supplementary Table S1) provides further insight into peptides stability. Peptide **1** had an average of 2.14 ± 1.57 intramolecular hydrogen bonds/nanosecond, whereas conjugation resulted in increased values of 2.85 ± 1.96 hydrogen bonds/nanosecond for **1a** and 3.09 ± 1.90 hydrogen bonds/nanosecond for **1b**. For peptide **2**, the native structure exhibited stronger internal hydrogen bonding (5.28 ± 1.74 hydrogen bonds/nanosecond), while the conjugates maintained comparable values (6.23 ± 1.84 hydrogen bonds/nanosecond for **2a** and 4.69 ± 1.56 hydrogen bonds/nanosecond for **2b**), but with no analogous trend when compared to **1a** and **1b**. These results might suggest that attachment of the antibiotic groups can promote the stabilization of the peptide backbone by promoting additional internal contacts with the antimicrobial conjugate that help preserve compact conformations during the simulation, but the RMSD of the peptide moiety increased in all cases, indicating the conjugation promoted a degree of destabilization of the parent peptide. Hydrogen bonding analysis demonstrated that all investigated structures maintained stable interactions with the solvent throughout the simulation (Supplementary Table S1). The chart plotting the intramolecular hydrogen bonding of the studied peptides during the molecular dynamics simulations is presented in Figure 4.

**FIGURE 4:**
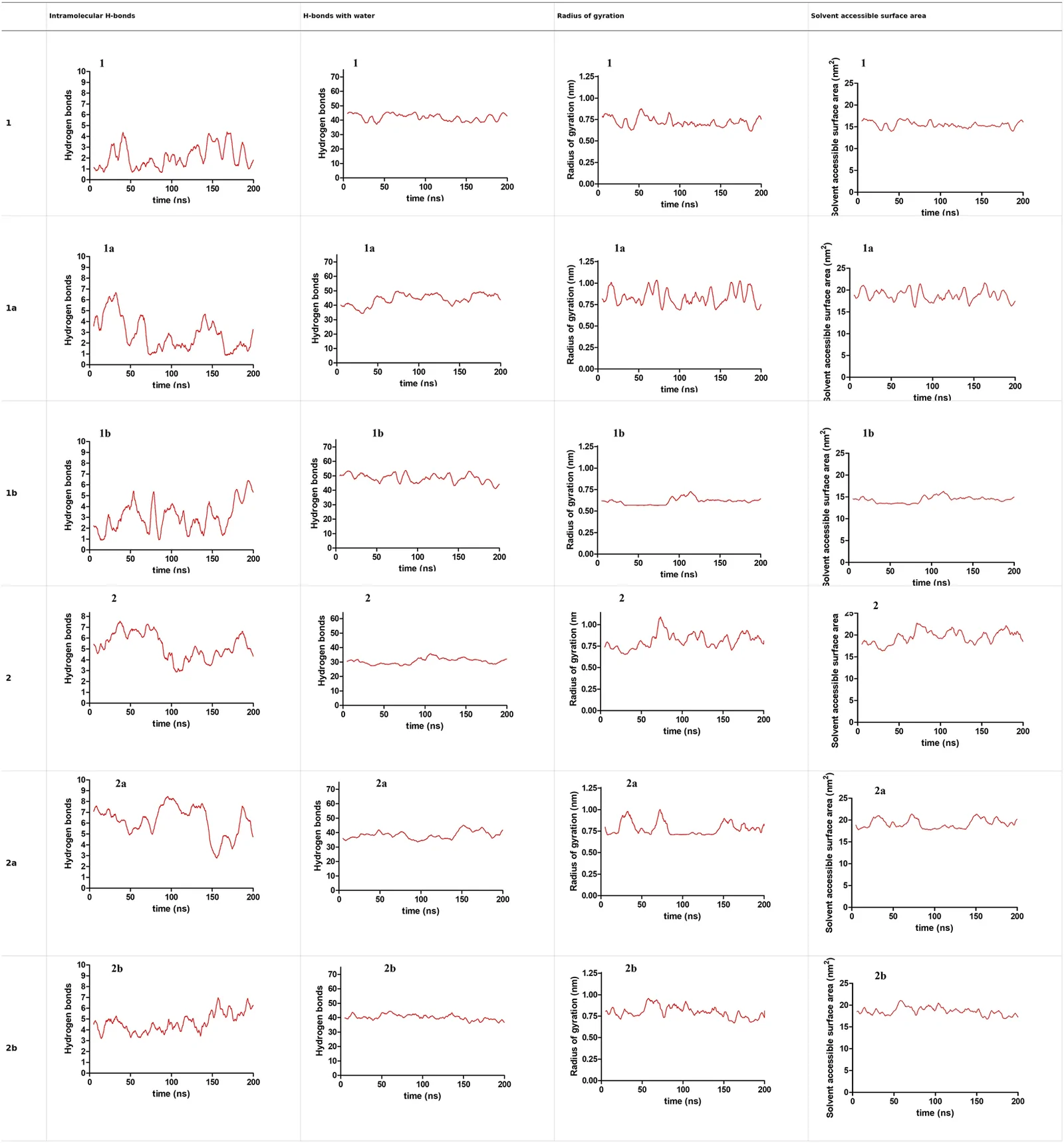
Time-course molecular dynamics metrics—intramolecular hydrogen bonds, hydrogen bonds with water, radius of gyration, and solvent-accessible surface area—over 200 ns, for peptides **1** and **2** and their conjugates (**1a, 1b, 2a, 2b**). Rows: compound. Columns: metric.

On the other hand, the parent peptide **1** displayed an average of 42.02 ± 4.34 hydrogen bonds with water, while conjugation increased these interactions to 44.13 ± 5.53 for **1a** and 48.74 ± 5.06 for **1b**. A similar trend was observed for peptide **2**, where the number of hydrogen bonds with water increased from 30.66 ± 3.35 for the unconjugated peptide to 38.43 ± 4.18 and 40.19 ± 4.06 for **2a** and **2b**, respectively. These results indicate that the antimicrobial moieties and the linker increase the interaction capacity of the conjugates with water, most likely due to additional hydrogen-bond donor/acceptor by the groups exposed to the solvent. The chart plotting the hydrogen bonding of the studied peptides with water during the molecular dynamics simulations is presented in Figure 4.

The conjugation of the two parent peptides **1** and **2** increased the average RMSD of the peptide moiety, with a higher effect on the **2a-b** derivatives. Taking into account the structural difference between parent peptides **1** and **2**, RMSD of the peptide **2**, which has a helical structure was 0.26 ± 0.10 nm, lower than of peptide **1** 0.42 ± 0.14 nm.

The average RMSD of peptide moiety of the conjugates when compared to the parent peptides **1** and **2** was increased in all four conjugates **1a-b** and **2a-b**, the amplitude of the change being higher for the 2 series derivatives.

The compactness of the studied peptides and their conjugates was also evaluated through the radius of gyration analysis. The trajectories showed relatively stable radius of gyration profiles for all structures (Figure 4), indicating the absence of major conformational change events during the 200 ns simulations. The conjugates followed the behavior of their corresponding parent peptides, demonstrating that the addition of the antimicrobial moieties did not cause large-scale structural expansion. Conjugation increased the overall molecular dimensions, as reflected by the higher radius of gyration values, yet the absence of progressive drift in the trajectories indicates that the larger conformations remained stable throughout the trajectory. The maintained compactness is consistent with the hydrogen-bonding results, where both solvent interactions and intramolecular contacts contribute to structural stabilization.

Solvent accessible surface area (SASA) analysis showed that all studied peptides maintained relatively constant solvent exposure during the simulations. Conjugation substantially increased the solvent-accessible surface area, reflecting the larger molecular size and increased solvent exposure of the antibiotic moieties, while the relatively stable SASA trajectories indicate preservation of conformational integrity during the simulations. The stable SASA profiles further support the conclusion that the conjugates maintain their overall conformation in aqueous conditions (Figure 4).

The free energy of solvation analysis revealed variable interactions between the compounds and the aqueous environment, for example, **2** had positive values, but its derivatives more favorable values (Figure 5). This observation for peptide **2** can be attributed to the helix structure, where the polar peptide bonds are oriented inside the helix and less to the external environment. Interestingly, the free energy of solvation had no correlation with the substituted conjugate, being also influenced by the parent structure of the peptide which was conjugated with. The Coulombic contribution was the dominant energetic component, indicating that electrostatic interactions between the studied peptides and solvent molecules play a major role in hydration. In particular, conjugates such as **1b** and **2a** showed highly favorable electrostatic contributions, consistent with their increased hydrogen bonding with water.

**FIGURE 5:**
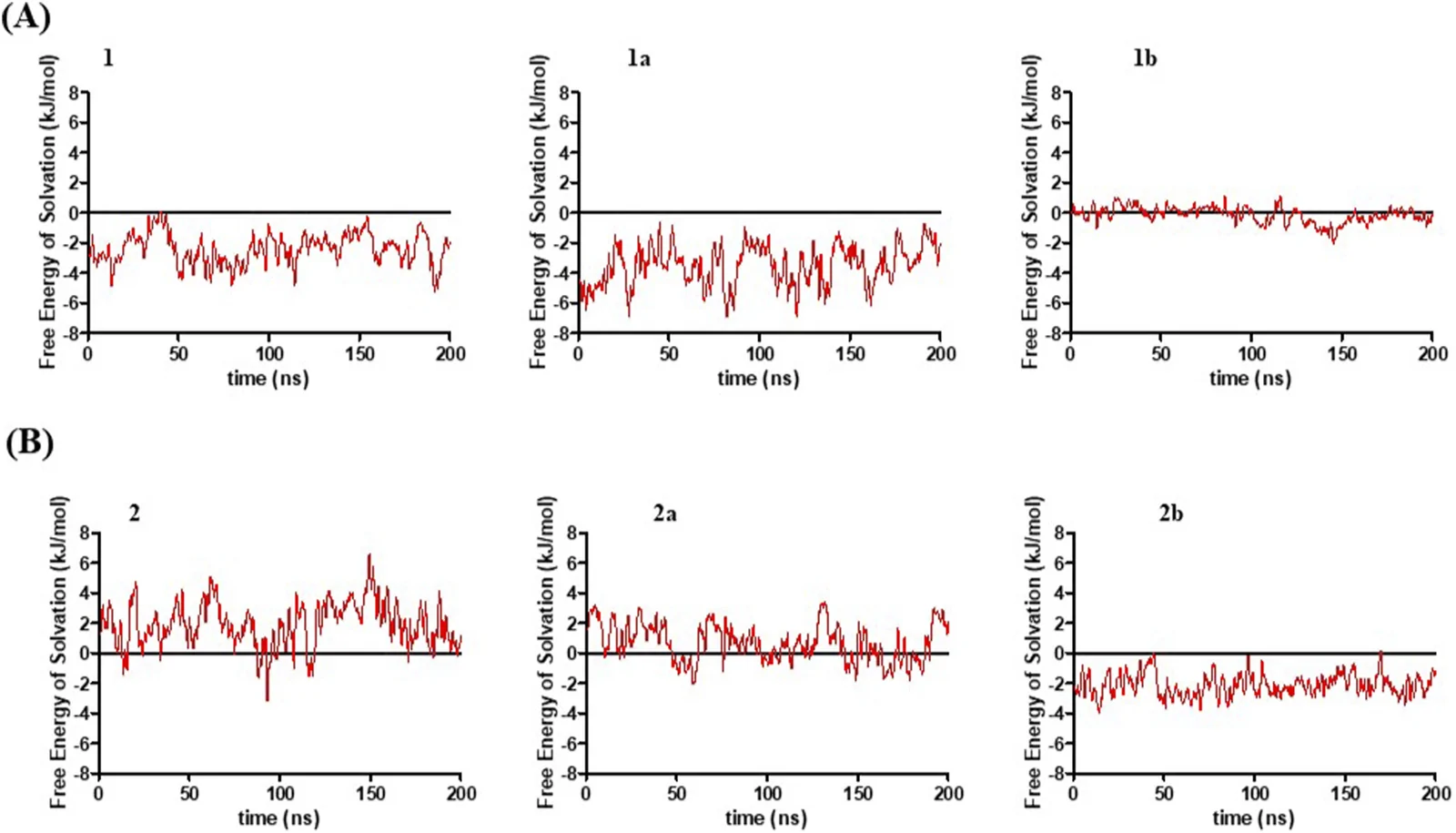
**(A)** Free energy of solvation of peptides **1, 1a, 1b** (1 ns moving average) in the molecular dynamics simulations. **(B)** Free energy of solvation of peptides **2, 2a, 2b** (1 ns moving average) in the molecular dynamics simulations.

Residue-level flexibility was evaluated by calculating the root mean square fluctuation (RMSF) of parent peptides backbone: α carbon atoms, oxygen atoms, nitrogen atoms, carbonyl carbon atoms and side chains (Figure 6). The RMSF profiles show that fluctuations are mainly localized at specific residues rather than being distributed across the entire peptide sequence. For peptide **1** and its derivatives, the central residues display relatively restricted motion, whereas terminal and side-chain regions show higher flexibility, which is expected for short peptides. Similar behavior was observed for peptide **2** and its conjugates. Importantly, conjugation with metronidazole or norfloxacin did not introduce excessive flexibility, indicating that the attached molecules are accommodated without disrupting the dynamic stability of the peptide framework.

**FIGURE 6:**
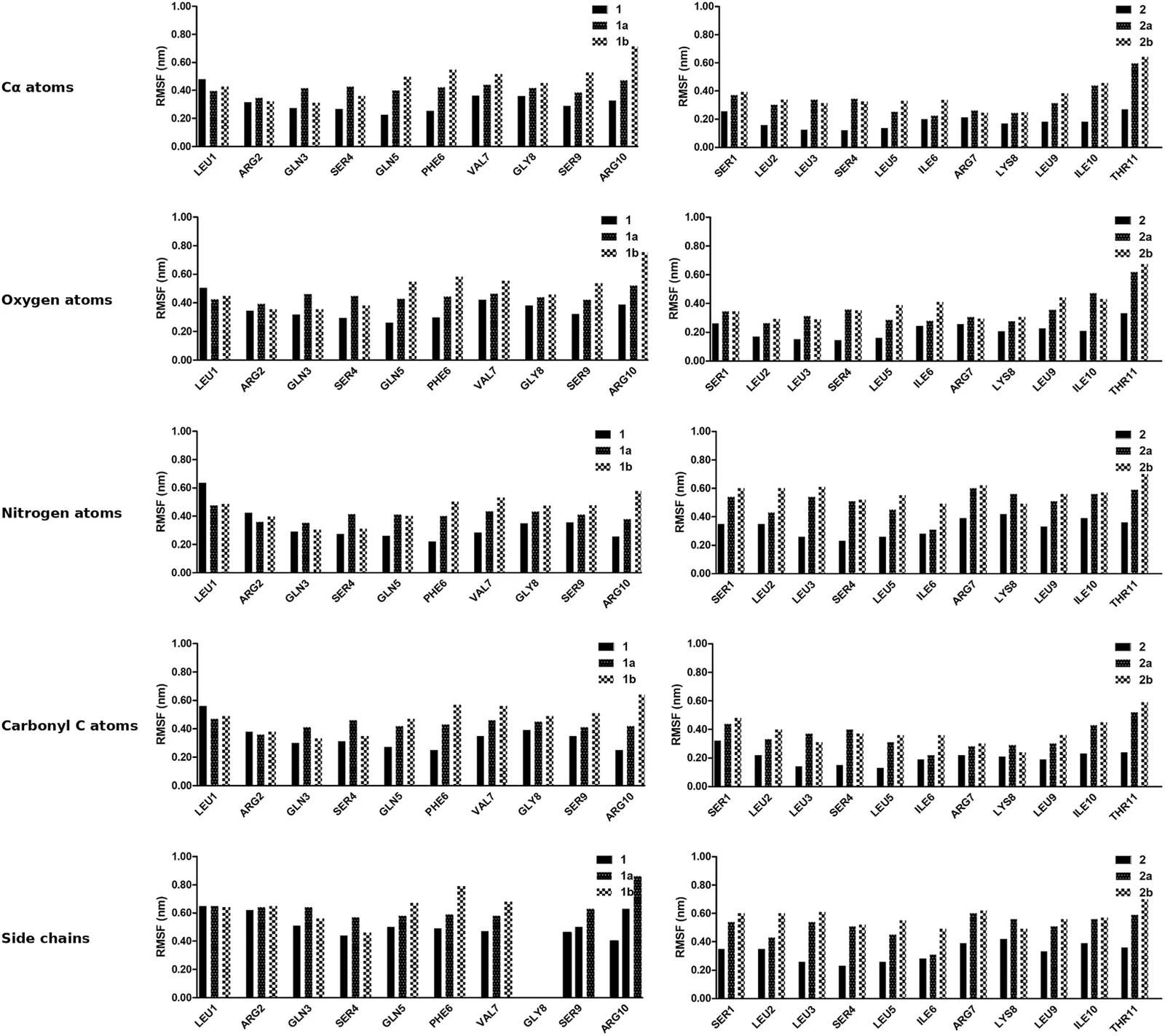
Root-mean-square fluctuation (RMSF, nm) per residue for the Cα, oxygen, nitrogen, and carbonyl carbon backbone atoms, and for side chains, of peptides **1, 2** and their conjugates (**1a, 1b, 2a, 2b**). Left column: peptide **1** series. Right column: peptide **2** series.

The interaction energy between the peptides and water further confirmed strong solvent stabilization. Peptide **2** showed stronger Coulombic interactions with water compared with peptide **1**, while conjugation modified these interactions depending on the antimicrobial moiety (Supplementary Table S2; Figure 7). For example, peptide conjugate **2a** displayed the most favorable total interaction energy among the peptide **2** derivatives, suggesting enhanced hydration after norfloxacin conjugation. The increased Lennard–Jones contributions observed for several conjugates indicate additional nonpolar and dispersion interactions introduced by the antimicrobial moieties. The increased solvent-accessible surface area of **1b** is accompanied by the largest number of water-mediated hydrogen bonds, indicating that solvent exposure directly contributes to enhanced hydration and electrostatic stabilization.

**FIGURE 7:**
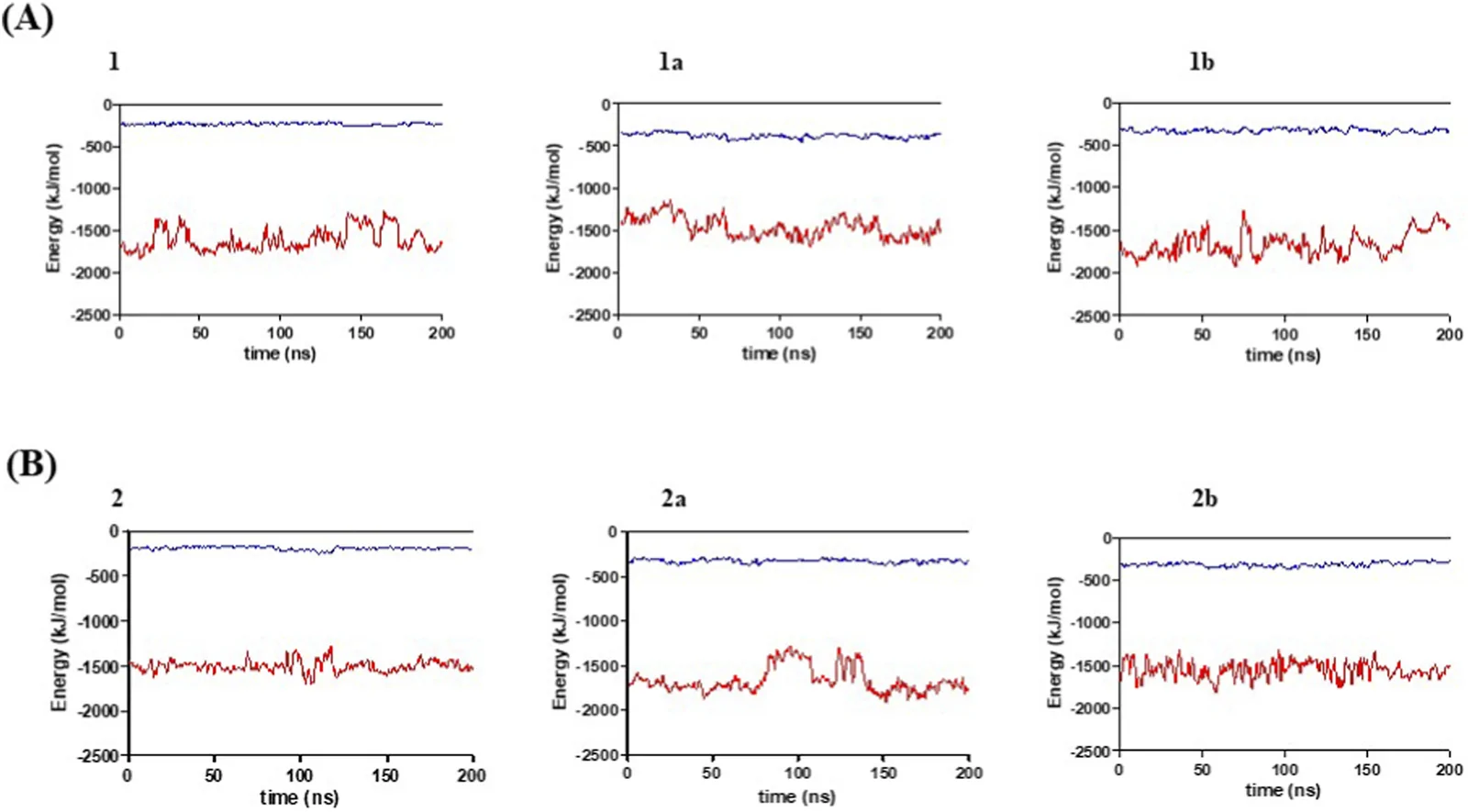
**(A)** The energy of interaction of the peptides **1, 1a, 1b** with water (1 ns moving average). **(B)** The energy of interaction of the peptides **2, 2a, 2b** with water (1 ns moving average).

Notably, conjugation with norfloxacin (**1a**) markedly reduced the intramolecular Coulombic interaction energy relative to the parent peptide **1** (−750.92 vs. −1683.16 kJ/mol), suggesting that norfloxacin moiety disrupted some of the initial electrostatic interactions network observed in peptide **1**, while for the metronidazole conjugates **1b** and **2b**, increased the Coulombic intramolecular interaction (Supplementary Table S3; Figure 8).

**FIGURE 8:**
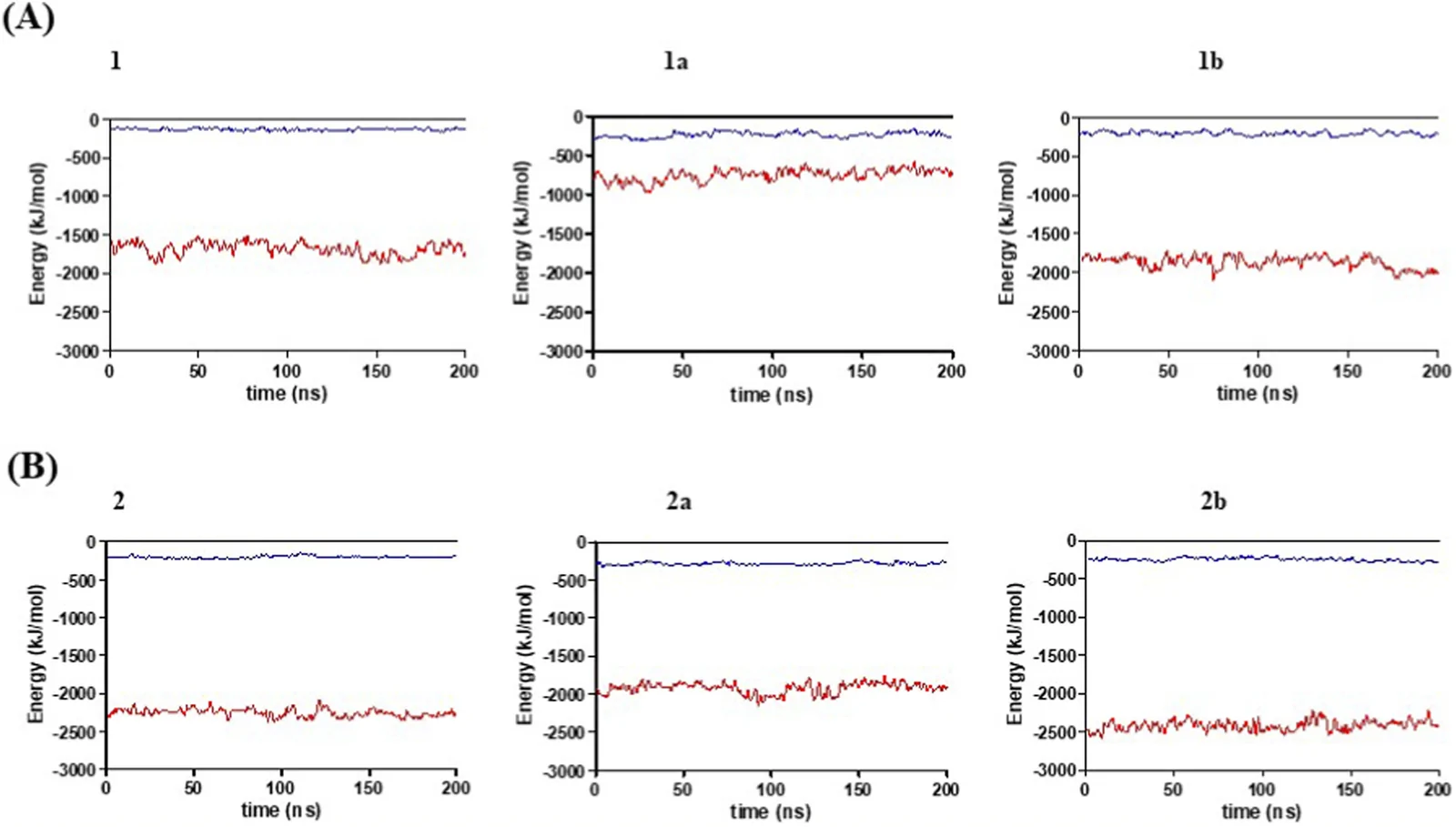
**(A)** The intramolecular energy of interaction of the peptides **1, 1a, 1b** (1 ns moving average). **(B)** The intramolecular energy of interaction of the peptides **2, 2a, 2b** (1 ns moving average).

The end-to-end distance analysis showed that the peptide backbones maintained stable dimensions throughout the trajectories. Peptides **1, 1a,** and **1b** displayed between the two ends average distances of 1.43 ± 0.47 nm, 1.39 ± 0.46 nm, and 1.25 ± 0.45 nm, respectively (Supplementary Table S4; Figure 9), indicating that conjugation slightly favored a more compact arrangement. In contrast, peptide **2** derivatives showed slightly larger distances (1.48 ± 0.23 nm for 2, 1.53 ± 0.21 nm for **2a**, and 1.62 ± 0.23 nm for **2b**), suggesting a more extended but still stable conformation (Supplementary Table S4; Figure 9). The distances between the antibiotic fragments and peptide ends remained within stable ranges, with a tendency for **2a** to maintain a constant state between 75 and 140 ns of the simulation.

**FIGURE 9:**
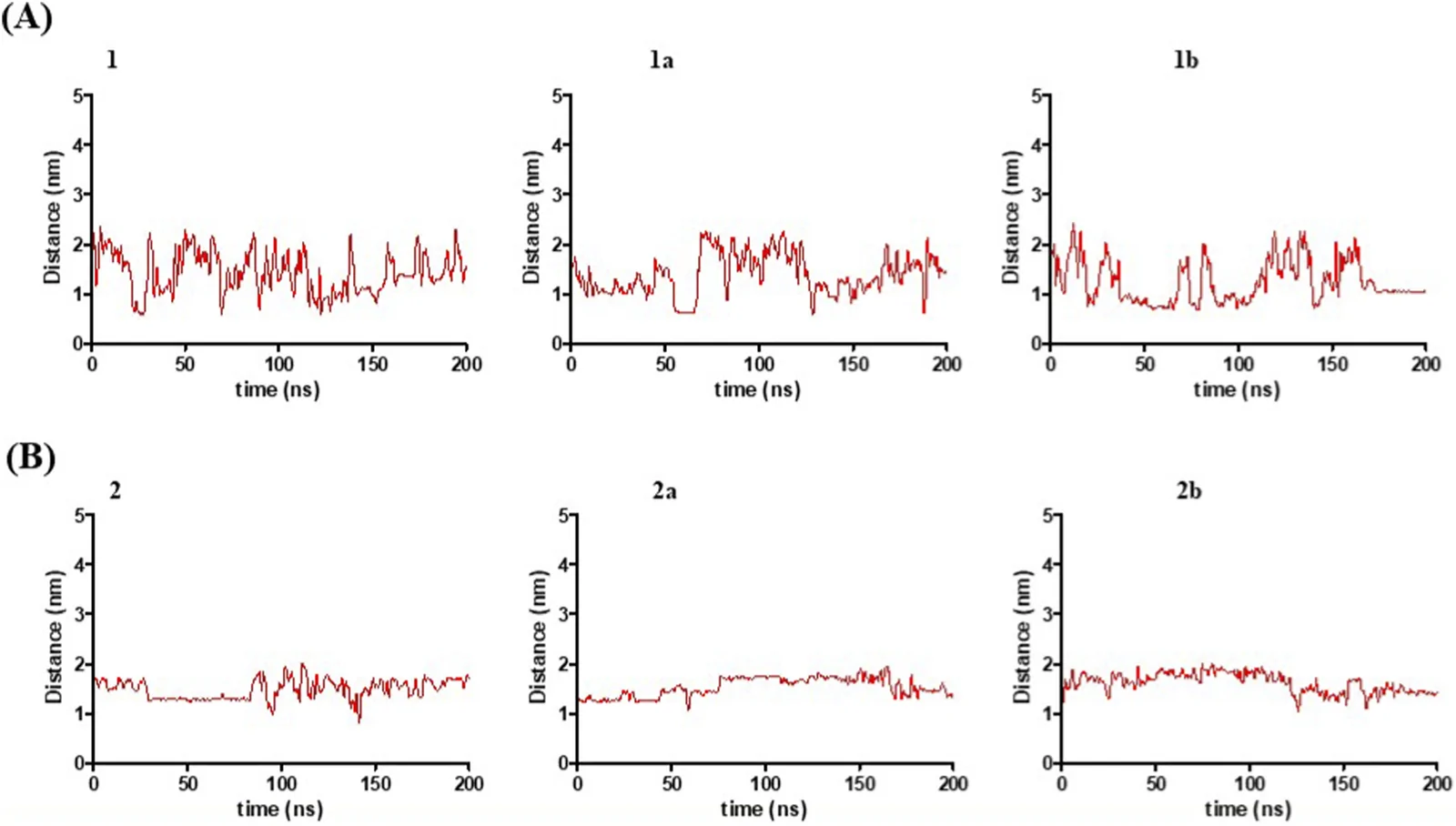
Molecular dynamics stability of parent peptides and novel conjugates. The distance between the first and the last α-carbon (1 ns moving average) during 200 ns molecular dynamics simulations. **(A)** Peptide **1** series: **1**, **1a**, **1b**. **(B)** Peptide **2** series: **2**, **2a**, **2b**.

Figure 10 and Supplementary Table S5 summarize the average distances between the antibiotic anchor atom and the N- and C-terminal α-carbons of the peptide moiety. For norfloxacin conjugates **1a** and **2a**, the anchor atom was the carbon at position 3 of norfloxacin, while for metronidazole conjugates **1b** and **2b** it was the nitrogen at position 1 of metronidazole. In all conjugates, the distance to the C-terminal α-carbon was consistently greater than to the N-terminal α-carbon, indicating a preferential orientation of the antibiotic closer to the N-terminus. Metronidazole-containing conjugates exhibited longer average distances overall compared to norfloxacin conjugates, with **2b** showing the greatest extension at 1.84 ± 0.59 nm to the last α-carbon. Similarly, peptide 2-based conjugates **2a** and **2b** displayed slightly larger distances than their peptide **1** counterparts, **1a** and **1b**. The relatively large standard deviations observed across all systems reflect the conformational flexibility of the conjugates in aqueous solution.

**FIGURE 10:**
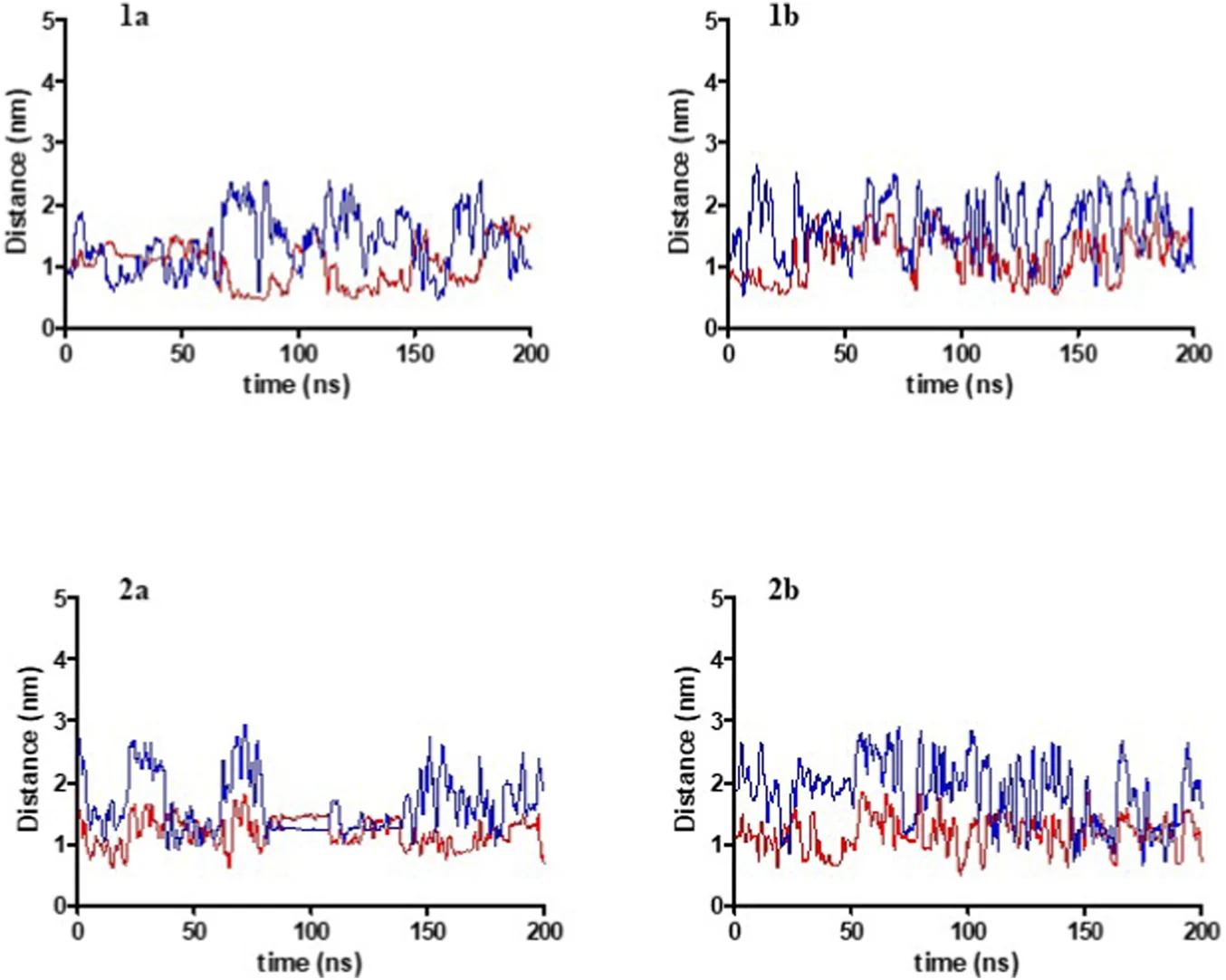
The distance between the carbon atom from position 3 of norfloxacin (**1a** and **2a**) and the nitrogen atom from position 1 of metronidazole (**1b** and **2b**) and the first (in red) and the last (in blue) α carbons from the peptides **1a, 1b/2a, 2b** (1 ns moving average) in the molecular dynamics simulations.

To gain a realistic view on the most preferred conformation of the peptides, the clustering of the poses of the studied peptides during the molecular dynamics simulations was made using 0.3 nm cutoff and the data is presented in Supplementary Table S6. Cluster analysis of the molecular dynamics (MD) trajectory revealed a strongly uneven distribution of conformational populations across the evaluated peptides. In most cases, the conformational ensemble is dominated either by a single highly populated cluster or by a highly fragmented distribution, indicating different degrees of structural stability and flexibility depending on the system.

For peptide **2**, a single dominant cluster accounts for 80.30% of the total poses, indicating a highly stable conformational state with strong preference for one major energy minimum. A secondary cluster contributes only 6.39%, while all remaining clusters are below 5%, confirming a well-defined and highly populated metastable state. Similarly, peptide **2a** shows a less pronounced but still dominant first cluster (27.20%), followed by a rapid decay in cluster populations (≤5% for subsequent clusters). This suggests a semi-stable conformational ensemble with one preferred state, but increased structural heterogeneity compared to system 2. System **2b** shows a more distributed pattern, with the most populated cluster accounting for only 6.04% of the trajectory. The remaining clusters are of comparable low occupancy (1.29%–4.61%), indicating a highly heterogeneous conformational landscape without a clearly dominant state. In contrast, systems **1, 1a,** and **1b** exhibit generally low first-cluster populations (6.69%, 6.88%, and 5.48%, respectively), followed by similarly low secondary cluster contributions. This pattern indicates extensive conformational dispersion and a lack of a dominant structure, consistent with high flexibility and rapid interconversion between multiple local minima. The graphical structures of the conformation of the studied peptides which were found in the most populated cluster are depicted in Figure 11. For the 3D structures of peptides and their conjugates obtained from the molecular dynamics conformations clustering, their electrostatic potential maps were depicted in Figure 12 and some molecular properties were computed and presented in Table 3.

**FIGURE 11:**
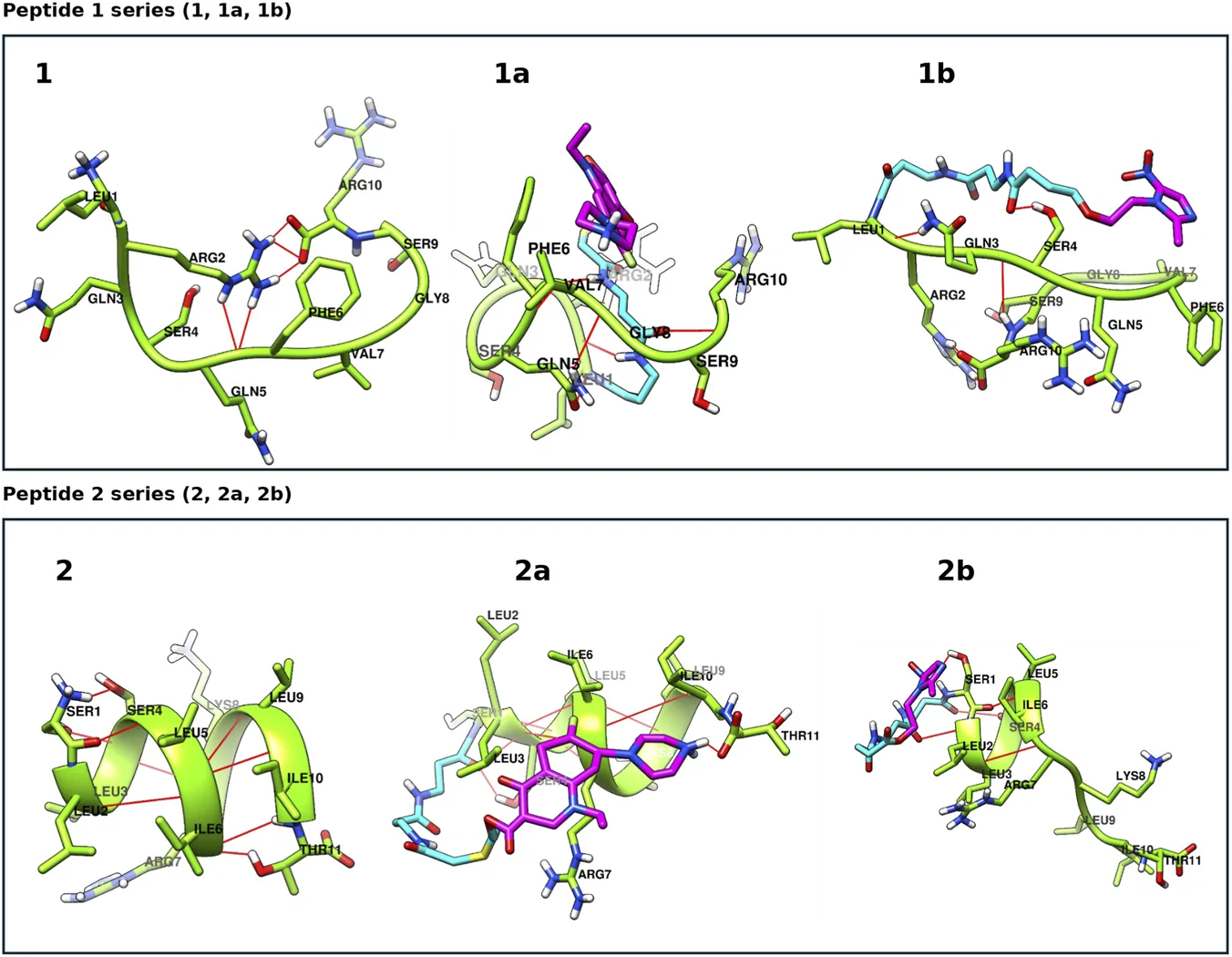
3D structures of peptides **1** and **2** and their conjugates (**1a, 1b, 2a, 2b**) in water, obtained from MD conformational clustering. Metronidazole and norfloxacin: magenta; peptide: green; linker: azure. Red lines: polar intramolecular interactions.

**FIGURE 12:**
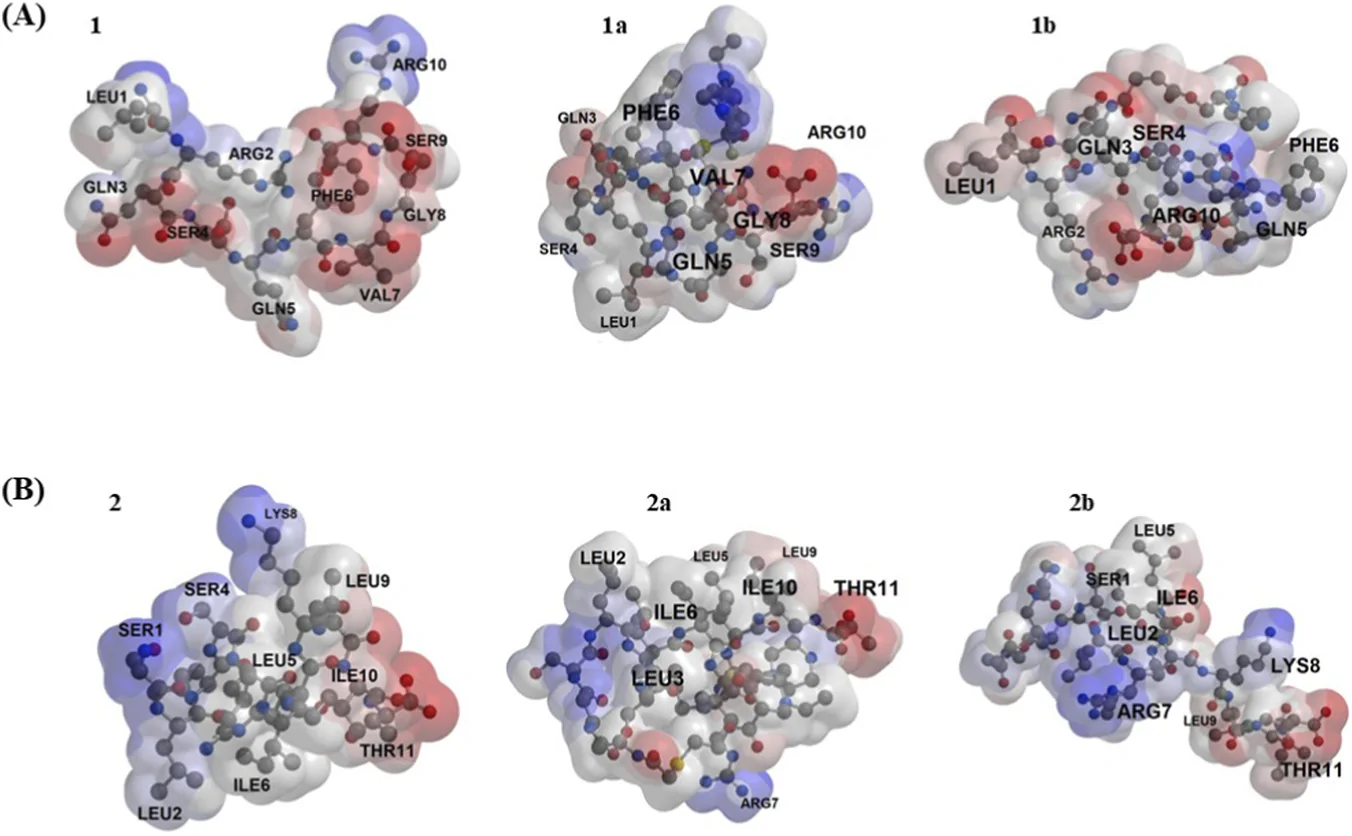
**(A)** Electrostatic potential maps of peptides **1**, **1a, 1b** obtained from the molecular dynamics conformations clustering. **(B)** Electrostatic potential maps of peptides **2**, **2a, 2b** obtained from the molecular dynamics conformations clustering. Hydrogen atoms were removed for clarity.

**TABLE 3:** Molecular descriptors calculated for studied peptides in water.

Peptide	Dipole moment (D)	HOMO		LUMO		Area (Å^2^)	Polar surface area (Å^2^)	Volume (Å^3^)	Polarizability
		eV	Location	eV	Location				
**1**	62.80	−8.17	COOH	1.50	Phe6 sidechain	1264.27	476.896	1159.28	133.14
**1a**	35.81	−7.10	COOH	0.32	Quinolone	1735.55	538.565	1679.39	175.86
**1b**	28.82	−8.30	COOH	0.27	Nitroimidazole	1628.80	568.089	1518.47	162.54
**2**	140.62	−7.56	COOH	1.38	Ser1-Leu3 backbone	1391.46	390.799	1308.72	145.43
**2a**	62.21	−7.65	COOH	0.93	Quinolone	1941.23	481.658	1857.48	190.04
**2b**	65.71	−7.76	COOH	−0.60	Nitroimidazole	1826.58	532.859	1690.72	176.84

Overall, the clustering results reveal two distinct behaviors across the systems: peptides such as **2** and **2a**, which exhibit partially to strongly stabilized conformations, and peptides **1, 1a, 1b,** and **2b**, which display highly dynamic and heterogeneous conformational changes with no single prevailing structural state. These findings suggest that structural stabilization is strongly system-dependent and likely influenced by the specific modifications introduced in each construct.

The electrostatic potential maps computed on the cluster-representative conformations (Figure 12) provide a dynamically weighted view of the surface charge distribution. For peptide **1**, the representative pose retains the electropositive charge centered on Arg2, Arg10 and the terminal nitrogen atom. For conjugates **1a** and **1b**, the norfloxacin and metronidazole moieties occupy peripheral, solvent-facing positions relative to the peptide core, without substantially altering the polarity centered on the arginine residues. For peptide 2 and its conjugates, the cationic area comprised by the sidechains of Arg7 and Lys8 is preserved in the representative conformation, consistent with the high conformational stability of this scaffold (Supplementary Table S6). It should be noted that, for peptides **1**, **1a**, **1b**, and **2b**, the above-displayed cluster-representative structure represents only 5.48%–6.88% of the total trajectory poses (Supplementary Table S6), reflecting the highly heterogeneous conformational landscape of these peptides, whereas for peptides **2** and **2a** it represents 80.30% and 27.20% of poses, respectively. Consequently, the electrostatic surface depicted for the more flexible systems should be interpreted as one representative state among many co-existing conformations rather than a dominant structural feature.

Molecular descriptors calculated on the cluster-representative conformations (Table 3) revealed consistent trends related to molecular size upon conjugation. Covalent attachment of norfloxacin or metronidazole increased the molecular surface area, volume, and polar surface area for all four conjugates relative to their respective parent peptides. For peptide **1**, the area increased from 1264.27 Å^2^ to 1735.55 Å^2^ (**1a**) and 1628.80 Å^2^ (**1b**), while for peptide **2**, the area increased from 1391.46 Å^2^ to 1941.23 Å^2^ (**2a**) and 1826.58 Å^2^ (**2b**). A similar trend was observed for molecular volume and polarizability, which increased from 133.14 (peptide **1**) and 145.43 (peptide **2**) to values between 162.54 and 190.04 for the conjugates, reflecting the larger and more polarizable electron cloud introduced by the antimicrobial moieties.

In contrast to molecular size, the dipole moment decreased upon conjugation for both parent peptides. Peptide **1** displayed a dipole moment of 62.80 D, which decreased to 35.81 D (**1a**) and 28.82 D (**1b**), while peptide **2** showed a more pronounced decrease, from 140.62 D to 62.21 D (**2a**) and 65.71 D (**2b**). This reduction, despite the increased molecular size and polarizability of the conjugates, suggests that the antimicrobial moieties and linker introduce local charge redistribution that partially cancels the overall dipole of the parent peptide, most probably due to intramolecular interactions.

Analysis of the frontier molecular orbitals showed a narrowing of the HOMO–LUMO energy gap for all conjugates relative to their parent peptides. For peptide **1**, the gap decreased from 9.67 eV to 7.42 eV (1a) and 8.57 eV (**1b**), while for peptide **2**, the gap decreased from 8.94 eV to 8.58 eV (**2a**) and 7.16 eV (**2b**). Notably, the HOMO was consistently localized at the C-terminal carboxylic acid group across all six peptides, regardless of conjugation. In contrast, the LUMO location shifted markedly upon conjugation: in the parent peptides, the LUMO was localized within the peptide backbone or side chain (Phe6 side chain for peptide **1**; Ser1–Leu3 backbone for peptide **2**), whereas in all four conjugates, the LUMO shifted entirely onto the antimicrobial moiety, specifically the quinolone ring moiety in the norfloxacin conjugates (**1a**, **2a**) and the nitroimidazole ring in the metronidazole conjugates (**1b**, **2b**). This consistent change indicates that the antibiotic pharmacophores act as the preferential electron-accepting sites within the conjugated structures, directly supporting the reduced HOMO–LUMO gap and suggesting that the antimicrobial moieties, rather than the peptide scaffold itself, govern the electron-accepting character of the conjugates. The most pronounced effect was observed for **2b**, the only system displaying a negative LUMO value (−0.60 eV), localized on the nitroimidazole ring, consistent with the well-established bioreductive activation mechanism of nitroimidazole class of antimicrobials, which relies on enzymatic reduction of the nitro group.

These frontier molecular orbital trends carry direct mechanistic relevance for the antibacterial behavior discussed throughout this study. The consistent localization of the HOMO at the C-terminal carboxylic acid across all six peptides and conjugates reflects the free-acid design common to the entire series and is primarily relevant to the electrostatic/toxicity rationale discussed above, rather than to antibiotic-specific target engagement. In contrast, the localization of the LUMO from the peptide backbone onto the antibiotic pharmacophore upon conjugation is mechanistically meaningful for each drug class individually. For the norfloxacin conjugates (**1a**, **2a**), the LUMO localized on the quinolone ring is consistent with the electron-accepting character required for π-stacking/charge-transfer interactions between the quinolone chromophore and the DNA–gyrase (or DNA–topoisomerase IV) complex, the accepted basis of fluoroquinolone target engagement; the narrowed HOMO–LUMO gap upon conjugation may therefore be interpreted as electronically compatible with, rather than detrimental to, this mechanism once norfloxacin is released. For the metronidazole conjugates (**1b**, **2b**), the LUMO localized on the nitroimidazole ring—and, most notably, the uniquely negative LUMO value obtained for **2b** (−0.60 eV)—directly reflects the electron-accepting capacity of the nitro group that is the prerequisite for its single-electron bioreductive activation into the cytotoxic nitroso-radical intermediate. In this sense, **2b** is, if anything, the most electronically “primed” for activation among the four conjugates; the fact that this electronic predisposition does not translate into potent activity under the aerobic assay conditions used here (Table 2) reinforces, at the electronic-structure level, the interpretation developed above that the redox-dependent activation requirement of metronidazole—not an unfavorable electronic configuration of the conjugate itself—is the principal factor limiting the antibacterial performance of **2b**. Taken together, the HOMO–LUMO analysis indicates that conjugation preserves, and in the case of metronidazole even enhances, the electronic character required for each antibiotic’s canonical mechanism of action, supporting the interpretation that the reduced potency of the metronidazole conjugates originates downstream of drug release and electronic activation-readiness, at the level of environmental redox conditions rather than molecular electronic structure.

## 3 Discussions

The structural modulations implemented in this study for the development of novel antibiotic-conjugated antimicrobial peptides aimed to expand the antimicrobial spectrum of conventional norfloxacin and metronidazole and optimize therapeutic selectivity, thereby ensuring a target-specific enhancement of antimicrobial activity without inducing non-specific toxicity (Scheme 1).

In order to enhance the safety profile for potential therapeutic use, in this study both AMPs sequences of urechistachykinin I and decoralin were synthesized with a C-terminal carboxyl group, since these variants are known to possess negligible neurotoxicity and respectively hemolytic activity when compared to their C-terminal amidated analogues (Konno et al., 2007; Mao et al., 2024; Strandberg et al., 2007). While the C-terminal carboxylate design provides a structural basis for anticipating a favorable safety profile, direct experimental validation of hemolytic activity and mammalian cell cytotoxicity for the specific peptide-drug conjugates reported here (**1a**, **1b**, **2a**, **2b**) was beyond the scope of the present study and is identified as a priority for follow-up work. Such studies should include a standard hemolysis assay on human or sheep erythrocytes and a viability/cytotoxicity assay in relevant mammalian cell lines (e.g., HepG2 for hepatic metabolic relevance, HEK293 for general cytotoxicity screening), ideally reported as a selectivity index relative to the MIC values obtained here (Table 2), in order to rigorously define the therapeutic window of these conjugates prior to any further preclinical development. The development of these peptide-drug conjugates (PDCs) relies on a drug delivery design involving the pathogen-cleavable ester-type linkage between the cationic membrane-active peptide sequences and norfloxacin or metronidazole. This specific design exploits the metabolic differences between the healthy host environment and the active site of bacterial infection. The ester linker acts as a stable bridge during systemic circulation. While intact, the conjugate remains largely inactive or possesses attenuated systemic toxicity. This prodrug approach masks the non-specific toxicity of the small-molecule antibiotic. The positive charge of the cationic peptides guides the entire peptide-drug conjugate toward the negatively charged bacterial membranes through electrostatic interactions, concentrating the therapeutic agent directly at the target site.

In addition to the pathogen-cleavable ester linker, a di-β-alanine motif was selected as a spacer based on well-established literature precedents showing that this linker preserves receptor affinity, as it is electrically neutral, enzymatically stable, and lacks steric hindrance (Effendi et al., 2021; Maria et al., 2024). The non-natural geometry of the β-peptide bond confers robust resistance to proteolytic degradation by serum proteases and endopeptidases, markedly prolonging the plasma half-life of the conjugate and preventing premature cargo leakage during systemic circulation. Furthermore, this spatial extension effectively overcomes the steric hindrance typically observed during the coupling of bulky cationic peptides with bulky, anchored antibiotics such as norfloxacin or metronidazole.

Upon reaching the bacterial microenvironment, the ester linker undergoes site-specific cleavage. Pathogen bacteria naturally secrete a high concentration of hydrolytic enzymes, specifically esterases and lipases. These enzymes recognize and hydrolyze the ester bond within the linker. This pathogen-triggered activation ensures a targeted release of active drugs. Once the ester linker is cleaved, the cationic peptide chain disrupts the bacterial lipid bilayer. This action destabilizes the membrane potential, and increases cell permeability, thus facilitating the rapid influx of the newly released antibiotics into the bacterial cytoplasm. Norfloxacin can then bind to bacterial DNA gyrase and topoisomerase IV to inhibit DNA replication. Concurrently, in anaerobic environments, metronidazole is reduced to its active toxic radical form, disrupting the pathogen’s DNA structure.

By combining membrane permeabilization with intracellular target inhibition, this design significantly lowers the minimal inhibitory concentration, overcomes existing resistance mechanisms, and minimizes off-target toxicity in host tissues.

The differential intrinsic antimicrobial activity of the two parent peptides can be rationalized within the classical two-step model of AMP–membrane interaction, in which an initial electrostatic attraction between the cationic peptide and anionic membrane components (phospholipids, LPS) is followed by hydrophobic insertion into the lipid bilayer, the latter being the step that actually drives permeabilization. Both parent peptides carry an identical net charge of +2 (Arg2/Arg10 in urechistachykinin I; Arg7/Lys8 in decoralin), and would therefore be expected to engage the anionic bacterial surface with comparable initial affinity. However, the two sequences differ in their capacity for the subsequent hydrophobic insertion step: decoralin (SLLSLIRKLIT) contains six hydrophobic residues (Leu×4, Ile×2) arranged to support an amphipathic α-helical conformation, concentrating hydrophobic bulk on one face of the helix and cationic residues on the other—a classical AMP architecture optimized for membrane insertion. Urechistachykinin I (LRQSQFVGSR), by contrast, is comparatively polar (Gln × 2, Ser × 2, Gly × 1) and lacks a comparable amphipathic organization. This structural asymmetry between electrostatic driving force (similar for both peptides) and hydrophobic insertion capacity (markedly favoring decoralin) is consistent with the complete lack of activity observed for peptide **1** and the broad-spectrum activity of peptide **2** (Table 2), and supports the interpretation that electrostatic attraction alone, without sufficient hydrophobic/amphipathic character, is insufficient to drive productive membrane disruption. Conjugation with norfloxacin or metronidazole did not eliminate the cationic surface domains responsible for the initial electrostatic step, as the electrostatic potential maps of the representative MD conformations show the Arg/Lys side chains remaining solvent-exposed and largely unperturbed by the antibiotic moiety (Figure 12), while the antibiotic itself occupies a peripheral, non-competing position. The reduction in overall dipole moment upon conjugation (Table 3) likely reflects local charge redistribution around the linker rather than loss of the membrane-relevant cationic domains themselves. Taken together, these aqueous-phase structural observations are consistent with preservation of the electrostatic targeting function of both scaffolds upon conjugation, while the hydrophobic/amphipathic character—inherent to decoralin but largely absent in urechistachykinin I—remains the limiting factor governing productive membrane insertion and, consequently, overall antimicrobial potency. Direct confirmation would require membrane-embedded simulations, which are currently underway. As a preliminary *in silico* indicator of solubility-relevant lipophilicity, calculated octanol-water partition coefficients (cLogP) were obtained for the parent peptides and their conjugates using ChemDraw Professional, version 22.2.0 (Table 4). Both parent peptides are predicted to be strongly hydrophilic (cLogP = −10.32 for peptide **1**; −1.26 for peptide **2**), consistent with the amino acid composition-based hydrophobicity analysis presented above. Conjugation with either antibiotic modestly increased calculated lipophilicity in all four cases (ΔcLogP = +0.38, +0.07, +0.54, and +0.22 for **1a**, **1b**, **2a**, and **2b**, respectively), with the norfloxacin conjugates showing a larger shift than their metronidazole counterparts, consistent with the more extended aromatic quinolone system of norfloxacin relative to the smaller, more polar nitroimidazole ring of metronidazole. Notably, all four conjugates remain net hydrophilic (cLogP < −0.7), suggesting that aqueous solubility is likely preserved despite antibiotic conjugation. As an experimentally grounded check on this computational trend, the rank order of experimental RP-HPLC retention times obtained for the four conjugates matched exactly the rank order of calculated cLogP (**1b** < **1a** < **2b** < **2a** for both parameters), providing chromatography-based support for the computational lipophilicity ranking.

**TABLE 4:** Calculated octanol-water partition coefficients (cLogP) of the parent peptides **1,2** and their peptide-antibiotic conjugates **1a,b** and **2a,b**. The RP-HPLC retention times were obtained using Zorbax SB-C18 column (4.6 × 100 mm, 3.5 micron), maintained at 40 °C, mobile phase: 0.1% (v/v) formic acid in water (Solvent A) and 0.1% (v/v) formic acid in acetonitrile (Solvent B), 1 mL/min flow rate in linear gradient from 2% to 95% B (0–15 min).

Compound	Calculated cLogP	ΔcLogP vs. parent peptide	Parent peptide	Conjugated antibiotic	RP-HPLC Retention time (min)
Peptide 1	−10.32	—	—	—	—
Peptide 2	−1.26	—	—	—	—
Conjugate **1a**	−9.94	+0.38	Peptide **1**	Norfloxacin	7.1
Conjugate **1b**	−10.25	+0.07	Peptide **1**	Metronidazole	5.4
Conjugate **2a**	−0.72	+0.54	Peptide **2**	Norfloxacin	8.1
Conjugate **2b**	−1.04	+0.22	Peptide **2**	Metronidazole	7.2

The antimicrobial profiles of the tested peptide-antibiotic conjugates (Table 2) are strongly dependent on the nature of the conjugated antibiotic and the peptide scaffold. The covalent conjugation of norfloxacin to the urechistachykinin I sequence (yielding conjugate **1a**) significantly enhanced antibacterial efficacy against *E. coli* ATCC 8739 and *S. aureus* ATCC 29213, while also improving antifungal potency against the yeast pathogen *Candida parapsilosis*.

Figure 1 quantifies the effect of antibiotic conjugation relative to the unmodified parent peptide **2** across the full panel of tested strains. Conjugation with norfloxacin (**2a**) increased potency relative to peptide **2** in 9 of 13 strains (median 2.3-fold), with the largest gains observed against *C. parapsilosis* ATCC 22019 and *S. enterica* ATCC BAA-2162 (14-fold each) and *L. monocytogenes* DSMZ 115292 (7.0-fold). Notably, the four strains for which **2a** underperformed relative to peptide **2** (*B. subtilis* ATCC 11774, *S. aureus* ATCC 29213, *C. albicans* ATCC 1386, *S. pyogenes* ATCC 12344; all 0.6-fold) were precisely those against which the unconjugated peptide **2** already displayed some of its lowest MIC values (Table 2), suggesting that conjugation trades a modest loss of potency where the parent peptide’s intrinsic membrane-disruptive activity is already near-maximal for a broadened, more consistent spectrum across strains where peptide **2** alone is weak or inactive. Conjugation with metronidazole (**2b**) showed a more mixed pattern (median 0.7-fold, indicating a modest net reduction in potency relative to peptide **2**), but with clear, strain-specific gains against *E. faecalis* ATCC 29212 (2.6-fold), *L. monocytogenes* DSMZ 115292 (2.5-fold), and, to a lesser extent, *S. enterica* ATCC BAA-2162 and *A. terreus* DSMZ 23081 (1.3-fold each), which is consistent with the improved potency of **2b** against facultative anaerobic strains. Taken together, these fold-change data support the interpretation that the antibiotic-conjugation strategy employed here does not uniformly enhance potency across all targets, but rather reshapes the antimicrobial spectrum of peptide **2** in a target-dependent manner, consistent with the design objectives (broadened aerobic/facultative coverage for **2a**, targeted facultative-anaerobe enhancement for **2b**) discussed throughout this section. Conjugation of norfloxacin to the decoralin peptide sequence, conducting to **2a**, broadened the antimicrobial spectrum to encompass both yeast and fungal pathogens. Although this peptide-drug conjugate did not surpass the antibacterial potency of the free antibiotic, it exhibited enhanced bacterial inhibition compared to the unmodified decoralin (peptide **2**) against several Gram-negative (*E. coli* ATCC 8739 and ATCC 25922, *S. enterica* ATCC BAA-2162 and ATCC 14028) and Gram-positive (*E. faecalis* ATCC 29212, *S. epidermidis* ATCC 12228, and *L. monocytogenes* DSMZ 115292) strains. These findings demonstrate that the reduced antibacterial activity inherent to decoralin lacking the C-terminal amide moiety (-CONH_2_) was successfully compensated *via* strategic N-terminal structural modulations. Specifically, the carbon-nitrogen backbone of the two β-alanine residues provides an extended and flexible spacer overcoming steric hindrance and preventing rapid degradation by peptidases, while covalent linkage to norfloxacin *via* ester bond enhances the antimicrobial efficacy and targeted delivery. The lower upfront activity of the conjugate relative to the free norfloxacin is likely due to the controlled, time-dependent cleavage of the ester bond, which limits the immediate bioavailability of norfloxacin in the assay medium. This is consistent with the molecular dynamics results: **1a** and **2a** possess high solvent accessible surface area (∼19.5 nm^2^) (Supplementary Table S1), with **2a** exhibiting one of the strongest peptide-water Coulombic interactions (−1661.95 kJ/mol) (Supplementary Table S2). This enhanced hydration and surface exposure may facilitate bacterial membrane interaction prior to ester cleavage and drug release.

In contrast, metronidazole conjugates **1b** and **2b** showed limited or abolished activity. Conjugate **1b** remained inactive, similar to parent peptide **1**, while **2b** generally lost the potent activity of peptide **2** (Table 2). Peptide **2** displayed broad-spectrum activity against both Gram-positive and Gram-negative bacteria (Table 2), consistent with a peptide-mediated membrane-disruptive mechanism. Conjugation with metronidazole to give **2b** retained activity on facultative anaerobe strains (Table 2). Specifically, **2b** displayed potent antibacterial activity against *L. monocytogenes* DSMZ 115292 (MIC = 11.86 μM), *E. faecalis* ATCC 29212 (MIC = 23.12 μM), *S. enterica* ATCC BAA-2162 (MIC = 92.50 μM) and *B. subtilis* (MIC = 23.12 μM), alongside improved antifungal potency against *Aspergillus terreus* DSMZ 23081 (MIC = 92.50 μM). However, **2b** was less potent and had a narrower spectrum than parent peptide **2**, indicating that conjugation partially compromised the intrinsic antimicrobial efficacy of the peptide scaffold. Metronidazole is known to be selectively active against anaerobes due to redox-dependent activation. Although no strict anaerobes were tested here, the improved activity of **2b** on facultative strains suggests that peptide-mediated delivery may partially overcome the activation limitation of the drug. This potency enhancement can be rationalized by the combined contribution of decoralin’s broad-spectrum membrane activity and the metronidazole payload. These findings support further investigation of pathogen-cleavable peptide-metronidazole conjugates, but optimization of linker design and drug release is needed to achieve a true dual-mechanism effect.

The molecular dynamics data indicate that the metronidazole conjugates are more conformationally flexible than the parent peptides and the norfloxacin analogues, with higher RMSD up to 0.59 ± 0.14 nm, larger Radius of gyration, and low cluster populations <6.1% (Supplementary Table S1 and S6). Additionally, the LUMO is localized on the nitroimidazole moiety (Table 3), but the overall flexibility and extended drug-to-peptide distances up to 1.84 ± 0.59 nm (Supplementary Table S5) may prevent the conjugate from efficiently interacting with its target or entering the cell before linker cleavage.

Collectively, molecular dynamics results correlated with antimicrobial activity indicate that the di-β-alanine combined with ester linker can successfully deliver norfloxacin while preserving activity, but conjugation to metronidazole compromises potency, likely due to increased conformational flexibility and unfavorable electronic or steric effects. Linker design and drug identity are therefore critical for maintaining the bioactivity of peptide-antibiotic conjugates.

The reliability of molecular dynamics simulations in predicting biologically relevant conformational states is well established when the technique is discussed alongside high-resolution experimental structural methods. NMR spectroscopy—both in solution/membrane-mimetic settings and, increasingly, in living cells—is widely regarded as a gold-standard complementary approach for defining the functional conformational states of biomolecules in complex environments, providing atomic-resolution structural insight that parallels and can validate computational predictions (Eladl et al., 2025a; Eladl, 2025b). In the specific context of antimicrobial peptides, recent reviews emphasize that atomistic and coarse-grained MD simulations of peptide-membrane interactions are most robust when informed by experimentally derived structural parameters from NMR and related biophysical techniques, underscoring the value of integrating computational and experimental approaches to translate simulation-derived mechanistic models into reliable predictions of in-cell or *in vivo* behavior (Cresca et al., 2026; Jayawardena et al., 2026). While the present study did not include direct experimental structural validation (solid-state NMR) of the modeled conjugate conformations, the MD protocol employed here follows simulation parameters and force fields established in the broader AMP-membrane simulation literature, lending support to the structural trends reported (conformational flexibility, SASA, electrostatic surface distribution) as a reasonable proxy for in-cell behavior; direct experimental structural validation remains, nonetheless, an important direction for future work.

A closer comparison between the norfloxacin- and metronidazole-conjugates suggests that the divergent outcome is unlikely to stem from differential linker cleavage efficiency, since both antibiotics were attached through the same pathogen-cleavable ester chemistry and are expected to be released upon esterase/lipase action at a similar rate. Rather, three complementary factors appear to account for the reduced potency of **2b**. First, and most fundamentally, metronidazole’s antimicrobial mechanism intrinsically depends on bioreductive activation of its nitro group under low-redox-potential, anaerobic or microaerophilic conditions; the aerobic conditions used in the present MIC assays are therefore inherently unfavorable for generating the cytotoxic nitroso-radical intermediate, regardless of how efficiently the conjugate is cleaved or how much free metronidazole is released. This contrasts with norfloxacin, whose inhibition of DNA gyrase/topoisomerase IV is redox-independent and equally effective under the aerobic assay conditions, which plausibly explains the consistently better performance of the norfloxacin conjugates (**1a**, **2a**) relative to the metronidazole conjugates (**1b**, **2b**). Second, unfavorable “pharmacokinetic-like” behavior at the molecular level may further limit **2b**’s activity: MD simulations show that the metronidazole conjugates adopt more heterogeneous, extended conformations (higher RMSD, larger radius of gyration, low cluster populations <6.1%; see Supplementary Tables S1 and S6) and a longer average drug-to-peptide distance (up to 1.84 ± 0.59 nm for **2b**; Supplementary Table S5) than the norfloxacin conjugates. This extended, flexible architecture may reduce the efficiency with which the peptide moiety can engage the bacterial membrane and position the antibiotic payload for release and uptake, effectively lowering local bioavailability at the target site even before any redox limitation comes into play. Third, the LUMO of **2b** is uniquely negative (−0.60 eV) and localized on the nitroimidazole ring (Table 3), consistent with its predisposition toward enzymatic reduction; however, this same electronic feature also implies that, absent an anaerobic microenvironment, the moiety may instead participate in non-productive electronic interactions rather than generating the active radical species.

This electronic picture also clarifies why the norfloxacin conjugates do not suffer the same limitation: the LUMO of **1a** and **2a** is instead localized on the quinolone ring, consistent with the electron-accepting, charge-transfer character required for the fluoroquinolone pharmacophore to engage the DNA–gyrase/topoisomerase IV complex—a redox-independent mechanism that remains fully functional under the aerobic assay conditions used here. Viewed together, the HOMO–LUMO analysis indicates that conjugation preserves, and in the case of **2b** even enhances, the electron-accepting character required for each antibiotic’s mechanism. Conjugate **2b** is the most electronically “primed” for bioreductive activation among the four conjugates, which reinforces, at the electronic-structure level, the conclusion that its reduced potency originates from the redox-dependence of the local environment rather than from an electronically unfavorable conjugate structure.

Taken together, we interpret the reduced potency of **2b** as being driven predominantly by the intrinsic redox-dependence of metronidazole’s mechanism of action under the aerobic assay conditions, with conformational and electronic factors acting as secondary, compounding contributors - rather than by inefficient linker chemistry, which is shared with the more potent norfloxacin conjugates. Antifungal activity evaluation of peptides versus peptide-drug conjugates indicates that chemical conjugation *via* a pathogen-cleavable ester linker enhances potency against specific fungal pathogens, although it does not confer a broad-spectrum antimicrobial profile comparable to conventional reference antibiotics. Nevertheless, the covalent conjugation of the decoralin N-terminus with norfloxacin *via* an ester-type linkage yielded promising hybrid agents that operate through a dual-action mechanism, thereby successfully broadening the therapeutic spectrum of this traditional antibiotic. Consequently, further investigations encompassing an expanded panel of fungal strains are highly warranted to fully elucidate the therapeutic potential of these peptide-drug conjugates.

It should be noted that the proposed dual mechanism of action—membrane permeabilization by the peptide scaffold followed by intracellular inhibition of the released antibiotic—is, at this stage, supported by indirect and computational evidence rather than by direct functional confirmation. The comparative MIC profile of each conjugate relative to its parent peptide and to the free antibiotic (Table 2), together with the MD-derived observation that the norfloxacin and metronidazole moieties occupy peripheral, solvent-exposed positions without disrupting the cationic character of the key arginine/lysine residues responsible for membrane interaction (Figure 12), are consistent with retained membrane-active behavior of the peptide portion after conjugation. However, no assay was performed in this study to isolate and independently confirm each mechanistic component. Future work should therefore include a membrane-permeabilization assay (e.g., SYTOX Green or propidium iodide uptake, or DiSC3(5)-based membrane depolarization) to directly demonstrate peptide-mediated membrane disruption by the conjugates relative to the parent peptides, alongside a target-specific assay for the released antibiotic—such as a DNA gyrase/topoisomerase IV supercoiling inhibition assay for the norfloxacin conjugates and a nitroreductase-dependent activation or anaerobic time-kill assay for the metronidazole conjugates. Such experiments would allow the relative contribution of each mechanistic component to be deconvoluted and would provide direct functional validation of the dual-mechanism hypothesis proposed here. Direct biophysical confirmation of membrane-disruptive activity has been achieved for other rationally designed AMPs. For example, a recent LL-37-derived peptide targeting multidrug-resistant *E. coli* and ESKAPE pathogens was shown, using circular dichroism, to undergo a random-coil-to-α-helix transition specifically upon interaction with bacterial-mimetic (POPE/POPG) vesicles, while surface plasmon resonance and isothermal titration calorimetry demonstrated markedly higher-affinity, more favorable binding to bacterial-mimetic than mammalian-mimetic membranes, and calcein dye-leakage assays confirmed extensive, selective permeabilization of bacterial-like vesicles (Eladl, 2025c). Applying an analogous biophysical characterization strategy, such as conformational analysis upon membrane binding, selective lipid-binding assays, and dye-leakage measurements to the present peptide-drug conjugates would provide the direct, quantitative evidence of membrane-disruptive capacity that is currently inferred only indirectly from MIC comparisons and molecular dynamics simulations, and represents a valuable methodological benchmark for future mechanistic validation of this conjugate class. Given the additional clinical relevance of biofilm-associated persistence among MDR pathogens, we further identify mature-biofilm disruption/inhibition assays as an important complementary direction, since the antimicrobial activity reported here was assessed exclusively against planktonic growth.

Taken together, these findings reveal that the conjugation of norfloxacin and metronidazole to decoralin (peptide **2**) via a pathogen-cleavable ester linkage effectively expands their antimicrobial spectrum to encompass fungal pathogens. Overall, the implemented structural modifications did not induce a non-specific increase of antibacterial efficacy; rather, they produced an enhanced, targeted potency against specific bacterial and fungal strains.

To better contextualize the clinical relevance of these findings, a direct fold-change comparison between the conjugates and the reference antibiotics used as positive controls (Table 2) is warranted. Against facultative anaerobic Gram-positive strains, conjugate **2b** displayed up to a ∼150-fold lower MIC than free metronidazole (e.g., 11.9 μM vs. >1830 μM for *L. monocytogenes* DSMZ 115292), representing a substantial potency advantage over the parent drug and underscoring the clinical potential of this conjugate for anaerobic and facultative anaerobic infections poorly addressed by metronidazole alone. In contrast, the norfloxacin conjugates **1a** and **2a** generally did not surpass the potency of free norfloxacin (e.g., **2a** vs. norfloxacin against *E. coli* ATCC 8739: 8.6 μM vs. 0.9 μM), and none of the tested conjugates exceeded the antifungal potency of fluconazole (e.g., **2a** vs. fluconazole against *C. parapsilosis*: 16.7 μM vs. 0.6 μM). These comparisons indicate that the principal clinical value of the norfloxacin- and antifungal-active conjugates lies not in surpassing the potency of the free reference drugs, but in conferring meaningful antimicrobial/antifungal activity onto peptide scaffolds (**1**, **2**) that are otherwise inactive or only weakly active on their own, together with the potential pharmacokinetic and safety advantages of pathogen-targeted, prodrug-style delivery discussed above.

Figure 2 summarizes this comparison across all strain/antibiotic pairs for which both MIC values could be determined. Conjugate **2b** consistently outperformed free metronidazole against the facultative anaerobic/aerobic strains on which both were active, with potency gains ranging from 39.5-fold (*S. pyogenes* ATCC 12344) to 158.1-fold (*B. subtilis* ATCC 11774; median 79.0-fold; Figure 2). In contrast, the norfloxacin conjugates showed the opposite trend: **1a** was less potent than free norfloxacin against 10 of 11 comparable strains (median 3.6-fold), and **2a** was less potent against all 10 comparable strains (median 11.1-fold), with the exception of *L. monocytogenes* DSMZ 115292, where **1a** and **2a** approached the potency of free norfloxacin (1.2-fold difference for both). Similarly, none of the antifungal-active conjugates matched the potency of fluconazole (median 27.2-fold less potent). These comparisons indicate that the clinical value of the norfloxacin- and fluconazole-comparable conjugates lies not in exceeding the potency of the parent small-molecule antibiotics, but in conferring spectrum-broadening antimicrobial activity onto peptide scaffolds that are otherwise inactive (peptide **1**) or only weakly active (peptide **2**) on their own (see also Table 2), together with the potential targeting and toxicity-mitigation advantages of the pathogen-cleavable prodrug design discussed above. Conjugate **2b**, however, represents a case where covalent linkage to a membrane-active AMP scaffold provides a substantial potency advantage over the free reference drug itself, highlighting its particular promise for further development against facultative anaerobic pathogens poorly controlled by metronidazole alone. In conclusion, the antimicrobial screening revealed two novel norfloxacin-conjugated AMPs (**1a**, **2a**) and one metronidazole-conjugated AMP (**2b**) with enhanced potency against clinically relevant bacterial and fungal pathogens, through a proposed dual mechanism. By retaining the native, unamidated C-terminal carboxylate group (-COOH), the designed PDCs **1a**, **2a** and **2b** are expected to preserve a highly selective therapeutic index, minimizing non-specific hemolytic toxicity or neurotoxicity, based on structural precedent from the parent AMP scaffolds (Konno et al., 2007). Direct experimental confirmation of hemolytic activity and mammalian cell cytotoxicity for these specific conjugates remains to be established.

Beyond the specific mechanisms of norfloxacin and metronidazole discussed above, bacterial susceptibility and resistance to antimicrobial agents are increasingly understood to be modulated at the epitranscriptomic level. Epitranscriptomic regulation of gene expression has emerged as a broadly relevant framework across human disease contexts, encompassing cooperative networks of RNA modifications (Eladl et al., 2026) and context-specific long non-coding RNA dynamics (Eladl and Estfanous, 2026), with growing recognition that analogous regulatory layers operate in bacterial systems. In bacteria specifically, tRNA-modifying enzymes have been shown to regulate stress-adaptive translation reprogramming under antibiotic and oxidative stress, with several modifications enhancing translation of resistance factors and efflux pump components (Fruchard et al., 2025). A concrete example of this interplay is provided by Erm-family methyltransferases, which install an epitranscriptomic m6A mark on the bacterial 23S rRNA to confer cross-resistance to macrolide-class antibiotics, in species such as *S. aureus* included in the present MIC panel (Shields et al., 2024). Similarly, RNA modification networks have been implicated in shaping innate immune responses and cellular adaptation during *P*. *aeruginosa* respiratory infection, another species represented in our panel (Ivanova et al., 2026). While the present study does not directly probe epitranscriptomic regulation, these findings suggest that the bacterial RNA-modification landscape represents a complementary layer of resistance modulation that could interact with or be exploited alongside pathogen-cleavable peptide-antibiotic conjugate strategies such as the one described here, and represents a promising direction for future mechanistic and combination-therapy studies.

## 4 Materials and methods

### 4.1 General methods

#### 4.1.1 Synthesis of antibiotic derivatives

All commercially available reagents necessary for synthesis of target compounds and intermediates were used without further purification. Succinic anhydride and di-tert-butyl dicarbonate (Boc_2_O) were purchased from Alfa Aesar (Thermofisher GmbH, Kandel, Germany). Organic solvents (tetrahydrofuran, anhydrous dichloromethane) were from VWR Chemicals (Vienna, Austria). Acetonitrile (HPLC gradient grade) was from Honeywell (Seetze, Germany).

Norfloxacin and metronidazole were derivatized to *N*-tert-butoxycarbonyl-norfloxacin (compound **a**) and respectively metronidazole monosuccinate ester (compound **b**) based on methods previously reported in the literature (Chen et al., 2023; Mura et al., 2011). The details of the experimental procedures and the obtained yields are provided in Supplementary Material.

Analytical thin-layer chromatography (TLC) was performed on pre-coated silica gel F-254 plates. Chromatography purifications of compounds were performed by column chromatography using silica gel (Kieselgel 60, particle size 0.063–0.200 mm, 70–230 mesh ASTM) purchased from Merck (Darmstadt, Germany).

The synthesis reactions were monitored by RP-HPLC analysis carried out on a Shimadzu Nexera X3 HPLC system (Shimadzu Corporation, Kyoto, Japan) equipped with separation module with two LC-40D pumps, a degassing unit DGU-405, a SIL-40 autosampler, a CTO-40S column oven and a photodiode array detector SPD-M40. The mobile phase was a mixture of 50% acetonitrile and 50% water, in isocratic mode. For metronidazole hemisuccinate monoester, HPLC analysis was performed on Phenomenex Luna C18 analytical column (5 μm, 100 Å) 150 × 4.6 mm i.d., maintained at 40 °C. The flow rate was set up at 0.4 mL/min and UV detection was made at 214 and 317 nm. *N*-tert-butoxycarbonyl-norfloxacin was analysed on Phenomenex Gemini C18 column (3 μm, 110 Å) 50 × 3.00 mm i.d., maintained at 40 °C. The flow rate was set up at 0.2 mL/min and UV detection was made at 214 and 279 nm.

#### 4.1.2 Peptide synthesis

Organic solvents and reagents necessary for solid phase peptide synthesis and purification (N,N-dimethylformamide for peptide synthesis, dichloromethane for peptide synthesis, trifluoroacetic acid, acetonitrile HPLC grade, ethyl acetate, *N,N*-diisopropylethylamine, pyridine, piperidine) were purchased from VWR Chemicals (Vienna, Austria). Fluorenylmethyloxycarbonyl (Fmoc)-protected L-amino acids Fmoc-L-Ser(tBu)-OH, Fmoc-Gly-OH, Fmoc-L-Val-OH, Fmoc-L-Phe-OH, Fmoc-L-Gln(Trt)-OH, Fmoc-L-Arg(Pbf)-OH, Fmoc-L-Leu-OH, Fmoc-β-Ala-OH, Fmoc-L-Lys(Boc)-OH, Fmoc-L-Ile-OH necessary for solid phase peptide synthesis were products of Novabiochem, Merck KGaA (Darmstadt, Germany). Ethyl(hydroxyimino)cyanoacetate (OxymaPure) and *N,N′*-diisopropylcarbodiimide (DIC) were purchased from Carl Roth GmbH (Germany). O-(Benzotriazol-1-yl)-*N,N,N′,N′*-tetramethyluronium hexafluorophosphate (HBTU) was purchased from Thermo Scientific Chemicals (Germany). 2-Chlorotrityl chloride (2-CTC) resin 1.46 mmol/g, 100–200 mesh was purchased from IRIS Biotech GmbH (Marktredwitz, Germany). All commercially available reagents were used without further purification. Polypropylene reservoirs (15 mL), polyethylene frits and polytetrafluoroethylene stopcocks necessary for solid phase peptide synthesis were purchased from Macherey-Nagel GmbH (Düren, Germany).

Purification of peptides and peptide-drug conjugates was performed by RP-HPLC on a Shimadzu Nexera X3 HPLC system with a Phenomenex Luna semi-preparative C18 column (5 μm, 100 Å, 150 × 10 mm), maintained at 40 °C, using as mobile phases: A: 0.1% formic acid in water; B: acetonitrile with 0.1% formic acid. The flow rate was set up at 3 mL/min and UV detection was made at 214, 279 and 317 nm. The following gradient separation procedure was applied: 2%–90% of B in A from 0 to 22 min.

Reversed-phase high performance liquid chromatography (RP-HPLC) analyses of the obtained peptides and peptide-drug conjugates were conducted on a Shimadzu Nexera X3 HPLC system (Shimadzu Corporation, Kyoto, Japan), using Zorbax SB-C18 column (4.6 × 100 mm, 3.5 micron, Agilent Technologies, Santa Clara, CA, United States), maintained at 40 °C, with UV detection at 214 and 279 nm (for norfloxacin-conjugated peptides **1a** and **2a**) and respectively 214 and 317 nm (for metronidazole-conjugated peptides **1b** and **2b**), using the software LabSolutions v5.71 SP2 (Shimadzu, Kyoto, Japan). The mobile phase consisted of 0.1% (v/v) formic acid in water (Solvent A) and 0.1% (v/v) formic acid in acetonitrile (Solvent B). The flow rate was set at 1 mL/min. A gradient elution was applied as follows: 0–15 min, linear gradient from 2% to 95% B; followed by a re-equilibration step with 2% B for 3 min.

The LC-HRMS analysis was performed using a Shimadzu Nexera X3 HPLC system (Shimadzu Corporation, Kyoto, Japan) coupled to a Shimadzu QTOF 9030 mass spectrometer. The LC configuration was equipped with two LC-40D X3 pumps with an integrated degasser, a SIL-40C X3 autosampler, and a CTO-40S column oven. Sample preparation involved a Sigma 204 Centrifuge (Osterode am Harz, Germany), an Analytical Plus Balance (Mettler-Toledo, Switzerland), and an Elma Transsonic 700/H ultrasonic bath (Singen, Germany). Chromatographic separation was achieved on a Phenomenex Gemini column (50 mm × 2.1 mm i.d., 3 µm particle size; Phenomenex, SUA) maintained at 40 °C. The mobile phase consisted of 0.1% (v/v) acetic acid in water (Solvent A) and acetonitrile (Solvent B). A gradient elution was applied as follows: 0–6 min, linear gradient from 10% to 90% B; followed by a re-equilibration step with 10% B for 2 min. The flow rate was set at 0.6 mL/min, and the injection volume was 1 µL. Mass spectrometry detection was performed in positive ionization mode using high resolution scan mode–for accurate mass measurement of parent ions. The ESI source parameters were set as follows: source voltage 3000 V; interface temperature 350 °C, nebulizing gas flow 3 L/min, heating gas flow 12 L/min (air); drying gas flow 12 L/min (nitrogen), desolvation line temperature 250 °C, heat block temperature 400 °C. The time of flight (TOF) tube was daily calibrated using standard solution of sodium iodide 400 mg/L. Data acquisition and data processing were made using LabSolutions 5.135 software (Shimadzu Corporation, Kyoto, Japan).

### 4.2 General procedure for solid-phase peptide synthesis (SPPS)

All reactions were carried out in polypropylene reservoirs (15 mL) fitted with polyethylene frits and polytetrafluoroethylene stopcocks, at room temperature. 2-Chlorotrityl chloride resin was employed to yield a C-terminal carboxyl group.

#### 4.2.1 Loading of first amino acid

2-Chlorotrityl chloride resin (400 mg, 1.46 mmol/g) was swelled in DCM (5.0 mL) for 30 min. DCM was removed then a solution of Fmoc-protected amino acid (1.2 equiv.) and DIPEA (2.5 equiv.) in DCM (3 mL) was added. The reaction mixture was gently shaken at 300 rpm. After 1 h, an additional portion of DIPEA (2.5 equiv.) was added to the resin. After 2 h of gently shaking at 300 rpm, methanol (800 µL) was added to the reaction mixture under continuous shaking for 15 min, for capping the unreacted sites of resin. Then, the resin was washed sequentially with N,N-dimethylformamide (DMF, 3 × 3 mL), dichloromethane (3 × 3 mL), then methanol/dichloromethane (1:1 v/v, 3 × 3 mL), and finally methanol (3 × 3 mL).

Fmoc deprotection of N-terminal amino acid unit was carried out by treatment with a solution of piperidine/DMF (80:20 *v:v*), in two cycles (2 × 3 mL) under continuous shaking at 300 rpm: first deprotection cycle (5 min) followed by washing with DMF (3 mL), followed by second deprotection cycle (15 min). The resin was washed five times with DMF (5 × 3 mL).

Peptide chain elongation was achieved using the following conditions: each Fmoc-protected amino acid (3 equiv.) was activated prior to coupling by treatment with N,N′-diisopropylcarbodiimide (DIC, 3 equiv.) and ethyl 2-cyano-2-(hydroxyimino)acetate (Oxyma Pure, 3 equiv.) in DMF (3 mL) under continuous shaking at 600 rpm for 10 min. The solution was added to the resin and the coupling reactions were carried out under gentle shaking at 300 rpm, between 60 min and 240 min. After each coupling step, the resin was washed five times with DMF (5 × 3 mL). The completion of each coupling reaction was confirmed by a negative Kaiser test.

Cleavage from the resin and total deprotection were achieved using the following conditions: after the last Fmoc removal, the resin was rinsed with DCM (5 × 3 mL) and dried under vacuum at room temperature. Cleavage cocktail (TFA/H_2_O/TIS, 95/2.5/2.5 *v/v/v*) was freshly prepared and added to the resin (2 mL/100 mg resin). The mixture was shaken for 3 h and the solution was added dropwise to cold diethyl ether (30 mL per 2 mL of cleavage solution). The resin was treated once again with 2 mL of cleavage cocktail, followed by precipitation in cold diethyl ether. The formed precipitate was separated by centrifugation (3,000 rpm, 5 min). The supernatant was discarded and the solid was washed twice with cold diethyl ether (2 × 10 mL) and then centrifuged again (3,000 rpm, 5 min). The crude product obtained was air-dried at room temperature, dissolved in a minimum amount of water and freeze dried.

Crude peptides were purified by semi-preparative HPLC on a RP-C18 column as mentioned in Section 4.1.2. The purified products were dissolved in 0.05 M aqueous HCl solution, at a concentration of 10 mg/mL. Lyophilization afforded the pure peptides as hydrochloride salts.

### 4.3 Synthesis of norfloxacin-conjugated peptides **1a** and **2a**

After completion of the peptide sequences **1** (LRQSQFVGSR) and **2** (SLLSLIRKLIT) bound to 2-chlorotrityl chloride resin, two Fmoc-β-Ala-OH (3 equiv.) were sequentially coupled to the deprotected N-terminal leucine (for peptide **1**) and respectively serine (for peptide **2**) unit of the resin-bound peptide chains by the same coupling protocol as mentioned in Section 4.2. Peptide chain elongation, using DIC (3 equiv) and Oxyma Pure (3 equiv) in DMF.

After Fmoc removal by the described general protocol (Section 4.2. Fmoc deprotection), the resin-bound peptides were functionalized with chloroacetic acid (5.0 equiv) pre-activated with DIC (5.0 equiv), in a minimum amount of DMF. Prior to addition to the resin-bound peptides, the mixture of chloroacetic acid and DIC was activated by shaking at 600 rpm for 10 min. The activated solution was then added to the resin, and the coupling reaction was allowed to proceed for 1.5 h. After washing the resin five times with DMF, completion of the reaction was confirmed by a negative Kaiser test, indicating successful incorporation of the chloroacetyl group onto the peptide chain.

Subsequently, the chloroacetylated resin-bound peptides suspended in DMF were treated with 2-mercaptoethanol (3.0 equiv.) in the presence of DIPEA (6.0 equiv.) under continuous shaking at 300 rpm, overnight, at room temperature. The resins were washed five times with DMF followed by addition of a solution of N-tert-butoxycarbonyl-norfloxacin (compound **a**, 3.0 equiv.) pre-activated with O-(Benzotriazol-1-yl)-*N,N,N′,N′*-tetramethyluronium hexafluorophosphate (HBTU, 3.0 equiv.), 4-*N,N*-dimethylaminopyridine (DMAP, 1.5 equiv.) and N,N-diisopropylethylamine (DIPEA, 6 equiv.) in N-methylpyrrolidone (NMP). Prior to addition to the peptide, the coupling mixture was activated by shaking at 600 rpm for 15 min. The coupling reactions were allowed to proceed overnight and then the resin was washed three times with NMP (3 × 3 mL) and three times with DCM (3 × 3 mL) and dried under reduced pressure at room temperature. Then, cleavage cocktail freshly prepared (TFA/H_2_O/TIS, 95/2.5/2.5 *v/v/v*) was added to each resin (2 mL/100 mg resin) and the mixtures were shaken for 3 h. The solution was added dropwise to cold diethyl ether (30 mL per 2 mL of cleavage solution). The resin was treated once again with 2 mL of cleavage cocktail, followed by precipitation in cold diethyl ether. The formed precipitates were separated by centrifugation (3,000 rpm, 5 min). The supernatant was discarded and the solid was washed twice with cold diethyl ether (2 × 10 mL) and then centrifuged again (3,000 rpm, 5 min). The crude products obtained were air-dried (4 h) at room temperature, dissolved in a minimum amount of water and freeze dried. The obtained norfloxacin-peptide conjugates were purified by semi-preparative HPLC on a RP-C18 column as mentioned in Section 4.1.2. Pure fractions were combined, lyophilized. The purified products were dissolved in 0.05 M aqueous HCl solution, at a concentration of 10 mg/mL. The obtained solutions were lyophilized, in order to obtain the pure norfloxacin-peptide conjugates **1a** and **2a** as hydrochloride salts.

#### 4.3.1 Norfloxacin-conjugated peptide **1a**

RP-HPLC: *R*_*t*_ = 7.1 min. ESI-HRMS positive ionisation mode m/z: calculated for C_76_H_117_FN_23_O_21_S [M + H]^+^: 1738.8494, found: 1738.8473; calculated for C_76_H_118_FN_23_O_21_S [M+2H]^2+^: 869.9283, found: 869.9296; calculated for C_76_H_119_FN_23_O_21_S [M+3H]^3+^: 580.2880, found: 580.2880.

#### 4.3.2 Norfloxacin-conjugated peptide **2a**

RP-HPLC: *R*_*t*_ = 8.1 min. ESI-HRMS positive ionisation mode m/z: calculated for C_84_H_142_FN_20_O_21_S [M + H]^+^: 1818.0358; found: 1818.0332; calculated for C_84_H_143_FN_20_O_21_S [M+2H]^2+^: 909.5215, found: 909.5246; calculated for C_84_H_144_FN_20_O_21_S [M+3H]^3+^: 606.6834, found: 606.6859.

### 4.4 Synthesis of metronidazole-conjugated peptides **1b** and **2b**

After completion of the peptide sequences **1** (LRQSQFVGSR) and **2** (SLLSLIRKLIT) bound to 2-chlorotrityl chloride resin, two Fmoc-β-Ala-OH (3 equiv.) were sequentially coupled to the deprotected N-terminal leucine (for peptide **1**) and respectively serine (for peptide **2**) unit of the resin-bound peptide chains by the same coupling protocol, as mentioned in Section 4.2. *Peptide chain elongation*, using DIC (3 equiv) and Oxyma Pure (3 equiv) in DMF. The completion of the coupling reaction was confirmed by a negative Kaiser test. Fmoc deprotection was performed by previously described general protocol (Section 4.2. Fmoc deprotection).

The resins were washed five times with DMF followed by addition of a solution of metronidazole succinate monoester (compound **b**, 3.0 equiv.) pre-activated with DIC (3 equiv.) and Oxyma Pure (3 equiv) in DMF for 10 min. The coupling reactions were allowed to proceed overnight under gentle agitation (300 rpm) and then the resin was washed three times with DMF (3 × 3 mL), three times with DCM (3 × 3 mL) and dried under reduced pressure at room temperature. Then, cleavage cocktail freshly prepared (TFA/H_2_O/TIS, 95/2.5/2.5 *v/v/v*) was added to each resin (2 mL per 100 mg resin) and the mixtures were shaken for 2 h. The cleavage solutions were added dropwise to cold diethyl ether (30 mL per 2 mL cleavage cocktail). The resin was treated once again with 2 mL of cleavage cocktail, followed by precipitation in cold diethyl ether. The formed precipitates were separated by centrifugation (3,000 rpm, 5 min). The supernatant was discarded and the solid was washed twice with cold diethyl ether (2 × 10 mL) and then centrifuged again (3,000 rpm, 5 min). The crude products obtained were air-dried (4 h) at room temperature, dissolved in a minimum amount of water and freeze dried. The obtained metronidazole-peptide conjugates were purified by semi-preparative HPLC on a RP-C18 column as mentioned in Section 4.1.2. Pure fractions were combined, lyophilized. The purified products were dissolved in 0.05 M aqueous HCl solution, at a concentration of 10 mg/mL. The obtained solutions were lyophilized, in order to obtain the pure metronidazole-peptide conjugates **1b** and **2b** as hydrochloride salts.

#### 4.4.1 Metronidazole-conjugated peptide **1b**

RP-HPLC: *R*_*t*_ = 5.4 min. ESI-HRMS positive ionisation mode m/z: calculated for C_66_H_106_N_23_O_22_ [M + H]^+^: 1572.7878; found: 1572.7867; calculated for C_66_H_107_N_23_O_22_ [M+2H]^2+^: 786.8975, found: 786.8958; calculated for C_66_H_108_N_23_O_22_ [M+3H]^3+^: 524.9341, found: 524.9309.

#### 4.4.2 Metronidazole-conjugated peptide **2b**

RP-HPLC: *R*_*t*_ = 7.2 min. ESI-HRMS positive ionisation mode m/z: calculated for C_74_H_131_N_20_O_22_ [M + H]^+^: 1651.9742, found: 1651.9771; calculated for C_74_H_132_N_20_O_22_ [M+2H]^2+^: 826.4907, found: 826.4926; calculated for C_74_H_133_N_20_O_22_ [M+3H]^3+^: 551.3296, found: 551.3291.

### 4.5 *In vitro* antimicrobial activity evaluation

#### 4.5.1 Microbial strains

The following bacterial, yeast and fungal strains were tested: *Escherichia coli* ATCC 8739, *Escherichia coli* ATCC 25922, *S. enterica* subsp. *enterica* (serovar Abony ATCC BAA-2162), *S. enterica* subsp. *enterica* (serovar *Typhimurium*) ATCC 14028, *E. faecalis* ATCC 29212*, P. aeruginosa* ATCC 27853*, Bacillus subtilis* ATCC 11774, *S. pyogenes* ATCC 12344, *S. aureus* ATCC 29213, *S. epidermidis* ATCC 12228, *L. monocytogenes* DSMZ 115292, *C. parapsilosis* ATCC 22019, *C. albicans* ATCC 1386, and *A. terreus* DSMZ 23081. Each strain was initially cultured in 10 mL of the appropriate sterile broth medium: Tryptic Soy Broth (TSB) for *E*. *coli*, *S. aureus, P. aeruginosa, B. subtilis, C. parapsilosis*; Nutrient Broth (NB) for *S*. *enterica, E. coli, S. epidermidis;* Brain Heart Infusion (BHI) for *L. monocytogenes, E. faecalis* and universal yeast medium (YM) for *C. albicans*. The fungal strain *A. terreus* was activated on Potato Dextrose Agar (PDA) slants.

The bacterial and fungal cultures were activated by incubating the broth tubes at 37 °C for 24 h. The purity of each inoculum was verified *via* microscopic examination of Gram-stained smears. Following incubation, the spread plate technique was employed to inoculate agar plates with each microorganism from the respective broth cultures, ensuring proper colony distribution and isolation. Bacterial morphology was subsequently confirmed *via* optical microscopy. For MIC testing preparation, a few colonies from each agar plate were transferred into a sterile saline solution (8.5 g/L NaCl). The resulting suspension was adjusted to match the turbidity of a 0.5 McFarland standard, equivalent to approximately 1.5 × 10^8^ CFU/mL.

#### 4.5.2 Determination of the minimum inhibitory concentration (MIC)

The MIC was determined by the resazurin microtiter plate-based antibacterial/antifungal assay (Ștefănescu et al., 2024). In each well of a 96-well microtiter plate, 100 µL of sterile medium (specific to each microorganism) and 100 µL of the sample were added. Serial two-fold dilutions were performed across the wells of each row by transferring 100 µL from well to well, discarding 100 µL from the final well. To each well, 10 µL of inoculum was added, with a concentration of 1.5 × 10^5^ CFU/mL for bacterial strains and 10^6^ CFU/mL for the fungal strains. Norfloxacin (2 mg/mL in DMSO) served as the positive control for bacteria, while fluconazole (1 mg/mL in DMSO) was used as the positive control for yeast. The negative control was DMSO. The plates were incubated at 37 °C for bacteria for 20–22 h. After incubation, 20 µL of 0.2 mg/mL resazurin aqueous solution was added to each well, followed by an additional incubation period of 2 h at 37 °C. At the end of this period, the viable bacterial cells caused the resazurin (initially blue and non-fluorescent) to oxidize into resorufin (pink and fluorescent) within the wells. Thus, the concentration in the last well of each row that retained a blue color signified the complete inhibition of bacterial growth, indicating the MIC. MIC values were recorded as the lowest concentration at which no color change from blue to pink occurred, indicating complete inhibition of bacterial or fungal growth. Each sample was tested in duplicate to ensure accuracy.

### 4.6 Molecular dynamics simulation and structural analysis

The 3D structure of the peptides **1–2** and their conjugates **1a-b** and **2a-b**, were generated with Avogadro 1.2.0 and were minimized in vacuum using the steepest descent using MMFF94 (Halgren, 1996). For analyzing the conformations of the parent peptides 1 and 2 and their conjugates 1a-b and 2a-b in time in water [TIP3P model (Jorgensen et al., 1983)], a 200 ns molecular dynamics study using CHARMM36 force field was made using GROMACS 2025.4, after energy minimization, NVT for 500 ps and NPT for 500 ps (Abraham et al., 2015; Vanommeslaeghe et al., 2010; Vanommeslaeghe and MacKerell, 2012). The parametrizations were made using CHARMM-GUI (Brooks et al., 2009; Jo et al., 2008; Kim et al., 2017; Lee et al., 2016). The calculations were performed on a machine with AMD Ryzen™ 97,900 CPU and an NVIDIA RTX 3060 GPU with CUDA 12.8. running a Debian Linux 12. The visual analyses of the trajectories were visualized using VMD 1.9.4 (Humphrey et al., 1996). Numeric analysis of the trajectories was computed using the GROMACS built-in functions. Solvation free energy estimations were computed using *sasa* module with *odg* flag, based on atomic solvation parameters, while peptide–water non-bonded interaction energies (Coulomb and Lennard-Jones short-range terms) were computed using energy module (Eisenberg and McLachlan, 1986). Clustering of the conformations was made using cluster module, using gromos method. Spartan 24 1.1.0 (Wavefunction, Inc., Irvine, CA, United States) was used for obtaining some molecular descriptors of the compounds on a machine with Intel 12700 KF CPU, by DFT calculation using ΩB97X-D functional with 6-31G* basis set in water, using the Conductor-like Polarizable Continuum Model.

The cLogP values were calculated *in silico* using ChemDraw Professional, version 22.2.0, from the structures of the parent peptides and conjugates, as a preliminary indicator of aqueous solubility-relevant lipophilicity.

## Data Availability

The original contributions presented in the study are included in the article/Supplementary Material, further inquiries can be directed to the corresponding author.
