# Supplementary material for: Synthesis, molecular dynamics, and antimicrobial evaluation of peptide-antibiotic conjugates designed for dual-mechanism pathogen inhibition
Source: Front Pharmacol. 2026 Sep 17;17:1927384. doi: 10.3389/fphar.2026.1927384 (PMC13628211; doi:10.3389/fphar.2026.1927384)
Supplement: Supplementary file 1 [file DataSheet1.pdf]

Supplementary Material

Synthesis, Molecular Dynamics, and Antimicrobial Evaluation of Peptide-Antibiotic Conjugates Designed for Dual-Mechanism Pathogen Inhibition

Denisa Leonte[1], Clemence Fartaoui[1], Ioana Ionuț[2], Róbert Tőtős[3], Gabriel Marc[1*], Laurian Vlase[4], Gheorghe-Adrian Martău[5,6], Dan Cristian Vodnar[5,6], Ovidiu Crișan[1]

[1]Department of Organic Chemistry, "Iuliu Hațieganu" University of Medicine and Pharmacy, 41 Victor Babeș Street, 400012 Cluj-Napoca, Romania, *[2]Department of Pharmaceutical Chemistry, "Iuliu Hațieganu" University of Medicine and Pharmacy, 41 Victor Babeș Street, 400012 Cluj-Napoca, Romania*, *[3]Enzymology and Applied Biocatalysis Research Center, Faculty of Chemistry and Chemical Engineering, Babeș-Bolyai University, Arany János Street 11, 400028, Cluj-Napoca, Romania*, *[4]Department of Pharmaceutical Technology and Biopharmacy, "Iuliu Hațieganu" University of Medicine and Pharmacy, 41 Victor Babeș Street, 400012 Cluj-Napoca, Romania*, *[5]Institute of Life Sciences, University of Agricultural Sciences and Veterinary Medicine, 3-5 Calea Mănăștur, 400372 Cluj-Napoca, Romania*, *[6]Faculty of Food Science and Technology, University of Agricultural Sciences and Veterinary Medicine, 3-5 Calea Mănăștur, 400372 Cluj-Napoca, Romania*

TABLE OF CONTENTS

Synthesis of N-tert-butoxycarbonyl-norfloxacin (compound **a**)……….……………………….….2

Synthesis of metronidazole hemisuccinate monoester (compound **b**)………….…………………..3

Synthesis of antimicrobial peptides **1** and **2** ……………………...…………………………….4

Schematic representation of norfloxacin/metronidazole conjugation of peptides **1** and **2**…………..7

HRMS of the final antibiotic-peptide conjugates **1a,b** and **2a,b**……………….…………………….…11

Molecular dynamic simulations…………………………………………………………………15

Synthesis of N-tert-butoxycarbonyl-norfloxacin (compound a)

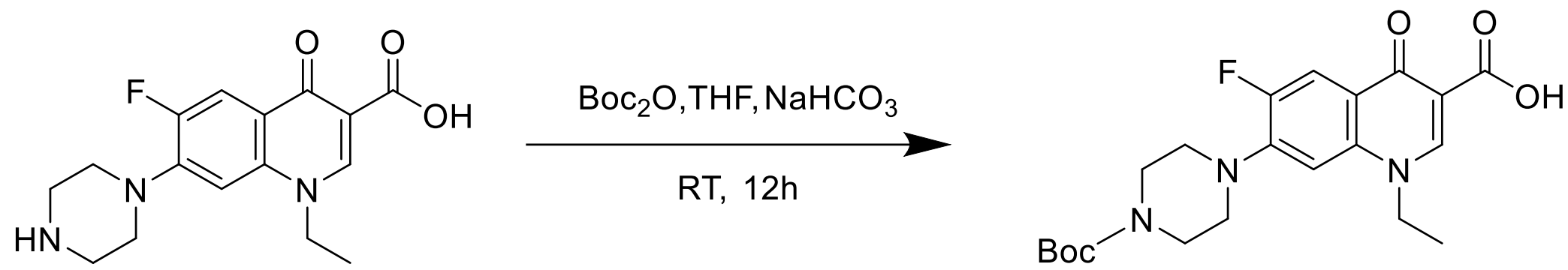

Norfloxacin (639 mg, 2.0 mmol, 1.0 equiv.) was dissolved in a mixture of tetrahydrofuran (12 mL) and water (6 mL) at pH 8-9, adjusted with sodium bicarbonate (504 mg, 6.0 mmol). Di-tert-butyl dicarbonate (Boc_2O, 552 mL, 2.4 mmol) was added and the reaction mixture was stirred at room temperature overnight. After completion o fthe reaction, monitored by RP-HPLC, THF was removed under reduced pressure and the pH was adjusted to 3 with citric acid. The formed precipitate was collected by filtration under reduced pressure to afford compound **a** as white powder, in 77 % yield. Retention time: 6.0 min (RP-HPLC separation conditions: Phenomenex Gemini C18 column, 3 μM, 110 Å, 50 x 3.00 mm i.d., maintained at 40 ºC, mobile phase: 50% acetonitrile and 50% water, 0.2 mL/min flow rate, UV detection at 214 and 279 nm); Rf = 0.46 (mobile phase CH_2Cl_2/Acetone 50/50 *v/v*). ^{1}H NMR and ^{13}C NMR spectra are identical to data already reported in the literature.

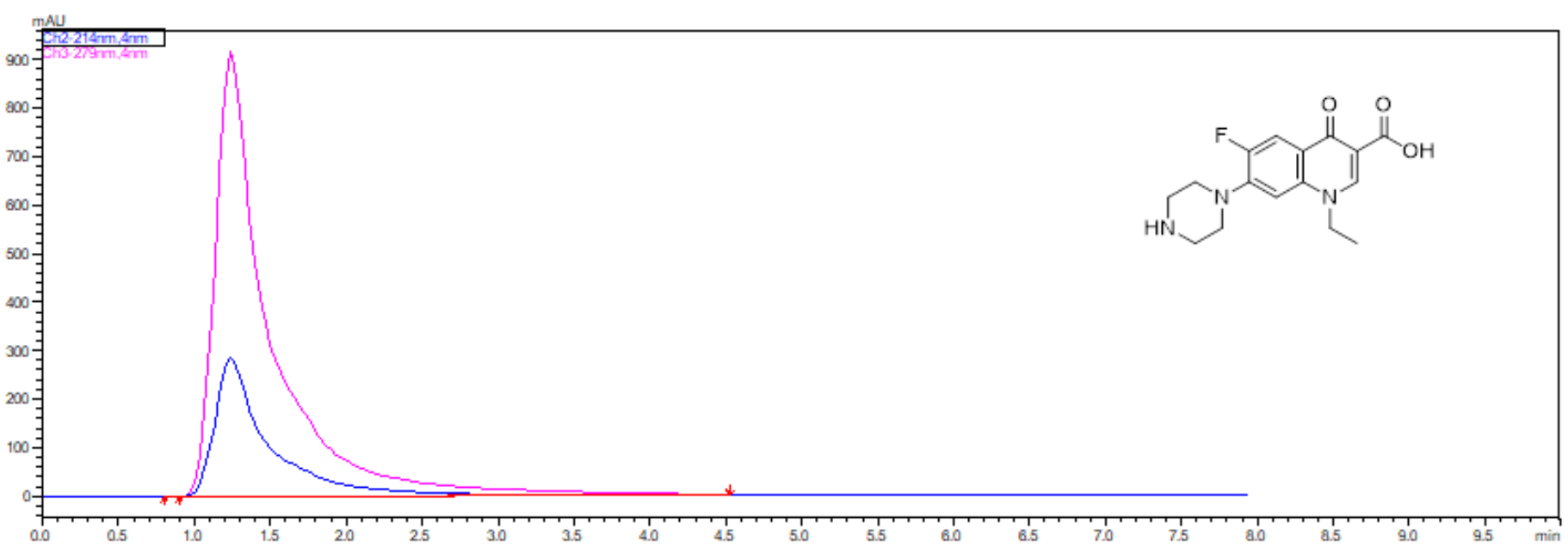

Supplementary Figure 1. Chromatogram of norfloxacin. RP-HPLC conditions: Phenomenex Gemini C18 column, 3 μM, 110 Å, 50 x 3.00 mm i.d., maintained at 40 ºC, mobile phase: acetonitrile/water 50/50 v/v, 0.2 mL/min flow rate, UV detection at 214 and 279 nm.

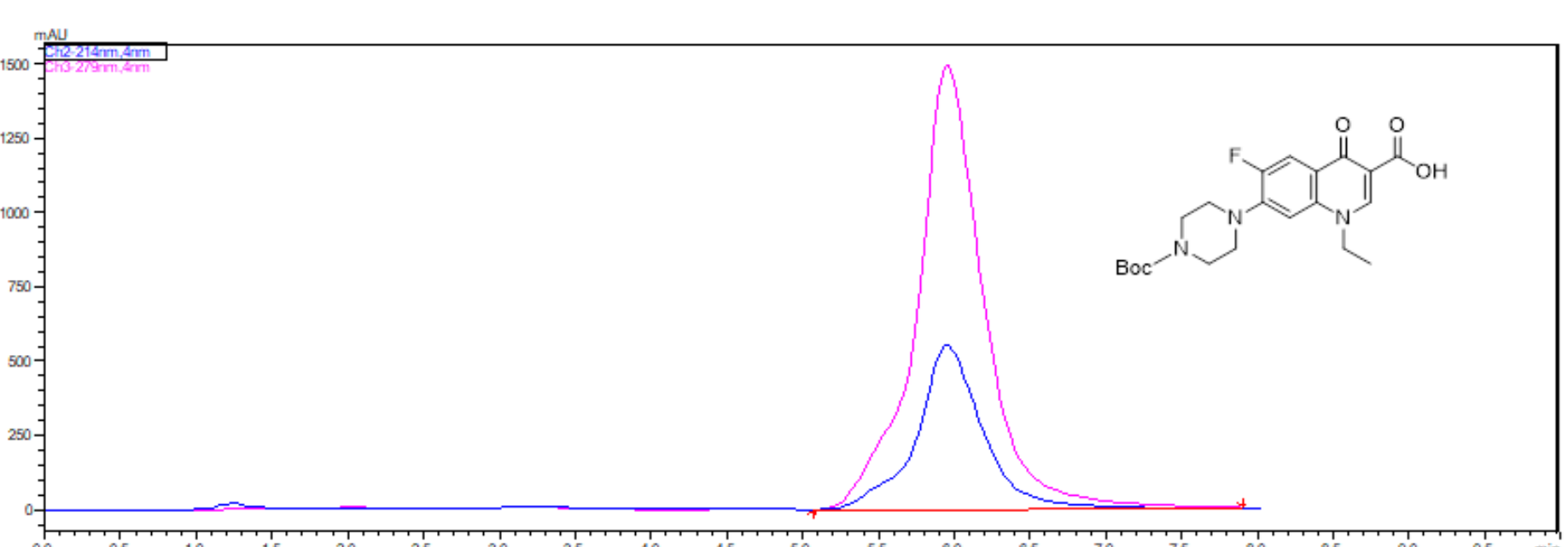

Supplementary Figure 2. Chromatogram of N-tert-butoxycarbonyl norfloxacin (compound **a**). RP-HPLC conditions: Phenomenex Gemini C18 column, 3 μM, 110 Å, 50 x 3.00 mm i.d., maintained at 40 ºC, mobile phase: acetonitrile/water 50/50 v/v, 0.2 mL/min flow rate, UV detection at 214 and 279 nm.

Synthesis of metronidazole hemisuccinate monoester (compound b)

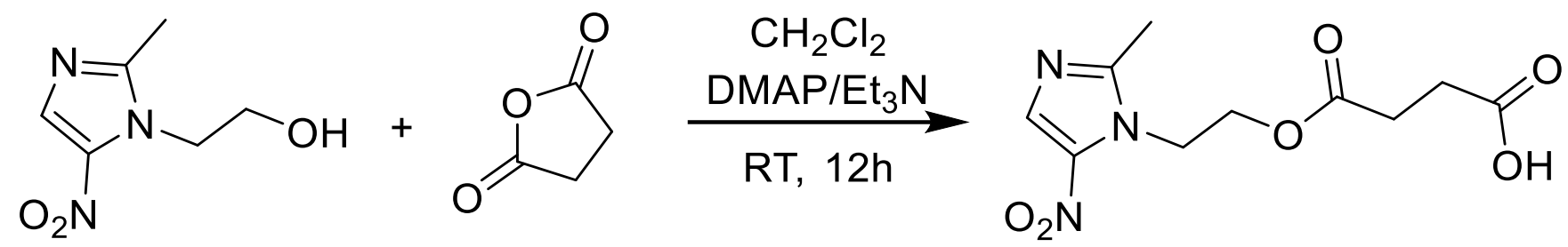

To a solution of metronidazole (513 mg, 3.0 mmol) in anhydrous dichloromethane (10 mL), succinic anhydride (450 mg, 4.5 mmol) was added, followed by addition of a catalytic amount of 4-N,N-dimethylaminopyridine (DMAP, 73 mg, 0.6 mmol) and triethylamine (585 µL, 4.2 mmol). The reaction mixture was stirred overnight at room temperature under magnetic stirring. After completion of the reaction, the reaction mixture was quenched for 20 min with water (15 mL) in order to hydrolyse the excess of succinic anhydride. The aqueous phase was removed and the organic phase was washed three times with water (3x15 mL). The organic phase was dried over anhydrous Na_2SO_4 and the solvent was evaporated to dryness under reduced pressure. The crude product was purified by silica gel column chromatography using a mixture of dichloromethane and acetone as mobile phase, in a gradient separation procedure, from 10% to 50% acetone to afford **compound b** as a white solid with 80% yield. R_t: 4 min (RP-HPLC separation conditions: Phenomenex Luna C18, 5 µM, 100 Å, 150 x 4.6 mm, maintained at 40 ºC, mobile phase: 50% acetonitrile and 50% water, flow rate 0.4 mL/min); Rf = 0.4 (mobile phase CH_2Cl_2/Acetone 50/50 *v/v*). ^{1}H NMR and ^{13}C NMR spectra of the final product are identical to those already reported in the literature.

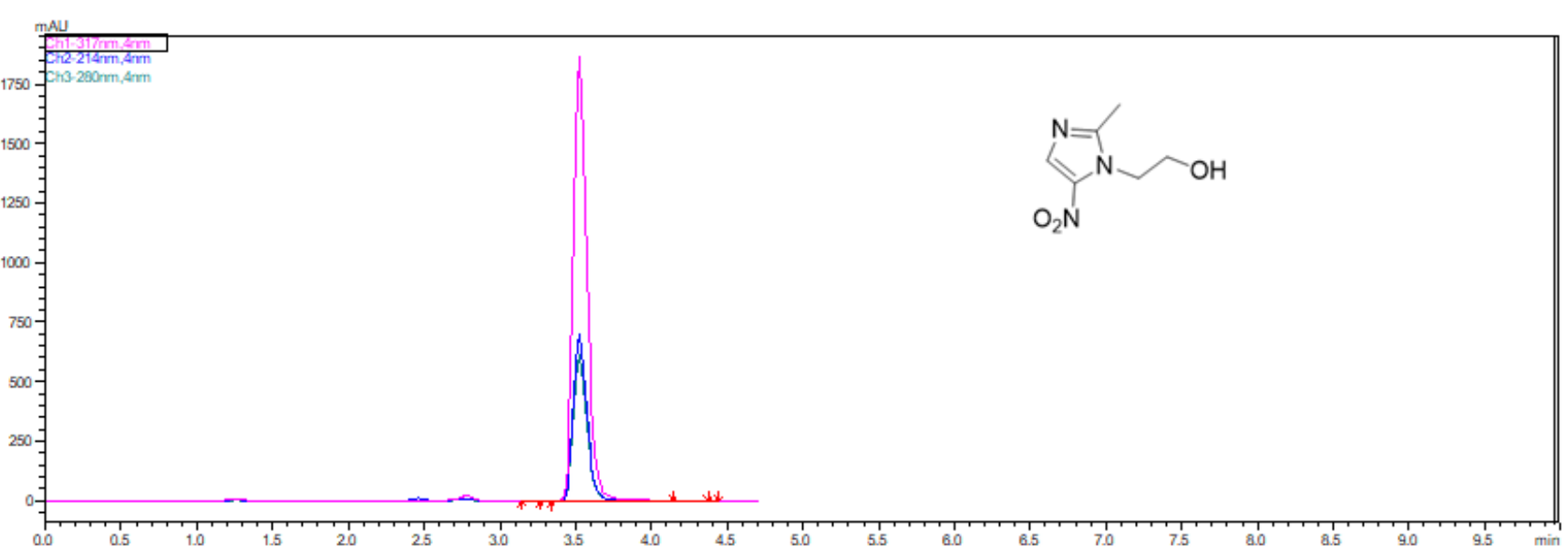

Supplementary Figure 3. Chromatogram of metronidazole. RP-HPLC conditions: Phenomenex Luna C18, 5 µM, 100 Å, 150 x 4.6 mm, maintained at 40 ºC, mobile phase: acetonitrile/water 50/50 v/v, flow rate 0.4 mL/min).

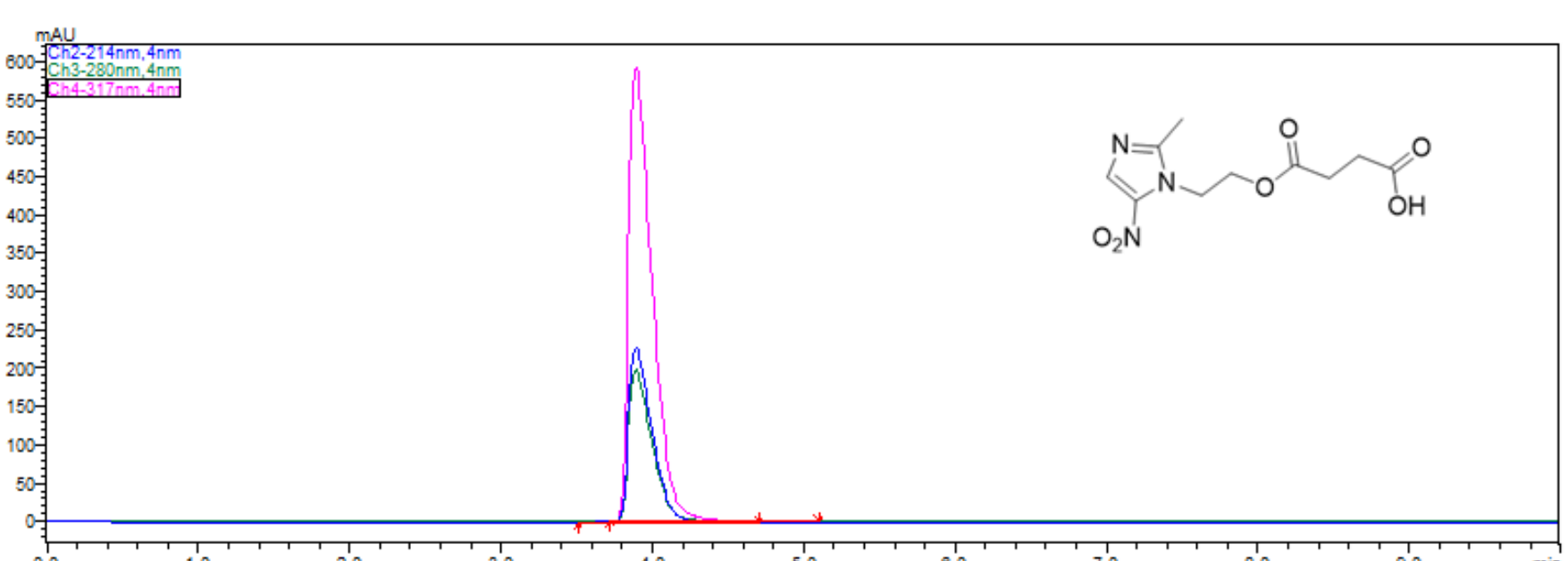

Supplementary Figure 4. Chromatogram of **compound b**. RP-HPLC conditions: Phenomenex Luna C18, 5 µM, 100 Å, 150 x 4.6 mm, maintained at 40 ºC, mobile phase: acetonitrile/water 50/50 v/v, flow rate 0.4 mL/min.

Synthesis of peptide 1

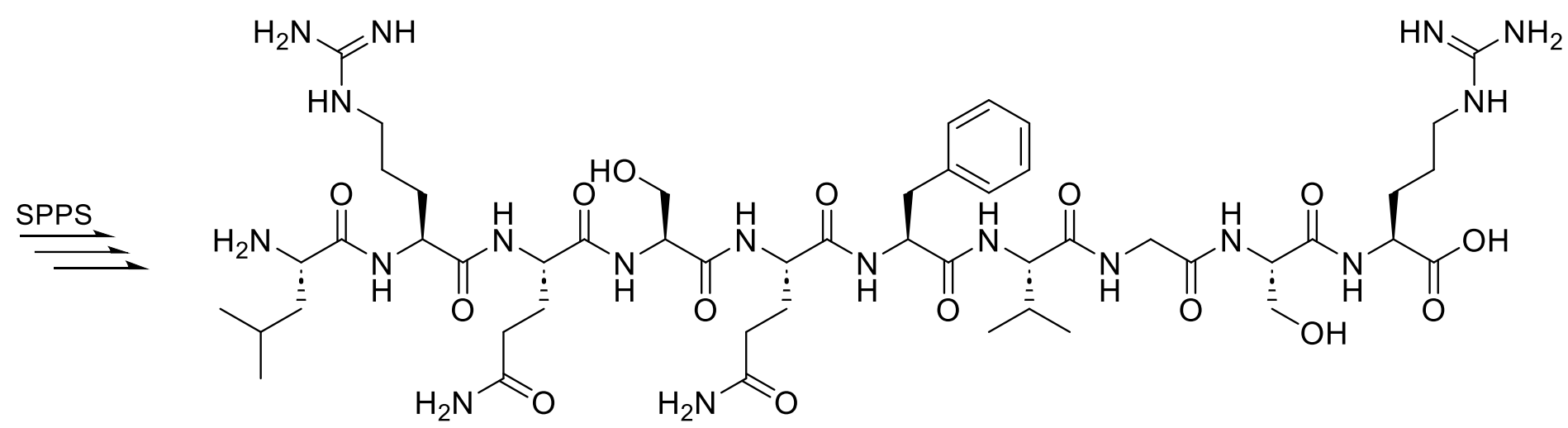

The peptide **LRQSQFVGSR** was synthesized by standard Fmoc-based solid-phase peptide synthesis (SPPS) on 2-chlorotrityl chloride (2-CTC) resin. The resin (400 mg) was swollen in 5 mL dichloromethane (DCM) for 30 min, then filtered to remove excess solvent.

N-Fmoc-L-Arg(Pbf)-OH (454 mg, 0.7 mmols, 1.2 equiv.) was pre-activated for 10 min by shaking at 600 rpm, in dichloromethane (3 mL) containing N,N-diisopropylethylamine (DIPEA, 304 µL, 1.75 mmols, 2.5 equiv.), then added to the resin. The reaction mixture was gently shaken at 300 rpm. After 1 hour, an additional portion of DIPEA (304 µL, 1.75 mmols, 2.5 equiv.) was added to the resin. After 2 hours of gently shaking at 300 rpm, methanol (800µL) was added to the reaction mixture under continuous shaking for 15 min, for capping the unreacted sites of resin. Then, the resin was washed sequentially with N,N-dimethylformamide (DMF, 3 x 3 mL), dichloromethane (3 x 3 mL), then methanol/dichloromethane (1 :1 v/v, 3 x 3 mL), and finally methanol (3 x 3 mL). The resin was dried under vacuum and accurately weighed to determine its substitution level, wich was subsequently used to calculate the precise mmol of each amino acid required for all further coupling steps.

Fmoc deprotection was carried out by treatment with 20% piperidine in DMF, in two cycles (2x3mL) under continuous shaking at 300 rpm: first deprotection cycle (5 min) followed by washing with DMF (3 mL), followed by second deprotection cycle (15 min). The resin was washed five times with DMF (5 x 3 mL). Each Fmoc deprotection was checked by a positive Kaiser test.

The next amino acids were sequentially coupled in the following order, C- to N-terminus: Fmoc-L-Ser(tBu)-OH, Fmoc-Gly-OH, Fmoc-L-Val-OH, Fmoc-L-Phe-OH, Fmoc-L-Gln(Trt)-OH, Fmoc-L-Ser(tBu)-OH, Fmoc-L-Gln(Trt)-OH, Fmoc-L-Arg(Pbf)-OH and Fmoc-L-Leu-OH. Each amino acid (3 eq.) was activated prior to coupling by treatment with with N,N'-diisopropylcarbodiimide (DIC, 3 equiv.) and and ethyl 2-cyano-2-(hydroxyimino)acetate (Oxyma Pure, 3 equiv.) in DMF (3 mL) under continuous shaking at 600 rpm for 10 min. Coupling reactions were performed in DMF, between 60 min and 240 min, under continuous gentle agitation (300 rpm). After each coupling step, the resin was washed five times with DMF (5 x 3 mL). The completion of each coupling reaction was confirmed by a negative Kaiser test.

After the last Fmoc removal, the resin was rinsed with DCM (5 x 3mL, 2 min) and dried under reduced pressure at room temperature. Then the resin was treated with 2 mL of cleavage cocktail freshly prepared (TFA/H_2O/TIS, 95/2.5/2.5 *v/v/v*). The mixture was shaken for 3 h and the solution was added dropwise to cold diethylether (30 mL). The resin was treated once again with 2 mL of cleavage cocktail, followed by precipitation in cold diethylether. The formed precipitate was separated by centrifugation (3000 rpm, 5 min). The supernatant was discarded and the solid was washed twice with cold diethyl ether (2 x 10 mL) and then centrifuged again (3000 rpm, 5 min). The crude product obtained was air-dried (4 h) at room temperature, dissolved in a minimum amount of water and freeze dried. Crude

peptides were purified by semi preparative HPLC on a RP-C18 column as mentioned in **Section 4.1.2**. Pure fractions were combined, lyophilized. The purified products were dissolved in 0.05 M aqueous HCl solution, at a concentration of 10 mg/mL. The obtained solutions were lyophilized, in order to obtain pure peptides as hydrochloride salts.

Synthesis of peptide 2

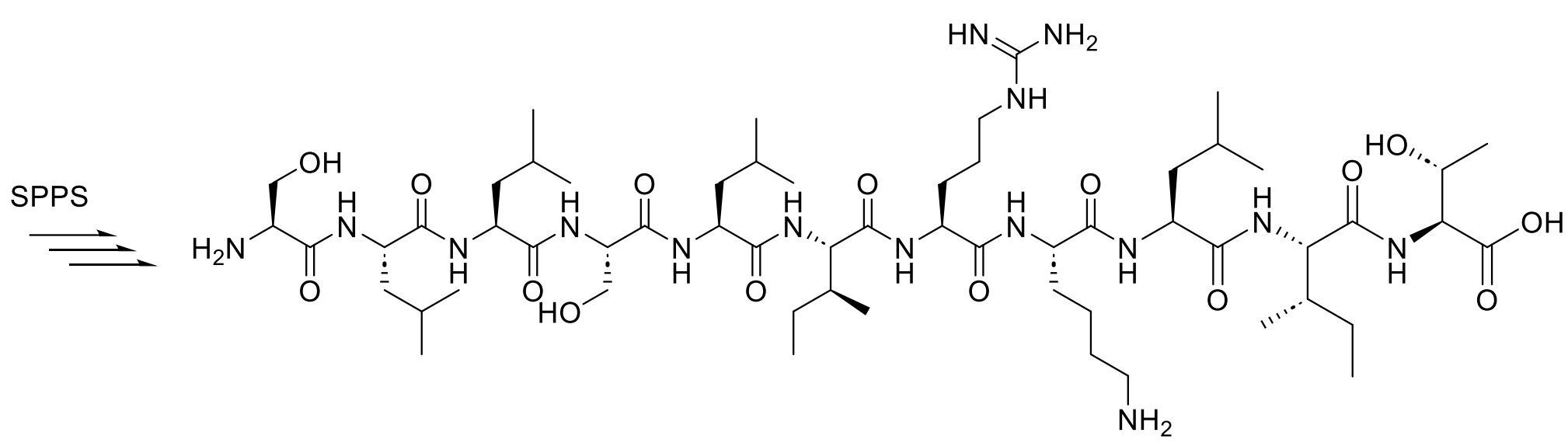

The peptide **SLLSLIRKLIT** was synthesized by standard Fmoc-based SPPS on 2-chlorotrityl chloride (2-CTC) resin. The resin (400 mg) was swollen in dichloromethane (DCM) for 30 min, then filtered to remove excess solvent.

Fmoc-L-Thr(tBu)-OH (268 mg, 0.7 mmols, 1.2 equiv.) was pre-activated for 10 min by shaking at 600 rpm, in dichloromethane (2 mL) containing N,N-diisopropylethylamine (DIPEA, 304 µL, 1.75 mmols, 2.5 equiv.), then added to the resin. The reaction mixture was gently shaken at 300 rpm. After 1 hour, an additional portion of DIPEA (304 µL, 1.75 mmols, 2.5 equiv.) was added to the resin. After 2 hours of gently shaking at 300 rpm, methanol (800µL) was added to the reaction mixture under continuous shaking for 15 min, for capping the unreacted sites of resin. Then, the resin was washed sequentially with DMF (3 x 3 mL), DCM (3 x 3 mL), then methanol/DCM (1 :1 v/v, 3 x 3 mL), and finally methanol (3 x 3 mL). The resin was dried under vacuum and accurately weighed to determine its substitution level, wich was subsequently used to calculate the precise mmol of each amino acid required for all further coupling steps.

Fmoc deprotection was carried out by treatment with 20% Piperidine in DMF, in two cycles (2x3mL) under continuous shaking at 300 rpm: first deprotection cycle (5 min) followed by washing with DMF (3 mL), followed by second deprotection cycle (15 min). The resin was washed five times with DMF (5 x 3 min). Each Fmoc deprotection was checked by a positive Kaiser test.

The next amino acids were sequentially coupled in the following order, C- to N-terminus: Fmoc-L-Ile-OH, Fmoc-L-Leu-OH, Fmoc-Lys(Boc)-OH, Fmoc-L-Arg(Pbf)-OH, Fmoc-Ile-OH, Fmoc-L-Leu-OH, Fmoc-L-Ser(tBu)-OH, Fmoc-L-Leu-OH, Fmoc-L-Leu-OH and Fmoc-L-Ser-(tBu)-OH. Each amino acid (3 eq.) was activated prior to coupling by treatment with with N,N'-diisopropylcarbodiimide (DIC, 3 equiv.) and and ethyl 2-cyano-2-(hydroxyimino)acetate (Oxyma Pure, 3 equiv.) in DMF (3 mL) under continuous shaking at 600 rpm for 10 min. Coupling reactions were performed in DMF, between 60 min and 240 min, under continuous gentle agitation (300 rpm). After each deprotection step, the resin was washed five times with DMF (5 x 3 mL). The completion of each coupling reaction was confirmed by a negative Kaiser test.

After the last Fmoc removal, the resin was rinsed with DCM (5 x 3mL, 2 min) and dried under reduced pressure at room temperature. Then the resin was treated with 2 mL of cleavage cocktail freshly

prepared (TFA/H_2O/TIS, 95/2.5/2.5 *v/v/v*). The mixture was shaken for 3 h and the solution was added dropwise to cold diethylether (30 mL). The resin was treated once again with 2 mL of cleavage cocktail, followed by precipitation in cold diethylether. The formed precipitate was separated by centrifugation (3000 rpm, 5 min). The supernatant was discarded and the solid was washed twice with cold diethyl ether (2 x 10 mL) and then centrifuged again (3000 rpm, 5 min). The crude product obtained was air-dried (4 h) at room temperature, dissolved in a minimum amount of water and freeze dried. Crude peptides were purified by semi preparative HPLC on a RP-C18 column as mentioned in **Section 4.1.2**. Pure fractions were combined, lyophilized. The purified products were dissolved in 0.05 M aqueous HCl solution, at a concentration of 10 mg/mL. The obtained solutions were lyophilized, in order to obtain pure peptides as hydrochloride salts.

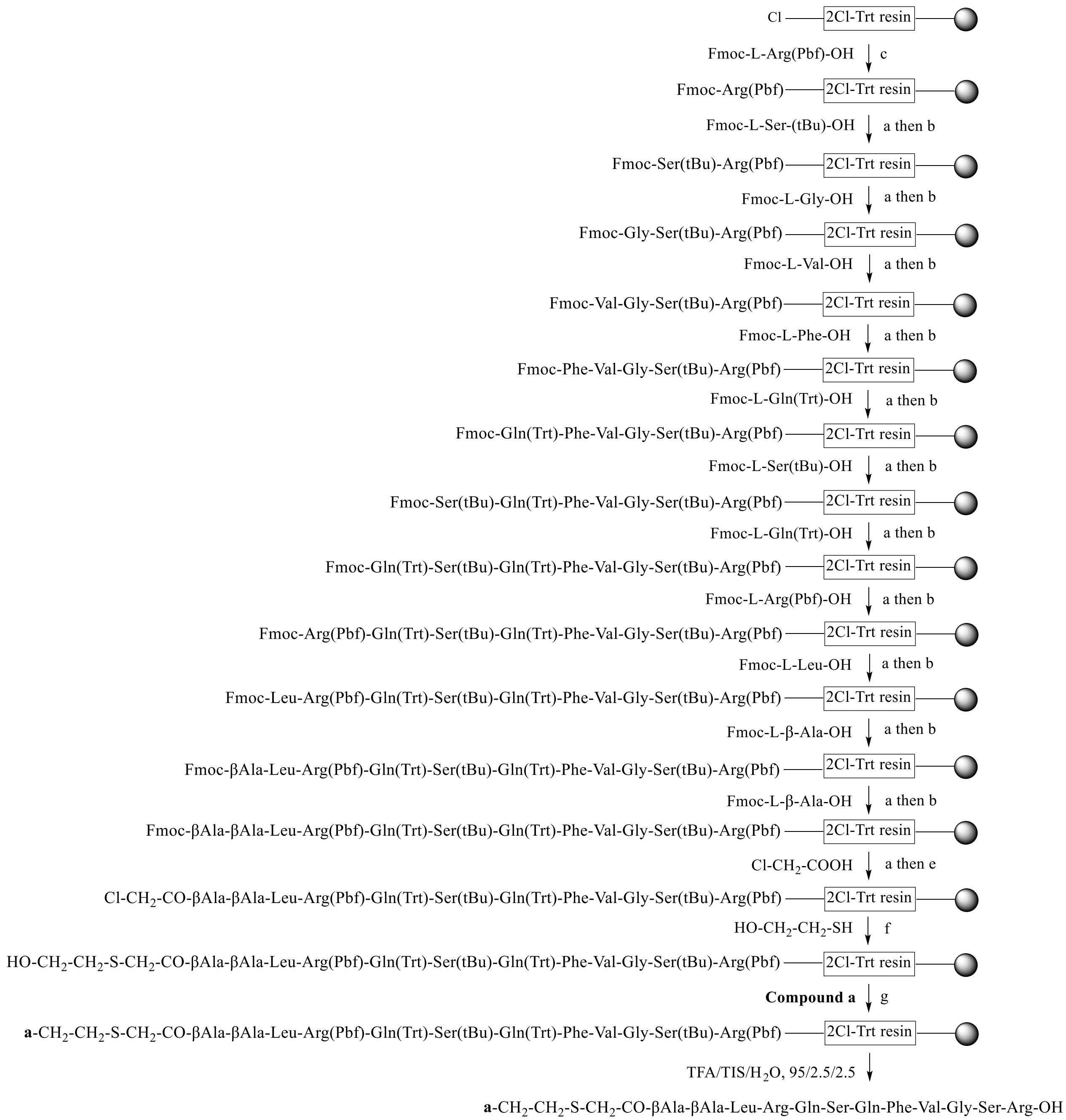

1a

Procedure a : 20% piperidine in DMF
Procedure b : Fmoc-amino acid (3 eq.), DIC(3 eq.), Oxyma (3 eq.) in DMF
Procedure c : resin in DCM (40 min), filtered, added Fmoc-L-Arg(Pbf)-OH (1,2 eq.) with DIPEA (2,5 eq.), 4h.
Procedure e : Cl-CH$_2$-COOH (5 eq.), DIC (5 eq.), in DMF, 1h30.
Procedure f : HO-CH$_2$-CH$_2$-SH (3 eq.), DIPEA (6 eq.), in DMF, overnight.
Procedure g : N-Boc-Norfloxacin **a** (3 eq.), HBTU (3 eq.), DMAP (1,5 eq.), DIPEA (6 eq.), in NMP, overnight.

Scheme S1a. Synthesis on solid support of norfloxacin-conjugated peptide **1a**. Final cleavage was performed in 3 h.

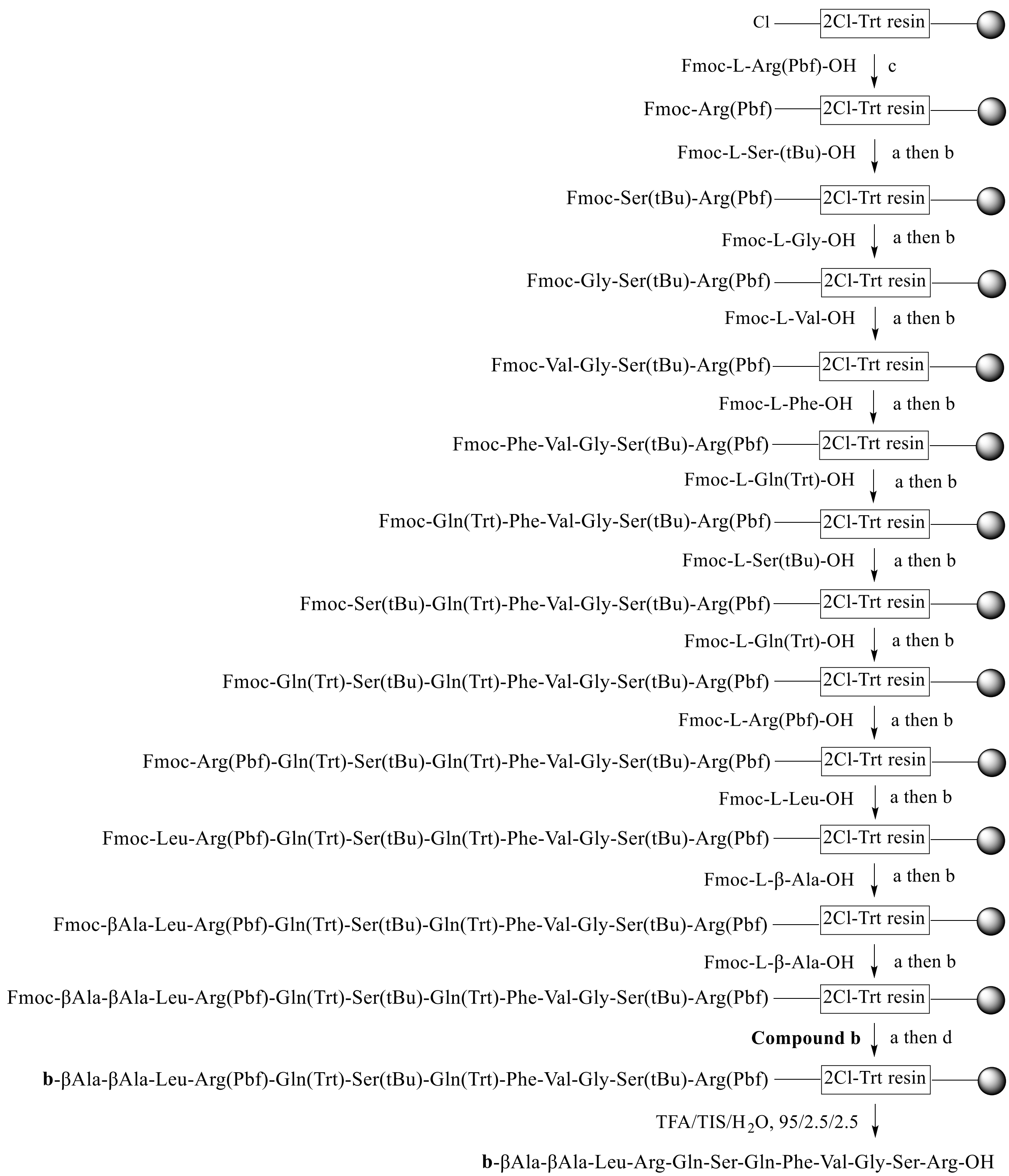

1b

Procedure a : 20% piperidine in DMF
Procedure b : Fmoc-amino acid (3 eq.), DIC(3 eq.), Oxyma (3 eq.) in DMF
Procedure c : resin in DCM (40 min), filtered, added Fmoc-L-Arg(Pbf)-OH (1,2 eq.) with DIPEA (2,5 eq.), 4h.
Procedure d : Metronidazole hemisuccinate monoester **b** (3 eq.), DIC (3 eq.), in DMF, overnight.

Scheme S1b. Synthesis on solid support of metronidazole-conjugated peptide **1b**. Final cleavage was performed in 3 h.

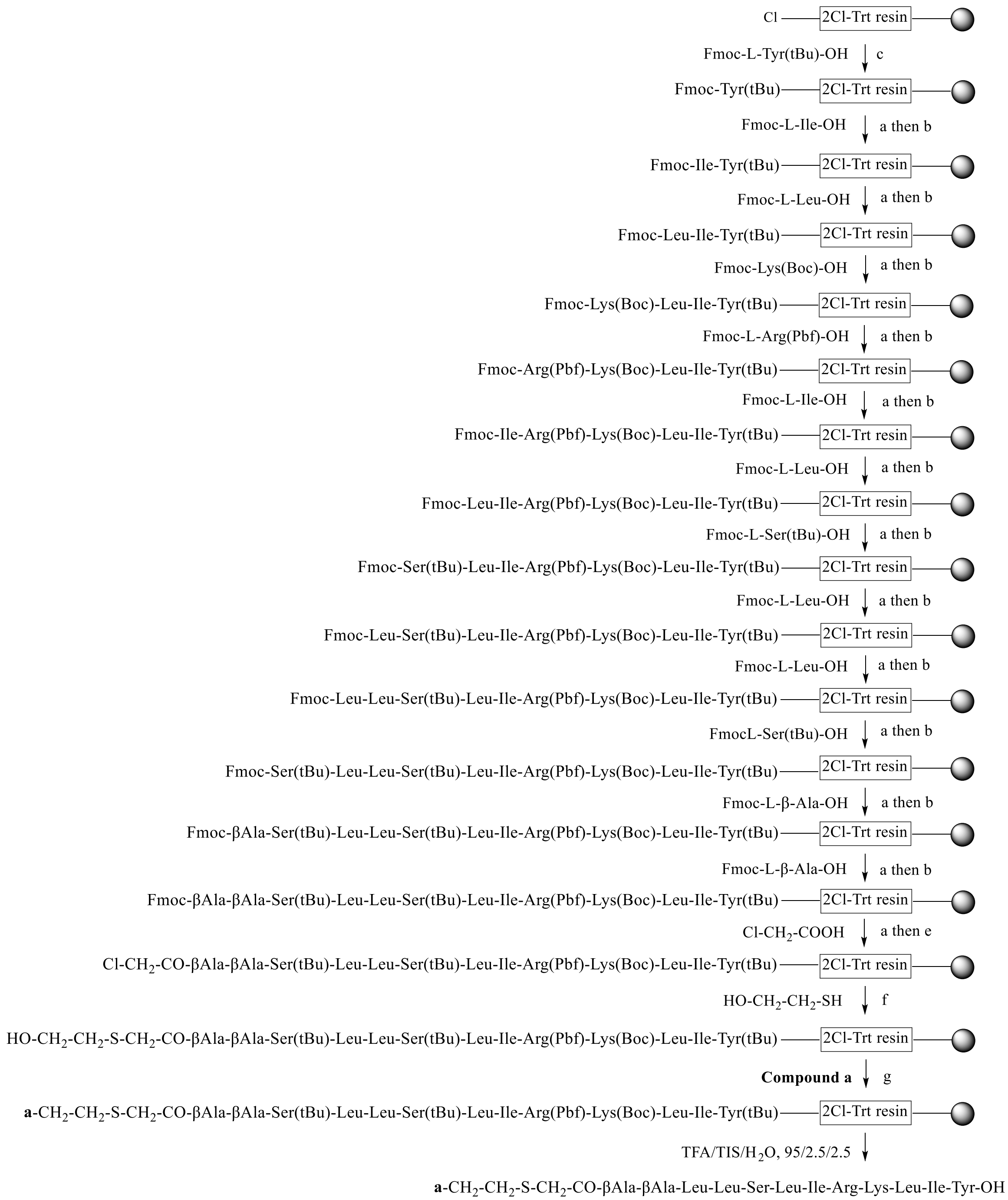

2a

Procedure a : 20% piperidine in DMF
Procedure b : Fmoc-amino acid (3 eq.), DIC(3 eq.), Oxyma (3 eq.) in DMF
Procedure c : resin in DCM (40 min), filtered, added Fmoc-L-Arg(Pbf)-OH (1,2 eq.) with DIPEA (2,5 eq.), 4h.
Procedure e : Cl-CH_2-COOH (5 eq.), DIC (5 eq.), in DMF, 1h30.
Procedure f : HO-CH_2-CH_2-SH (3 eq.), DIPEA (6 eq.), in DMF, overnight.
Procedure g : N-Boc-Norfloxacin **a** (3 eq.), HBTU (3 eq.), DMAP (1,5 eq.), DIPEA (6 eq.), in NMP, overnight.

Scheme S2a. Synthesis on solid support of norfloxacin-conjugated peptide **2a**. Final cleavage was performed in 3 h.

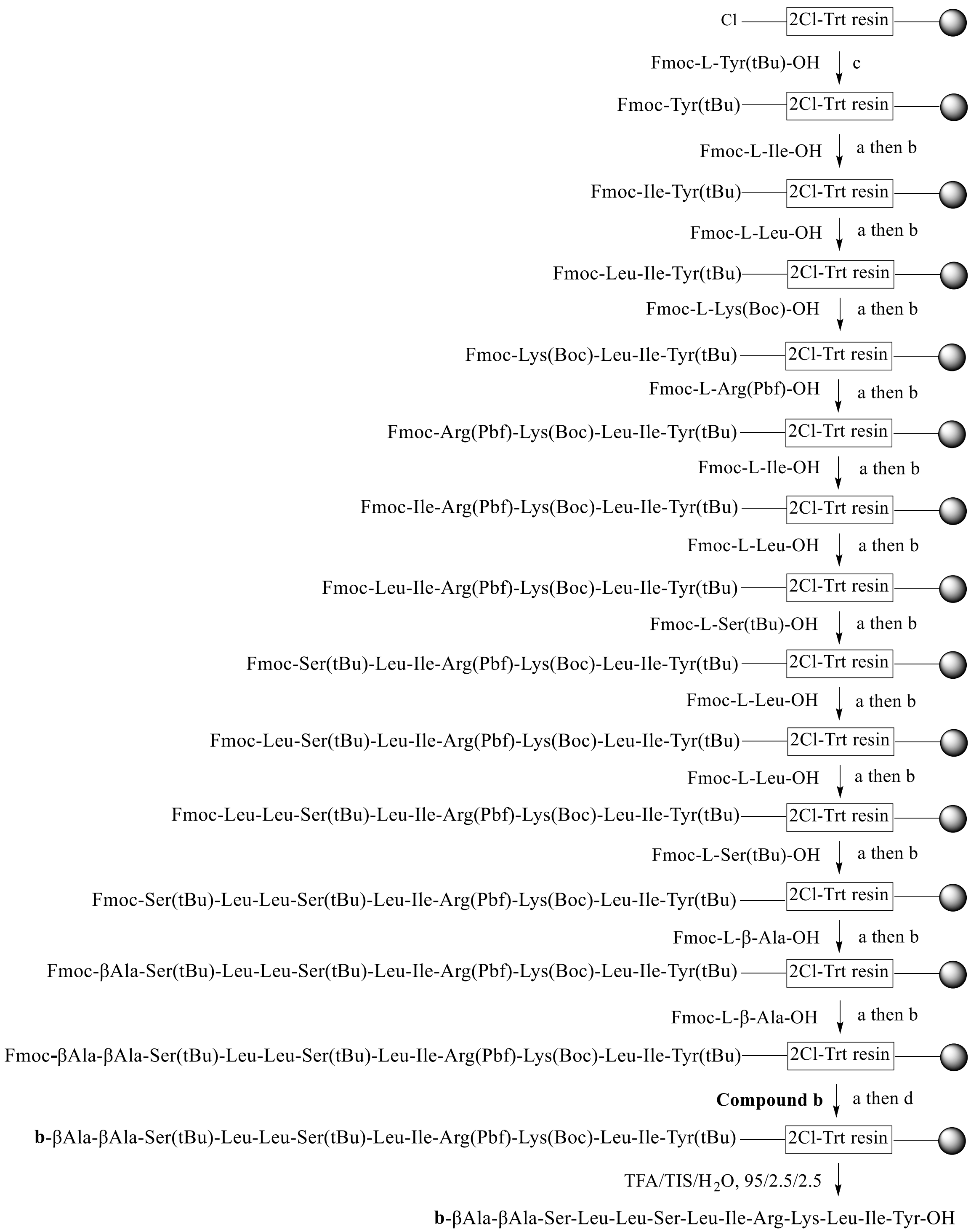

2b

Procedure a : 20% piperidine in DMF
Procedure b : Fmoc-amino acid (3 eq.), DIC(3 eq.), Oxyma (3 eq.) in DMF
Procedure c : resin in DCM (40 min), filtered, added Fmoc-L-Arg(Pbf)-OH (1,2 eq.) with DIPEA (2,5 eq.), 4h.
Procedure d : Metronidazole hemisuccinate monoester **b** (3 eq.), DIC (3 eq.), in DMF, overnight.

Scheme S2b. Synthesis on solid support of metronidazole-conjugated peptide **2b**. Final cleavage was performed in 3 h.

HRMS of the final antibiotic-peptide conjugates

Norfloxacin-conjugated peptide 1a:

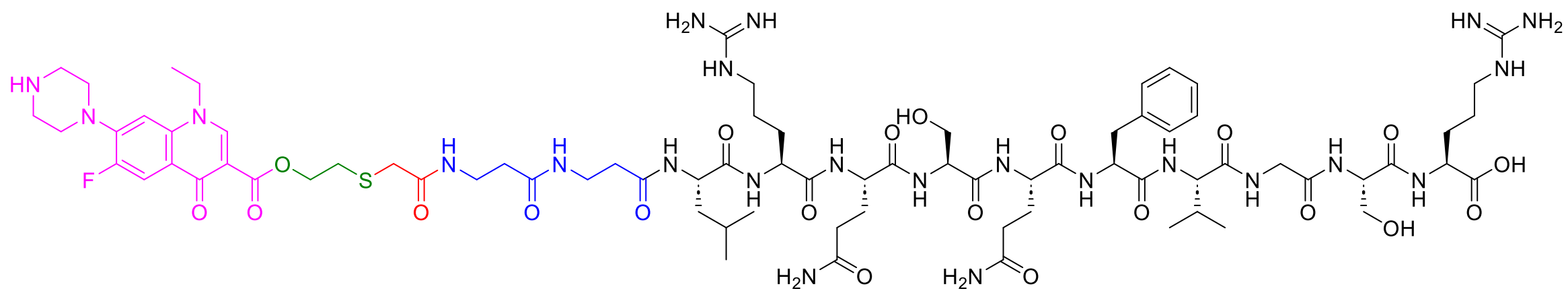

1a

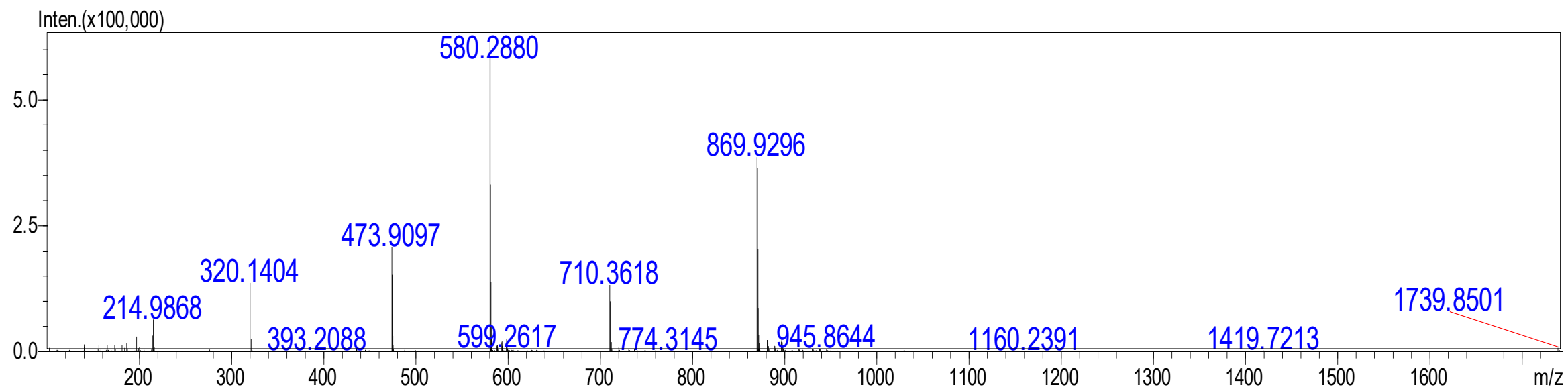

1a: detailed $[M+H]^+$:

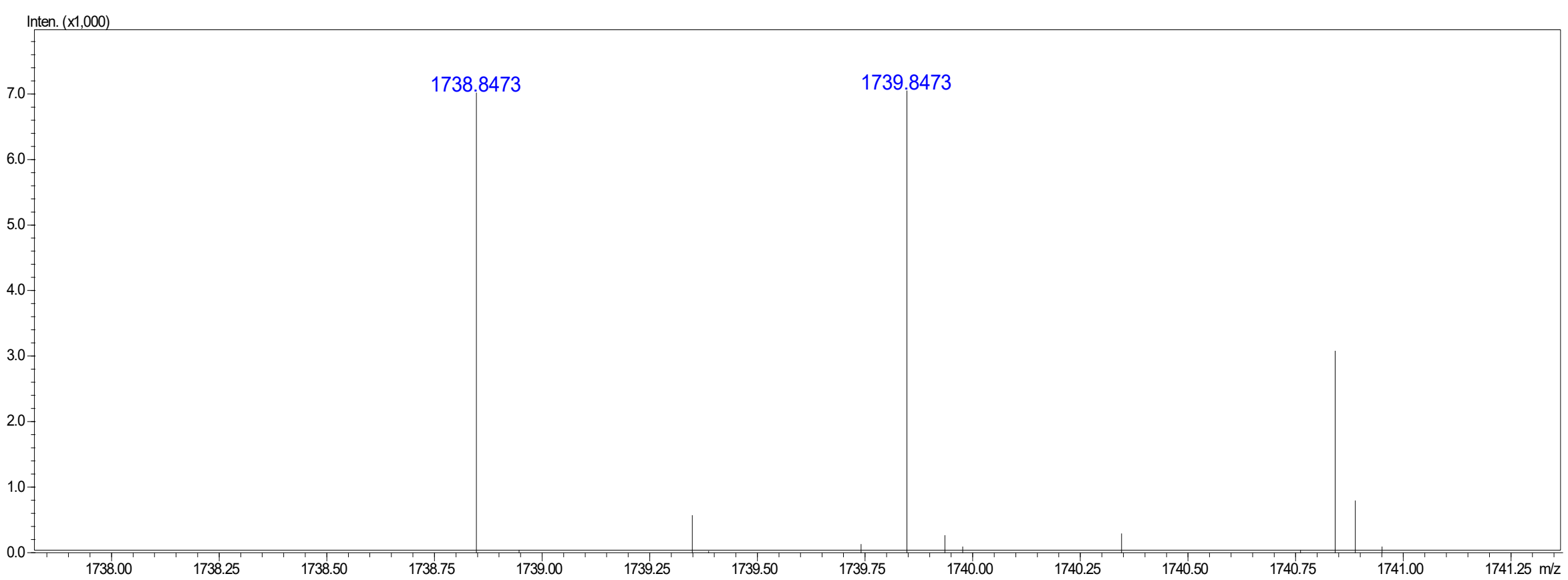

Norfloxacin-conjugated peptide 2a:

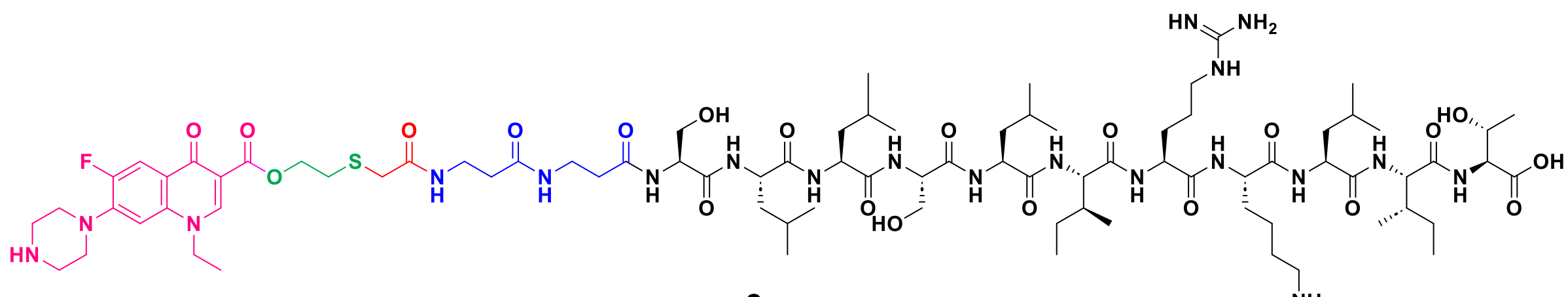

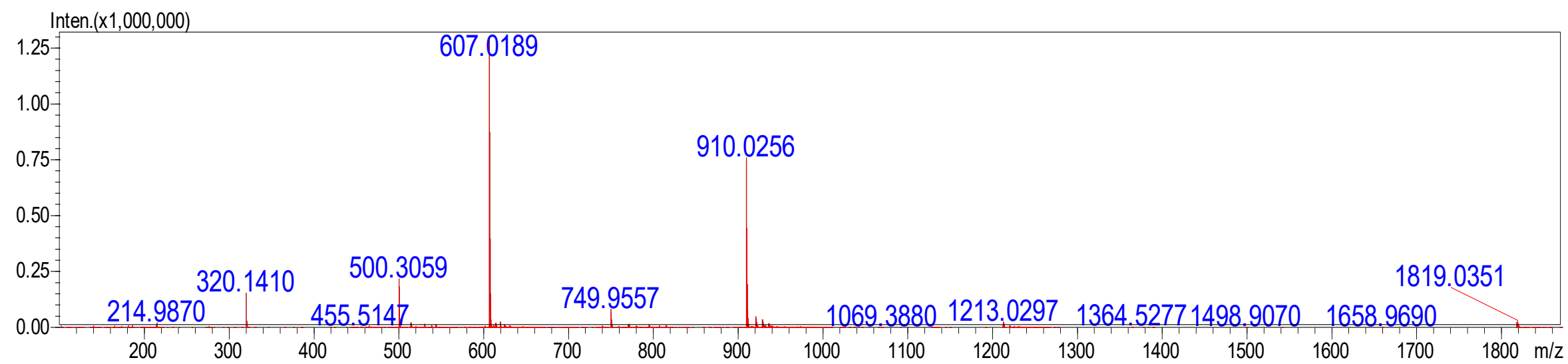

2a - detailed $[M+H]^+$:

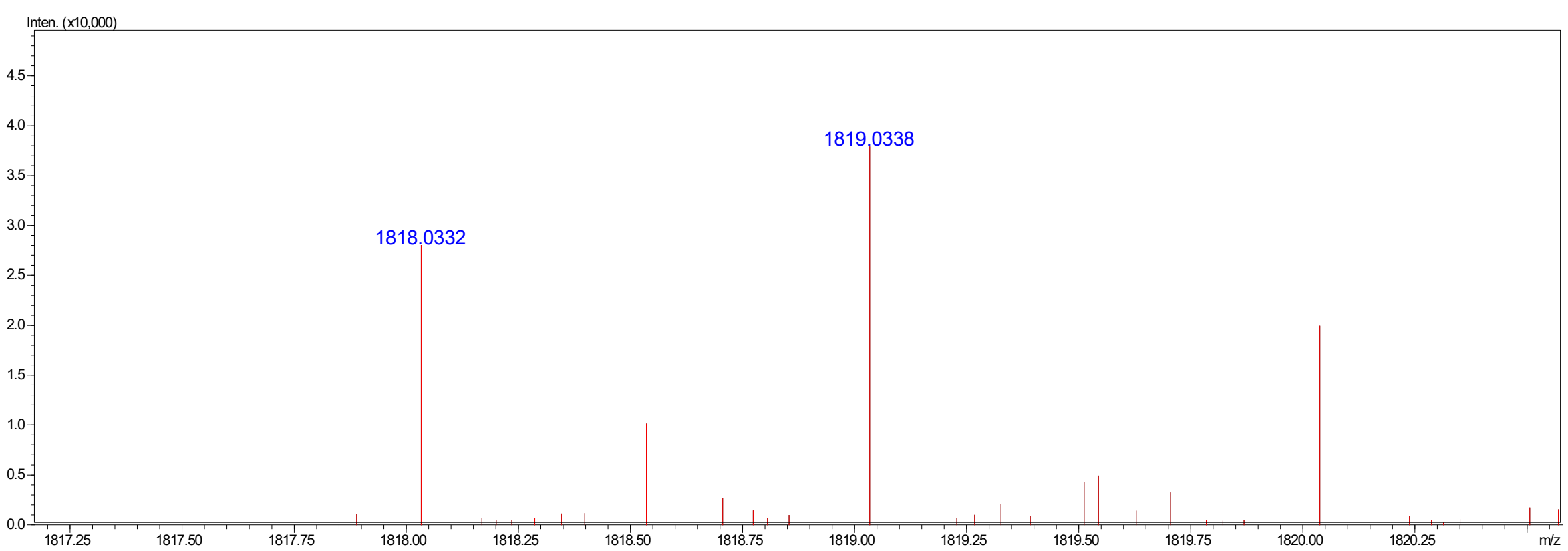

2a - detailed $[M+2H]^{2+}$:

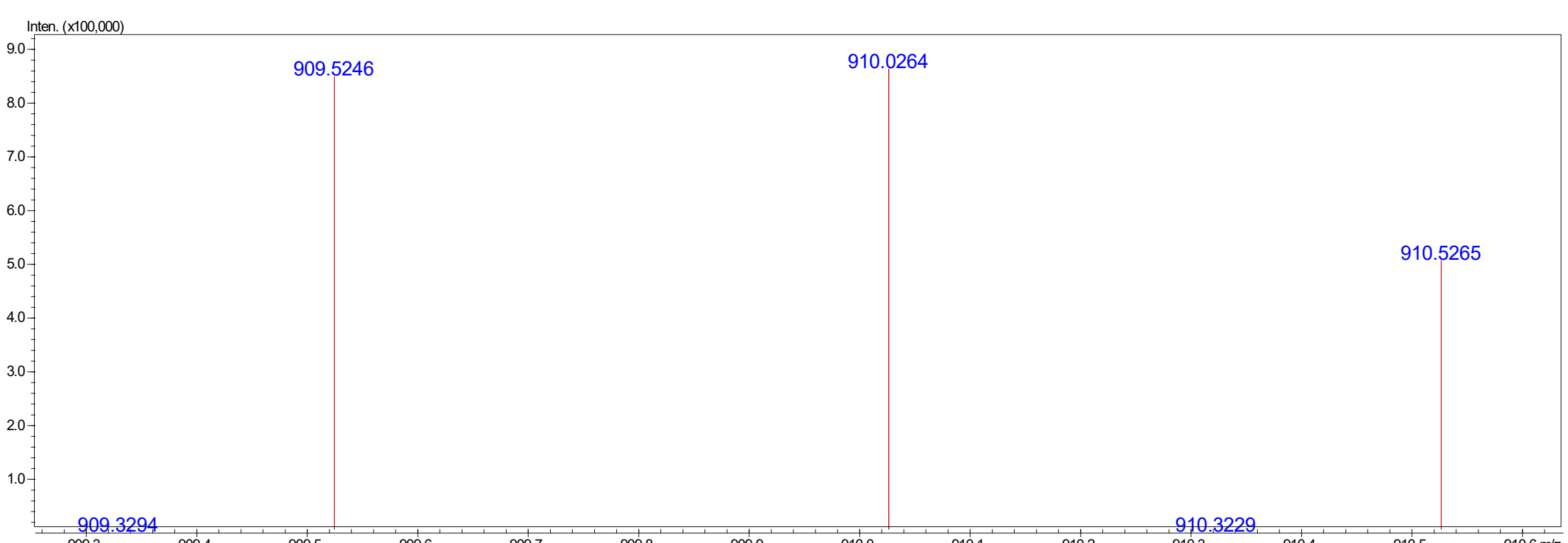

2a - detailed $[M+3H]^{3+}$:

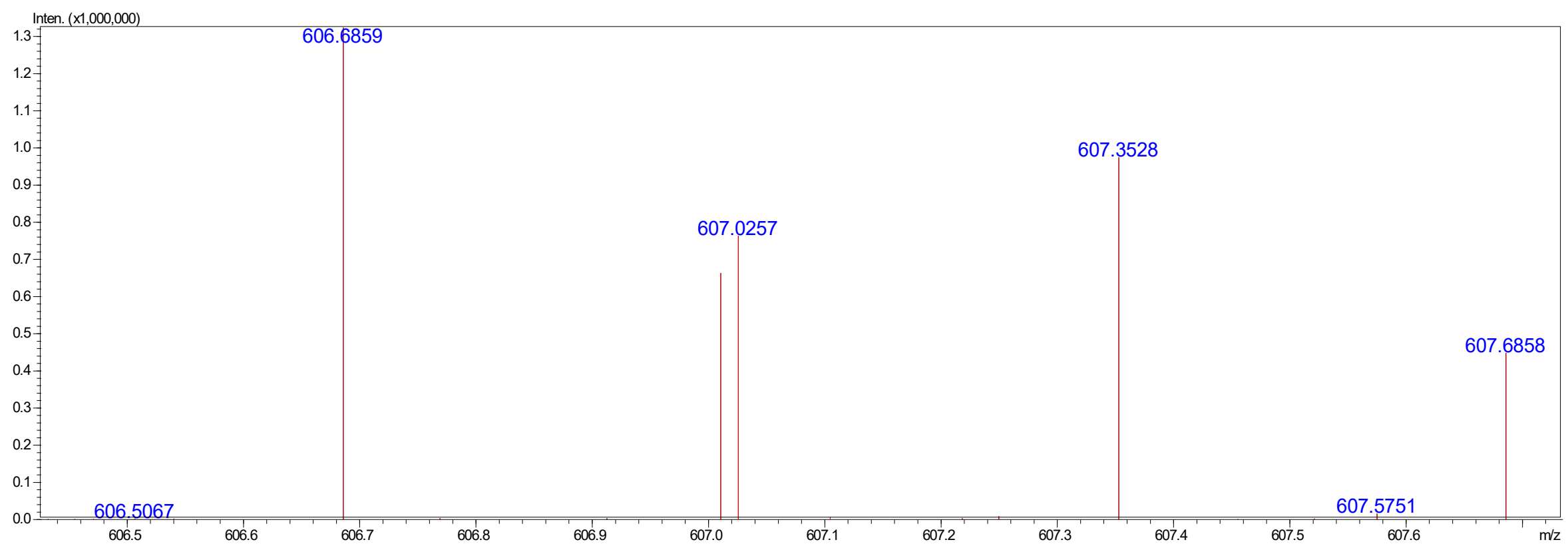

Metronidazole-conjugated peptide 1b:

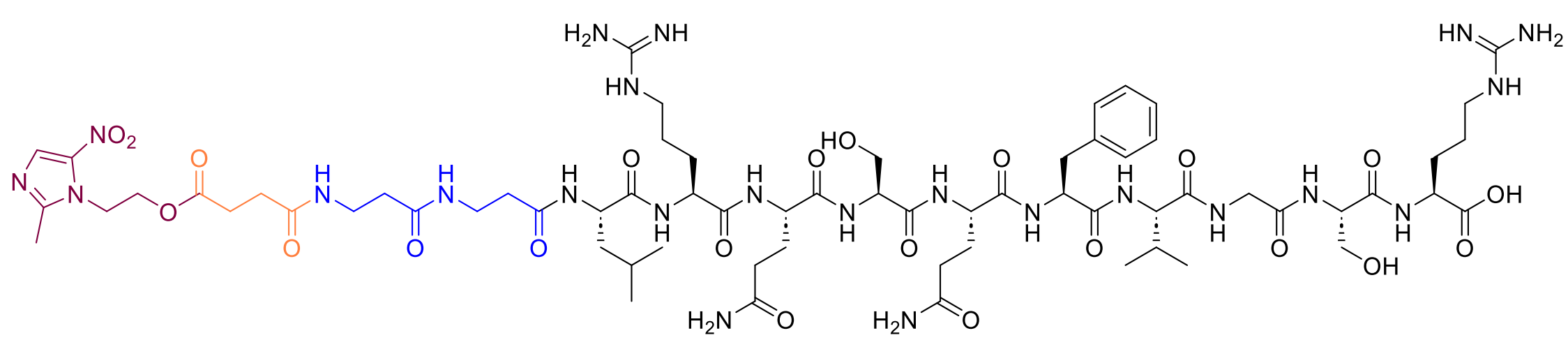

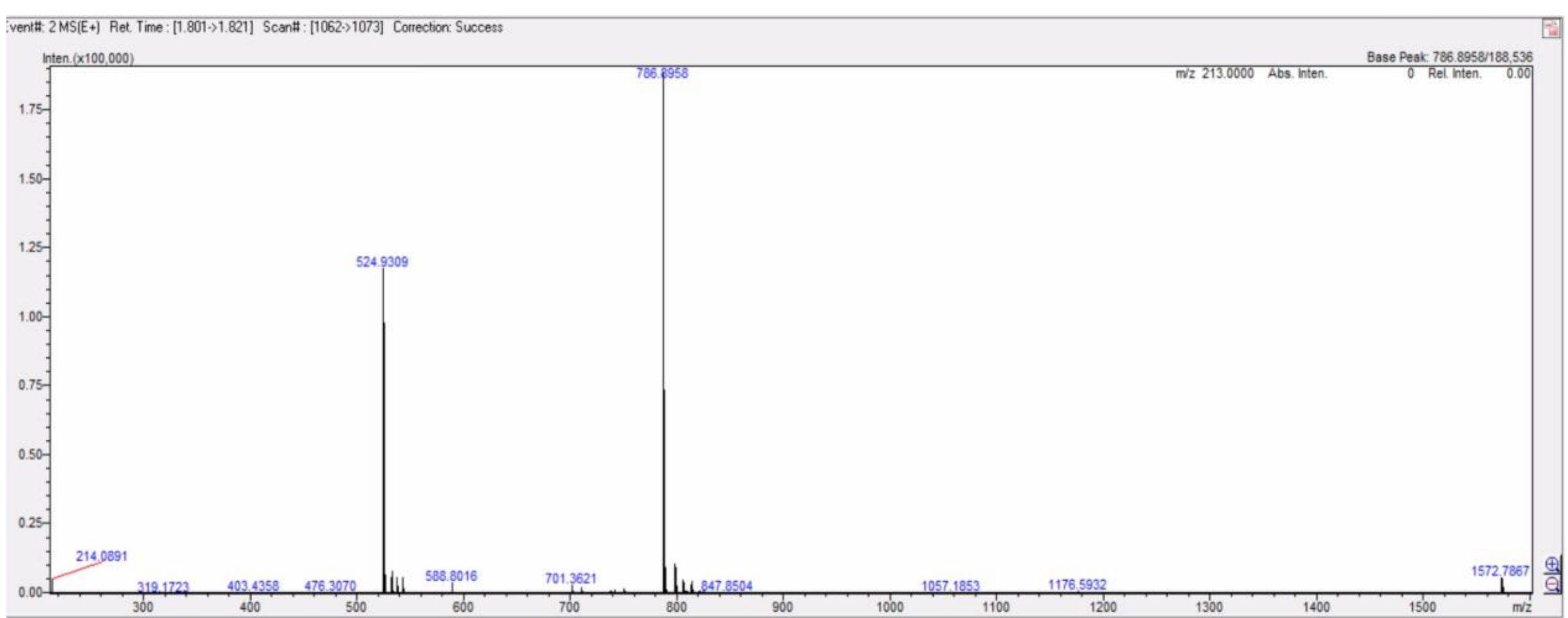

Metronidazole-conjugated peptide 2b:

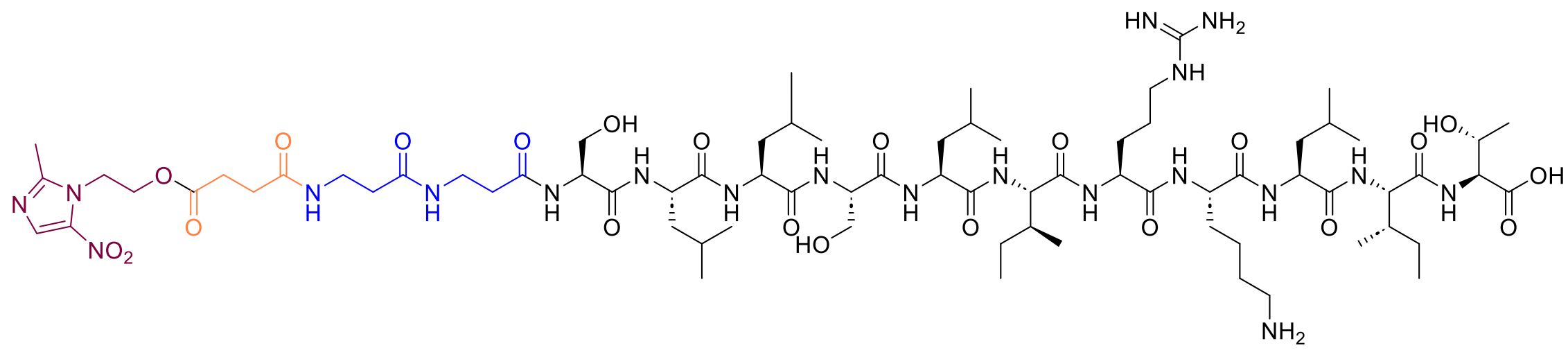

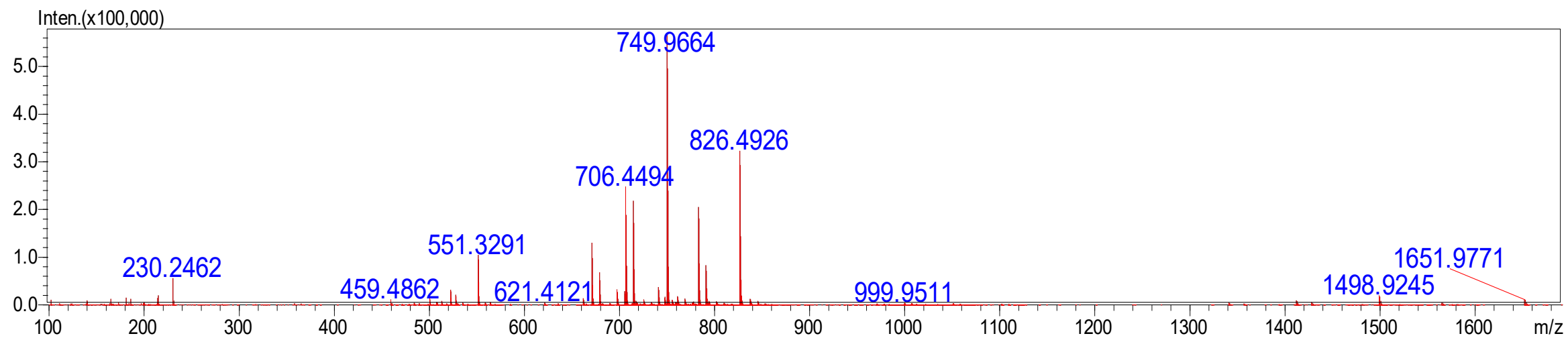

Molecular dynamic simulations

Table S1. Intramolecular and with water hydrogen bonding of the studied peptides

Peptide	Intramolecular hydrogen bonds (no/ns)	Hydrogen bonds with water (no/ns)	Average RMSD peptide moiety (nm)	Radius of gyration of whole structure (nm)	Solvent accessible surface area (nm^2)	Free energy of solvation (kJ/mol)
**1**	2.14 ± 1.57	42.02 ± 4.34	0.42 ± 0.14	0.72 ± 0.08	15.49 ± 1.11	-2.40 ± 1.62
**1a**	2.85 ± 1.96	44.13 ± 5.53	0.52 ± 0.12	0.81 ± 0.11	19.55 ± 1.88	-3.52 ± 2.02
**1b**	3.09 ± 1.90	48.74 ± 5.06	0.59 ± 0.17	0.84 ± 0.13	18.80 ± 1.75	-0.13 ± 1.15
**2**	5.28 ± 1.74	30.66 ± 3.35	0.26 ± 0.10	0.62 ± 0.04	14.40 ± 0.87	1.94 ± 2.25
**2a**	6.23 ± 1.84	38.43 ± 4.18	0.43 ± 0.15	0.78 ± 0.10	19.18 ± 1.38	0.71 ± 1.90
**2b**	4.69 ± 1.56	40.19 ± 4.06	0.47 ± 0.14	0.80 ± 0.09	18.59 ± 1.38	-2.10 ± 1.53

Table S2. The average energy of solvation of peptides **1, 1a, 1b, 2, 2a, 2b** (kJ/mol)

Type of interaction	1	1a	1b	2	2a	2b
**Coulombic**	-1606.85 ± 168.63	-1467.94 ± 153.49	-1664.61 ± 175.09	-1504.05 ± 105.43	-1661.95 ± 170.59	-1549.91 ± 145.26
**Lennard–Jones**	-226.43 ± 37.04	-380.43 ± 46.69	-329.85 ± 43.96	-194.33 ± 35.80	-324.39 ± 41.33	-316.03 ± 43.06

Table S3. The average energy of intramolecular interaction of peptides **1, 1a, 1b, 2, 2a, 2b** (kJ/mol)

Type of interaction	1	1a	1b	2	2a	2b
**Coulombic**	-1683.16 ± 102.65	-750.92 ± 91.87	-1864.49 ± 100.73	-2253.95 ± 68.69	-1920.54 ± 89.90	-2414.75 ± 89.80
**Lennard–Jones**	-132.93 ± 20.70	-235.28 ± 40.22	-201.18 ± 32.07	-200.53 ± 21.17	-283.42 ± 26.15	-244.11 ± 29.13

Table S4. The average distance between the first and the last α carbon from the peptides **1, 1a, 1b, 2, 2a, 2b** (nm)

**Peptide**	**1**	**1a**	**1b**	**2**	**2a**	**2b**
**Distance ± SD**	1.43 ± 0.47	1.39 ± 0.46	1.25 ± 0.45	1.48 ± 0.23	1.53 ± 0.21	1.62 ± 0.23

Table S5. The average distance between the carbon atom from position 3 of norfloxacin (for **1a** and **2a**) and the nitrogen atom from position 1 of metronidazole (for **1b** and **2b**) and the first and the last (in blue) α carbons from the peptides **1a, 1b / 2a, 2b (nm)**

**Distance to**	**1a**	**1b**	**2a**	**2b**
**The first α carbon**	1.04 ± 0.37	1.19 ± 0.42	1.19 ± 0.28	1.17 ± 0.34
**The last α carbon**	1.37 ± 0.51	1.65 ± 0.56	1.63 ± 0.51	1.84 ± 0.59

Table S6. The clustering analysis of the poses during the 200 ns molecular dynamics simulation (% of total poses)

**Cluster**	**1**	**1a**	**1b**	**2**	**2a**	**2b**
**1st**	6.69	6.88	5.48	80.30	27.20	6.04
**2nd**	5.17	4.62	2.49	6.39	5.02	4.61
**3rd**	4.97	2.85	2.20	4.52	3.88	4.48
**4th**	4.94	2.68	1.90	2.57	3.00	3.56
**5th**	4.87	2.37	1.90	1.29	2.91	3.47
